# Human umbilical mesenchymal stem cell-derived mitochondria transplantation suppresses sFLT-1 secretion by regulating calcineurin-NFAT-dependent pathways in angiotensin II-induced preeclampsia rats

**DOI:** 10.1186/s13287-026-04930-9

**Published:** 2026-02-13

**Authors:** Hui Xing Cui, Jun Xian Liu, Young Cheol Kang, Kyuboem Han, Hong Kyu Lee, Chun-Hyung Kim, Yin Hua Zhang

**Affiliations:** 1 Department of Medical Cosmetology and Plastic Surgery, Shandong Provincial Maternal and Child Health Care Hospital Affiliated to Qingdao University, Jinan, Shandong China; 2 Department of Physiology & Biomedical Sciences, Ischemic/Hypoxic Disease Institute, Seoul National University College of Medicine, Seoul, Republic of Korea; 3 Paean Biotechnology, Inc., 5 Samil-Daero 8-Gil, Jung-Gu, Seoul, 04552 Republic of Korea; 4 Yanbian University Hospital, Yanji, China

**Keywords:** Mitochondrial transplantation, Human umbilical mesenchymal stem cell, Preeclampsia, sFlt-1, PlGF, sFlt-1/PlGF, ROS, Trophoblast cells, Calcineurin-NFAT-dependent pathways

## Abstract

**Background:**

Mitochondrial transplantation (Mito-T) is a novel therapeutic strategy for ischaemic cardiovascular diseases. This study aimed to test the efficacy of human umbilical mesenchymal stem cell-derived mitochondrial transplantation (Mito-T) on preeclampsia (PE).

**Methods:**

PE was induced in Sprague–Dawley pregnant rats by infusing angiotensin II (Ang II) starting on gestation day 8 (GD 8). Mito-T (100 μg/μl) was injected via the jugular vein on GD 14.

**Results:**

On GD 20, PE rats exhibited high blood pressure, kidney and placental vascular abnormalities, reduced placental and foetal weights, foetal crown-rump lengths. Mito-T was predominantly distributed in the kidneys, uterus, and placenta of PE rats. Mito-T reversed clinical manifestations of PE, restored placental vascular abnormalities, and reduced serum sFLT-1 levels and the sFLT-1/PlGF ratio. In placental mitochondria, Mito-T increased protein levels of complexes (I‒V), improved mitochondrial membrane potential, ATP synthase, citrate synthase activities, and biogenesis markers (PGC-1α, TFAM, and NRF1), and reduced reactive oxygen species production. Mito-T increased mitochondrial fusion proteins (OPA1, MFN1, and MFN2) in the placenta, whereas fission (DRP1 and FIS1) and mitophagy (PINK, BNIP3, BNIP3L, and FUNDC1) proteins were reduced. In placental tissue, primary trophoblast cells, and the Bewo cell line, Mito-T reduced the mRNA and protein levels of sFLT-1 and attenuated the calcineurin-NFAT pathways elevated by PE or Ang II.

**Conclusions:**

This study demonstrates that Mito-T reverses the pathological phenotypes of PE rats by improving placental mitochondrial activity and suppressing trophoblast-derived sFLT-1 production. These findings provide proof-of-concept evidence that Mito-T could serve as a potential therapeutic strategy for reducing maternal and foetal risks in patients with PE.

**Graphical Abstract:**

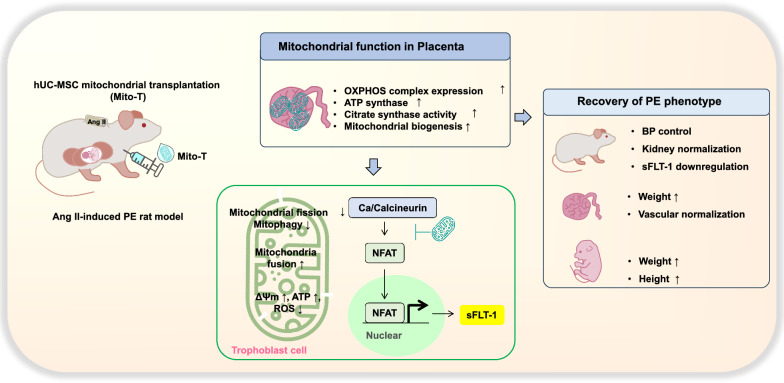

**Supplementary Information:**

The online version contains supplementary material available at 10.1186/s13287-026-04930-9.

## Introduction

Pre-eclampsia (PE), a pregnancy-specific multisystem functional disorder characterised by abnormal placental function, oxidative stress imbalance, and vascular endothelial injury, seriously threatens maternal and infant health [1, 2]. Clinical manifestations of PE include elevated blood pressure (BP) and/or kidney dysfunction (proteinuria) at 20 weeks of gestation. If left untreated, maternal systemic complications may develop into HELLP syndrome, pulmonary oedema, seizures, and fatal arrhythmias, affecting both mothers and babies [3]. Currently, clinical management remains focused on symptomatic support and pregnancy termination, lacking effective etiological interventions. Therefore, there is an urgent need for new treatment strategies to fundamentally intervene in treatment and effectively control the occurrence and development of PE [4].

The pathophysiological mechanisms of preeclampsia are complex and involve multiple factors such as abnormal placental formation, vascular endothelial dysfunction, and inflammatory response [5]. Of these, placental trophoblast dysfunction is a key factor in PE [6, 7]. Trophoblasts are essential for the exchange of nutrients and gases between the mother and foetus, and their impaired function can lead to insufficient blood supply to the foetus, triggering a series of pathophysiological changes in the mothers [8, 9]. sFLT-1 is an antiangiogenic factor whose expression is increased in the serum of patients with PE. By binding to vascular endothelial growth factor, it inhibits angiogenesis and exacerbates vascular endothelial dysfunction and hypertension [10–12].

Stem cells have attracted substantial attention in the field of regenerative medicine owing to their self-replication and multidirectional differentiation potential [13–15]. Various types of stem cells, such as mesenchymal stem cells (MSCs) and endometrial stem cells, potentially repair damaged tissues and regulate immune responses [13–15]. Mitochondria are the energy factories of cells and are involved in cellular energy metabolism, signal transduction, and apoptosis [16, 17]. Mitochondrial dysfunction is associated with the occurrence and development of various diseases [17, 18].

Currently, mitochondrial transplantation (Mito-T) is emerging as a new therapy for ischaemia-related diseases that restores cellular energy metabolism and function by transplanting healthy mitochondria into damaged cells [18–21]. Local and intravascular injections of autologous and heterologous mitochondria are beneficial to patients with myocardial infarction, pre-ischaemia, immediately after ischaemia, and during reperfusion [22]. Mito-T applications extend from diseases of the heart and brain (stroke) to the kidney, lung, liver, skeletal muscle, spinal cord, and sepsis [22]. Reported mitochondria sources include skeletal muscle, liver, heart, and platelets [22]. The efficacy of mitochondria from these sources is comparable; however, more stable sources may be advantageous. Recently, mitochondria from human umbilical cord mesenchymal stem cells (hUC-MSCs) are integrative and functionally stable [23], and Mito-T from hUC-MSCs improves the survival of lipopolysaccharide (LPS)-injected sepsis in mice [23]. A phase I/IIa clinical trial for refractory polymyositis/dermatomyositis indicated therapeutic potential, opening the possibility that hUC-MSC-derived mitochondria may serve as a useful source for treating a wide range of human diseases. Until recently, Mito-T using hUC-MSC-derived mitochondria for placental dysfunction and PE remained unexplored.

In this study, the effects of Mito-T from hUC-MSCs were tested in an Ang II-induced PE rat model. Maternal and foetal phenotypes, along with the vascular structure of major maternal organs, were evaluated before and after Mito-T treatment. In particular, placental structure, mitochondrial function, and dynamics were examined, together with the molecular mechanisms mediating the functional recovery of placental mitochondria and vasculature in PE. Our results provide convincing evidence indicating the beneficial effects of Mito-T on maternal organs and placental mitochondrial function in PE.

## Materials and methods

This study was conducted in accordance with the ARRIVE 2.0 guidelines.

### Rat model of PE and Mito-T administration

**Fig. 1:**
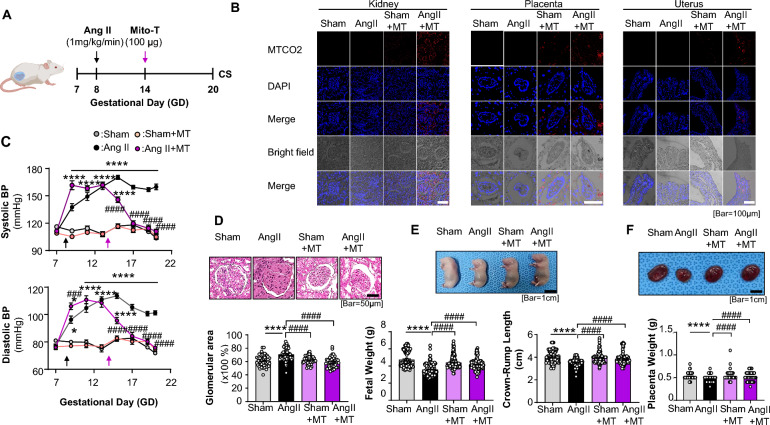
Mito-T restored clinical manifestations of Ang II-Induced PE Rats. **A** Ang II was infused via osmotic minipump (1 μg/kg/min) at GD 8 in the Ang II group. hUC-MSC-derived mitochondria (100 μg/μl) were injected into the jugular vein (Mito-T groups) on GD 14, and rats were sacrificed at GD 20. **B** Distribution of hUC-MSC mitochondria in the kidney, uterus, and placenta tissues of rats in the Sham and Ang II groups. **C** Comparison of systolic and diastolic blood pressure in the Sham, Ang II, Sham + MT, and Ang II + MT groups. Each group consists of six rats. **D** Representative H&E staining images of the kidney. The glomerular area was compared between the four groups. **E** Images of foetal rats from the sham, Ang II, sham + MT, and Ang II + MT groups. Foetal weight and crown-rump length were compared between the four groups. (foetal number: Sham 144, Ang II 149, Sham + MT 157, Ang II + MT 124). **F** Representative images of placentas and comparison of placental weight across the four groups. (Placenta number: Sham 138, Ang II 159, Sham + MT 145, Ang II + MT 106). Values are expressed as mean ± SEM. *P < 0.05, **P < 0.005, ****P < 0.0001. * Sham group vs. Ang II group or Sham + MT vs. Ang II + MT; # indicates comparison between the Ang II, Sham + MT, and Ang II + MT groups

All animal experimental procedures were performed in accordance with the Guide for the Care and Use of Laboratory Animals and were approved by the Institutional Animal Care and Use Committee of the Laboratory Animal Centre, Seoul Medical University, South Korea [SNU-220724-1-1]. Twenty-four pregnant Sprague–Dawley rats (gestation day, GD 7, KOATECH, Korea) were randomly categorized into four groups: sham, Ang II, sham + MT, and Ang II + MT. Ang II was infused through an osmotic minipump (1 μg/kg/min) on GD 8 (Ang II group) [24]. hUC-MSC mitochondria (100 μg/μl) were injected into the jugular vein on GD 14 (Mito-T groups), and rats were sacrificed at GD 20 (Fig. 1A). Kidney and placenta tissues were collected on the day of euthanasia. BP was measured on GD 7, 9, 11, 13, 15, 17, 19, and 20 using a non-invasive blood pressure measurement device, and the averaged data from three cycles of measurements were collected for data analysis. Pregnant rats were anaesthetised with sodium pentobarbital (50 mg/kg) via intraperitoneal injection before euthanasia by CO_2_ inhalation.

### Quantitative analysis of the basal decidua of the placenta

Haematoxylin and eosin (H&E)-stained sections from four placental samples per group (three consecutive sections per sample) were analysed using Image J software. The ratio of basalis to total placental area (%) was used as the quantitative indicator.

### Isolation of mitochondria from hUC-MSC

hUC-MSCs were obtained by primary culture of umbilical cord from a healthy pregnant woman with informed consent. The study was approved by Public Institutional Review Board (IRB) designated by Ministry of Health and Welfare, Korea (IRB No. P01-202002-31-008). Mitochondria were isolated from membrane-disrupted hUC-MSC using differential centrifugation, with quality and purity strictly controlled based on previously established clinical and nonclinical protocols [25, 26].

Briefly, hUC-MSC cells were harvested and depressurised in SHE buffer (0.25 M Sucrose, 20 mM HEPES, 2 mM EGTA, 0.1% bovine serum albumin [BSA], pH 7.4) using nitrogen cavitation (Parr Instrument, USA). After centrifugations (2000 × g for 10 min at 4 °C and the supernatant at 12,000 × g for 15 min, 4 °C), the mitochondria pellet was washed twice with 500 μl SHE buffer, followed by centrifugation at 20,000 × g for 10 min at 4 °C. The purified mitochondrial pellet was resuspended in 100 μl suspension buffer (300 mM Trehalose), a known cryoprotectant [27]. Isolated mitochondria were rapidly frozen in 300 mM Trehalose solution and stored frozen until the day of the experiment. The frozen mitochondria were thawed and used immediately, avoiding repeated freeze–thaw cycles. Protein concentrations were quantified using a bicinchoninic acid assay.

### H&E staining and immunohistochemistry

Kidney and placenta tissues were fixed in 4% paraformaldehyde, processed through wax embedding, sectioned, and stained. Subsequently, the tissues were transferred to 70% ethanol solution for 24 h at 20‒22 °C. The samples were embedded in paraffin, cut into 4 μm sections, and stained with H&E and toluidine blue. Slides were visualised using Aperio ImageScope software.

Paraffin-embedded tissue sections were rehydrated, subjected to antigen retrieval using tris–EDTA (pH 8.0) or 10 mM sodium citrate buffer (pH 6.0) at 95 °C for 10 min, and blocked with goat serum for 20 min. Sections were incubated with primary antibody (eNOS; 1:200, BD Biosciences) at 4 °C, followed by incubation with secondary antibody (Anti-IgG: 1:200, Cell Signalling) at room temperature for 30 min. Samples were processed using a DAB substrate kit (Sigma Aldrich, USA) based on the instructions of the manufacturer. To track Mito-T, rehydrated tissue sections were stained with an antibody against human mitochondrial cytochrome c oxidase subunit II (1:1000, rabbit polyclonal antibody, Abcam). After washing with phosphate-buffered saline (PBS), sections were incubated with fluorescence-tagged rabbit secondary antibodies (Invitrogen, USA) for visualisation. The stained samples were mounted in VECTASHIELD with DAPI mounting solution and imaged using a confocal microscope (FluoView FV3000, Olympus, Japan).

### Enzyme-linked immunosorbent assay (ELISA) experiments

sFLT-1 levels in maternal serum samples were determined using ELISA kits (MyBiosource Inc., San Diego, CA, USA; CAT#MBS725733) based on the instructions of the manufacturer.

### RNA preparation and quantitative real-time PCR

Total RNA was extracted from collected tissues using Qiazol Lysis Reagent (Cat.no.79306, QIAGEN). The concentration of the purified RNA was measured using a NanoDrop spectrophotometer (Thermo Fisher Scientific). Primer sequences are provided in Data Supplement 1. Briefly, cDNA was amplified with SYBR green (BIONEER 2XGreenStarqPCRMaster MIX) using a real-time PCR system (Applied Biosystems 7500) under the following conditions: 95 °C for 3 min, 40 cycles at 95 °C for 10 s, 55 °C for 3 s, 95 °C for 10 s, and 65 °C for 5 s. Specificity of the amplification products was assessed by melting curve analysis. Relative gene expression was calculated using the comparative threshold (Ct) method (2^−△△Ct^).

### Western blotting analysis

Proteins were extracted from placental tissues using lysis buffer containing 150 mM NaCl, 50 mM Tris–HCl, 1 mM EDTA, 1% Triton X-100, and phosphatase inhibitor cocktail (Roche), pH 7.4. The lysates were boiled at 95 °C for 10 min, fractionated by SDS/PAGE, and transferred to PVDF membranes in 25 mM Tris, 192 mM glycine, 0.01% SDS, 20% methanol. Membranes were blocked in 1X TBS containing 1% Tween-20 and 5% BSA (blocking solution) for 1 h at room temperature with gentle rocking. Membranes were incubated overnight at 4 °C with primary antibodies, followed by secondary antibodies (Data Supplement 2). Blots were developed using enhanced chemiluminescence plus western blotting detection reagents (Amersham Biosciences).

### ATP synthesis activity assay

A reaction mixture containing 500 μM ADP and 5 μg mitochondria was prepared at room temperature. ATP production was measured every 2.5 s for 20 min using luminescence detection on a SYNERGY HTX multi-mode reader (BioTek, USA).

### Citrate synthesis activity assay

Citrate synthase activities were assessed in isolated mitochondria as described previously.^21^ Briefly, after adding 2 μg of mitochondria and 10 mM acetyl CoA, the reaction was initiated by adding 5,5’-Dithiobis (2-nitrobenzoic acid) (Sigma), and the change in absorbance was recorded at 450 nm every 1 min for 20 min using SYNERGY HTX multi-mode reader (BioTek, USA).

### Mitochondrial membrane potential (MMP) measurement

MMP was assessed by incubating mitochondria (10 μg) with tetramethylrhodamine methyl ester (TMRM, 100 nM) in mitochondrial assay solution (MAS buffer: 220 mM D-Mannitol, 70 mM sucrose, 10 mM KH_2_PO_4_, 5 mM MgCl_2_, 2 mM HEPES, 1 mM EGTA, 0.2% BSA, pH 7.2 adjusted with KOH) for 20 min at 28 °C. MMP was measured in TMRM-incubated mitochondria in the presence of metabolic substrates (5 mM pyruvate, 5 mM malate, 5 mM glutamate, 4 mM ADP, and 5 mM succinate) in a 96-well plate (excitation at 540 nm and emission at 580 nm). All plate setup, scanning, and analysis were conducted based on the protocols of the manufacturer.

### Reactive oxygen species (ROS) measurement

Mitochondrial ROS levels were determined using the Amplex™ Red Hydrogen Peroxide/Peroxidase Assay Kit (Invitrogen, A22188). In a 100 μl working solution containing 100 μM Amplex® Red reagent and 0.2 U/mL horseradish peroxidase, 10 μl of mitochondria (1 μg/μl) were added and supplemented with MAS buffer (220 mM D-Mannitol, 70 mM sucrose, 10 mM KH_2_PO_4_, 5 mM MgCl_2_, 2 mM HEPES, 1 mM EGTA, 0.2% BSA W/V, pH 7.2 adjusted with KOH) to a final volume of 155 μl. The mixture was incubated in the dark (at 37 °C) for 10 min. Substrates (45 μl; 5 mM pyruvate, 5 mM malate, 5 mM glutamate, 4 mM ADP, and 5 mM succinate) were then added, and ROS was measured in a 96-well plate using fluorescence detection (excitation at 530 nm and emission at 590 nm).

### Isolation of trophoblast cells from the placenta

Trophoblast cells were isolated from the placenta as previously described [27]. Briefly, placenta tissue was cut into small pieces (2 × 2 mm) and washed thoroughly with PBS (0‒4 °C) to remove blood. Tissue pieces were minced in Dulbecco’s Modified Eagle Medium (DMEM; 5 ml/g) and digested with 1 mg/ml Dispase II, 0.5 mg/ml collagenase I, and 0.1 mg/ml DNase I at 37 °C for 20 min per cycle, repeated three times. An equal volume of DMEM, containing 10% foetal bovine serum (FBS), 100 U/ml penicillin, and 100 μg/ml streptomycin, was added to the tissue mixture to stop enzyme degradation. The mixture was filtered sequentially through 100 μm and 40 μm strainers to remove tissue debris. Trophoblast cell suspension was collected and centrifuged at 350 g for 10 min at 4 °C. The resulting cell pellets were resuspended in 5 ml DMEM and layered on top of a preformed Percoll gradient (65%, 55%, 50%, 45%, 35%, 30%, and 25%). The gradient was centrifuged at 30,000 × g at 4 °C for 30 min. The layer between the 45% and 35% (density 1.050‒1.060 g/ml) was collected, washed with DMEM, and centrifuged at 350 × g for 10 min at 4 °C. The final pellets were resuspended in PBS solution for further experiments.

### Nuclear protein extraction

Nuclear proteins were extracted using a nuclear extraction kit (Lot: WB 317789, Thermo Scientific, USA) based on the instructions of the manufacturer. Briefly, 20 mg of placental tissue was homogenized in 200 μl ice-cold buffer A (containing dithiothreitol (DTT) and protease inhibitor) and centrifuged at 16,000 × g for 10 min at 4 °C. The pellets were resuspended in 11 μl buffer B and centrifuged at 16,000 × g for 5 min at 4 °C. The supernatant was used as the cytosolic fraction of the placental cells. To extract nuclear proteins, the pellets were incubated on ice for 40 min after adding 100 μl buffer C (with DTT and protease inhibitor), vortexing for 15 s every 10 min. The mixture was then centrifuged at 16,000 × g for 10 min at 4 °C, and the supernatant was collected as the nuclear fraction.

### Culture of the trophoblast BeWo cell line

The trophoblast cell line BeWo was obtained from the Korean Cell Line Bank (KCLB No. 10098). BeWo cells were maintained in DMEM (Thermo Fisher) supplemented with FBS (10%). The cell lines were divided into four groups: Sham, Sham + MT (100 μg/ml), Ang II (1 μM), and Ang II + MT. Following 24-h incubation, the cells were collected for subsequent experiments.

### Imaging of well plates using confocal laser scanning microscopy

Cell lines were divided into four groups: sham, sham + MT (100 μg/ml), Ang II (1 μM), and Ang II + MT. MT-GFP (green) was provided by Paean Biotechnology. Cells were collected after 24 h of incubation. For MMP analysis, cells were incubated with 400 nM TMRM at 37 °C for 30 min, and unbound dye was washed off. Stained cells were maintained in 5 nM TMRM in 5% phenol red-free RPMI medium for imaging. Cells seeded in 96-well optical bottom plates were continuously scanned using a Leica TCS SP8 confocal microscope (Leica Microsystems, Wetzlar, Germany) equipped with a supercontinuum white-light laser. TMRM fluorescence was recorded with emission at 600 ± 25 nm under 562 nm excitation.

### Statistical analysis

Statistical analyses were performed using GraphPad Prism 8 software (GraphPad Software). Data are presented as means ± SEM or median (minimum to maximum). One-way ANOVA or independent t-tests were used as appropriate. A *P* < 0.05 was considered statistically significant. All experiments were repeated three times.

## Results

### Mito-T restores clinical manifestations in Ang II-induced PE rats

First, we examined Mito-T distribution in the organs of sham and Ang II-treated rats on GD20 via immunofluorescence staining of MT-CO2 proteins in hUC-MSCs. Mito-T was detected in the glomerular and collecting tubules of the kidney, epithelial interstitial spaces of the uterus, and labyrinth/foetal vessels of the placenta in both Sham and Ang II-treated rats, with noticeably higher intensity in the Ang II group (Fig. 1B).

Ang II infusion from GD8 caused significant increases in systolic and diastolic BPs (mmHg) from GD9 to GD20. Peak BP values were as follows: Sham, systolic 112.28 ± 12.72, diastolic 81.86 ± 8.95; Ang II, systolic 156.75 ± 11.58, diastolic 113.69 ± 12.06 (sham vs. Ang II *P* < 0.0001, *P* < 0.0001) (Fig. 1C). Mito-T administration at GD14 gradually reduced the BP of Ang II rats until GD20 without affecting BP in Sham rats (Sham + MT: systolic BP 102.40 ± 12.51, diastolic BP 74.57 ± 7.67; Ang II + MT: systolic BP 107.47 ± 11.15, diastolic BP 75.80 ± 6.39, Sham vs. Ang II *P* < 0.0001, Ang II vs. Ang II + MT *P* < 0.0001) (Fig. 1C). Across GD15‒20, BP was significantly higher in Ang II than in Ang II + MT mice (systolic BP, *P* < 0.0001; diastolic BP, *P* < 0.0001) (Fig. 1C).

Subsequently, we examined histological changes in the maternal kidney cortex induced by Ang II and the effects of Mito-T. H&E staining showed that glomerular capillaries were reduced and glomerular diameter was increased in the Ang II group, resulting in a significantly higher glomerular diameter/capsule volume (glomerular diameter/capsule volume: Sham, 61.12%; Ang II, 70.58%; *P* < 0.0001) (Fig. 1D). Mito-T restored glomerular capillaries and normalized glomerular diameter, reducing the glomerular diameter/capsule volume (Sham, 61.92%; Ang II, 58.66%; *P* < 0.0001) (Fig. 1D).

The offspring of the four groups were examined to investigate the effects of Mito-T. Foetal size was significantly smaller in the Ang II group; however, this reduction was reversed by Mito-T (Fig. 1E). Quantitatively, foetal weight was significantly reduced in Ang II rats and restored by Mito-T (foetal weight in g: Sham, 4.74 ± 0.95, n = 144; Sham + MT, 4.4 ± 0.72, n = 157; Ang II, 3.58 ± 0.66, n = 149; Ang II + MT, 4.16 ± 0.64, n = 124; *P* < 0.0001 for Sham vs. Ang II and Ang II vs. Ang + MT) (Fig. 1E). Similarly, foetal crown-rump length was shorter in the Ang II group but preserved by Mito-T treatment (foetal crown-rump length in cm: Sham: 4.07 ± 0.05, n = 138; Ang II: 3.50 ± 0.03, n = 140; Sham + MT: 3.99 ± 0.05, n = 118; Ang II + MT: 4.00 ± 0.05, n = 114, *P* < 0.0001 between Sham *vs*. Ang II and Ang II *vs.* Ang II + MT) (Fig. 1E).

Mito-T also affected placental weight. Placental weight was lower in the Ang II group; however, this decrease was reversed by Mito-T (placental weight in g in Sham: 0.53 ± 0.007 g, n = 105; Ang II: 0.47 ± 0.008 g, n = 111; Sham + MT: 0.53 ± 0.007 g, n = 98; Ang II + MT: 0.52 ± 0.010 g, n = 78; Sham vs. Ang II, *P* < 0.0001; Ang II vs. Ang II + MT: *P* < 0.0001) (Fig. 1F).

These results provide direct evidence that Ang II induces the PE phenotype in rats, with clinical manifestations such as maternal hypertension, renal abnormalities, and foetal growth restriction. Mito-T reversed all these effects, demonstrating its potential for preventing PE.

### Mito-T administration on the vasculature of the placenta

Mito-T distribution in the placenta and improvements observed in PE offspring suggest a preventive role. We examined the structural and functional bases of the placenta following Mito-T treatment.

**Fig. 2:**
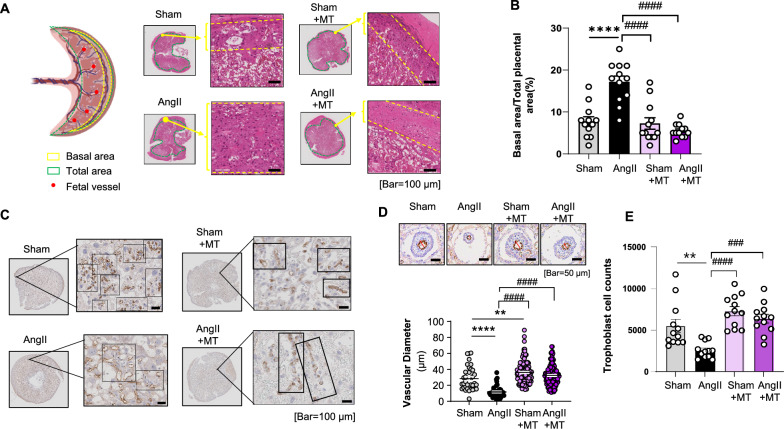
Placental vasculature and trophoblasts in Ang II-induced PE rats and their responses to Mito-T. **A** Schematic diagram of the rat placenta. H&E staining showed changes in the dimensions of the basal zone. **B** Basal region of the placenta is significantly expanded in the PE group (Sham vs. Ang II, *p* < 0.0001), which was restored after Mito-T treatment (Ang II vs. Ang II + MT, *p* < 0.0001; Sham vs. Sham + MT, *p* = ns; Sham vs. Ang II + MT, *p* = ns, Ang II vs. Sham + MT, *p* < 0.0001). (Each group includes 12 regions). **C** Immunohistochemistry images showing changes in placental vasculature in the labyrinthine zone (placental barrier) and placental chorionic foetal blood vessels in PE, with recovery following Mito-T. **D** Immunohistochemistry images with eNOS staining of endothelium showing differences in foetal blood vessel diameter between the Sham and Ang II groups with and without Mito-T. Vascular diameter was reduced significantly in PE and restored by Mito-T (Sham vs. Ang II, *p* < 0.0001; Sham vs. Sham + MT, *p* = 0.0061; Ang II vs. Ang II + MT, *p* < 0.0001, Ang II vs. Ang II + MT, *p* < 0.0001). (Number of diameters measured per group: Sham 38, Ang II 43, Sham + MT 121, Ang II + MT 131). **E** Quantification of trophoblast cells (syncytiotrophoblasts and cytotrophoblasts) based on immunohistochemistry images of the foetal blood vessels. PE caused a significant reduction in trophoblast number (Sham vs. Ang II **, *p* = 0.0035), which was recovered with Mito-T. The Sham + MT and Ang II + MT groups showed significantly higher trophoblast numbers than the Ang II group (Ang II vs. Sham + MT ####, *p* < 0.0001; Ang II vs. Ang II + MT ###, *p* = 0.0001). (Each group consists of 12 regions, n = 12). Values are expressed as mean ± SEM. *P < 0.05, **P < 0.005, ****P < 0.0001. * Sham group vs. Ang II group or Sham + MT vs. Ang II + MT; # indicates comparison between the Ang II, Sham + MT, and Ang II + MT groups

H&E staining results showed that the basal layer of the decidua in the placenta of Ang II-treated rats was significantly widened, whereas it returned to its normal width after Mito-T treatment (Fig. 2A).

Quantitative analysis of the basal area relative to total placental area in H&E staining showed significant expansion of the basal area in the PE group (Sham vs Ang II, *p* < 0.0001), and Mito-T restored the changes to the normal range (Ang II vs Ang II + MT, *p* < 0.0001; Sham vs Sham + MT, *p* = ns) (Fig. 2B).

Immunohistochemistry with eNOS protein further demonstrated that the vasculature in the labyrinth zone near the junctional zone, as well as foetal blood vessels near the chorion, was significantly affected in PE compared to those in Sham. In the Sham group, capillaries near the junctional zone form clusters around trophoblasts, suggesting a close interconnection that likely contributes to a solid placental barrier (Fig. 2C). In contrast, PE placentas exhibited collapsed capillary structures and significantly enlarged sinus cavities, leading to loosely arranged trophoblast cells. In the labyrinth trophoblast septa, the continuity of syncytiotrophoblast maintains the placental barrier [28]. Therefore, the changes in PE were presumably due to the ischaemic microenvironment secondary to thickening of the basal zone and impaired spiral vessel remodelling, indicating a compromised placental barrier in PE (Fig. 2C). Mito-T restored vascular clustering, promoted close arrangement of trophoblast cells, and reduced sinus cavity enlargement in the labyrinth zone, suggesting potential restoration of the placental barrier.

Changes in foetal vasculature near the chorion were observed and quantified (Fig. 2D). The diameter of foetal blood vessels was significantly reduced in PE (Sham 28.39 ± 14.21 μm, Ang II 11.72 ± 7.02 μm; Sham vs. Ang II, *P* < 0.0001). After Mito-T administration, the diameter of foetal blood vessels in the chorion was significantly increased (Sham + MT: 36.16 ± 13.61 μm, Ang II + MT: 32.42 ± 12.62 μm; Ang II vs. Ang II + MT: *P* < 0.0001 (Fig. 2D). Consistent with the changes of foetal vessels, trophoblast cells surrounding these vessels were also significantly reduced in PE. To quantify this, trophoblast cells surrounding blood vessels from various placentas were analysed across 12 arbitrary regions from each placenta (as in Fig. 2D). Each region was imaged at 20x, and the trophoblast cells surrounding the blood vessels were counted using Image J. As shown in Fig. 2E, the number of trophoblast cells surrounding blood vessels in the PE group placentas was significantly reduced compared to that in the Sham group (Sham vs. Ang II **, *p* = 0.0035); whereas the number of trophoblast cells in the Sham + MT and Ang II + MT groups was significantly increased compared to that in the Ang II group (Ang II vs. Sham + MT ####, *p* < 0.0001; Ang II vs. Ang II + MT ###, *p* = 0.0001; Fig. 2E).

### Mito-T on placental mitochondria functions

**Fig. 3:**
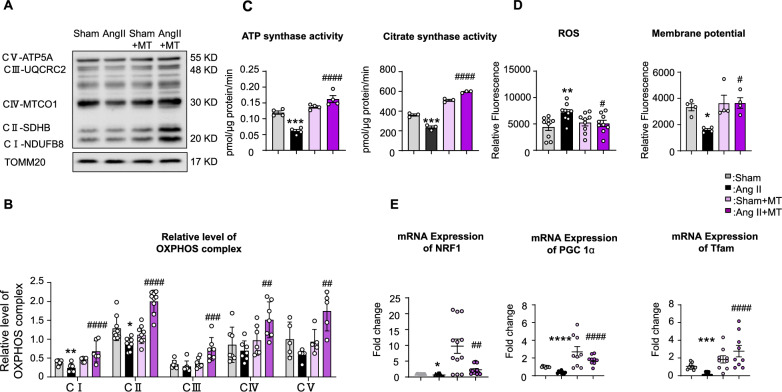
Effects of Mito-T on mitochondrial activity in the placenta. **A** and **B** Immunoblotting results of mitochondrial complex I‒V components in the Sham, Ang II, Sham + MT, and Ang II + MT groups. The results showed significant reductions in CI and CII in Ang II; however, a significant increase was observed in all complex components after Mito-T treatment. (Sham vs. Ang II CI *P* = 0.0267, CII *P* = 0.0035; Ang II *vs.* Ang II + MT CI *P* = 0.0250, CII *P* < 0.0001, CIII *P* = 0.0013, CIV *P* = 0.0005, CV *P* = 0.025, n = 6 for each group). **C** Comparison of ATP and citrate synthase activities to evaluate functional mitochondria in the placentas of the four groups. Results showed that both mitochondrial ATPase and citrate synthase activities were reduced in the Ang II group, and Mito-T significantly increased the activities (ATPase in pmol/ μg protein/min: Sham vs. Ang II: *P* = 0.0002; Ang II vs. Ang II + MT *P* < 0.0001; Citrate synthase in pmol/ μg protein/min: Sham vs. Ang II: *P* = 0.0085; Ang II vs. Ang II + MT *P* < 0.0001) ( ATPase n = 4; Citrate synthase n = 3 for each group). **D** Measurement of mitochondrial ROS and MMP in the placentas of the four groups. The results showed that placental ROS increased and MMP decreased in the Ang II group; however, Mito-T reversed these changes. (ROS: Sham vs. Ang II: *P* = 0.0059; MMP: Sham vs. Ang II: *P* = 0.0059; Ang II vs. Ang II + MT *P* = 0.0217; MMP: Ang II vs. Ang II + MT *P* = 0.001). (ROS & MMP n = 9 for each group). **E** mRNA expression of mitochondrial biogenesis markers (NRF1, PGC-1, TFAM) in the four groups. mRNA of PGC-1α, NRF1, TFAM expression were reduced in Ang II; Mito-T increased the levels of PGC-1α, NRF1, and TFAM (Sham vs. Ang II: PGC-1 α *P* < 0.0001, NRF1 *P* = 0.0131, TFAM *P* < 0.0001; Ang II vs. Sham + MT: PGC-1 α *P* = 0.0484, NRF1 *P* = 0.0005, TFAM *P* = 0.0054; Ang II vs. Ang II + MT: PGC-1 α *P* = 0.0189, NRF1 *P* = 0.0017, TFAM *P* < 0.0001). (NRF1 n = 12; PGC-1α n = 9; TFAM n = 12 for each group). Values are expressed as mean ± SEM. *P < 0.05, **P < 0.005, ****P < 0.0001. * Sham group vs. Ang II group or Sham + MT vs. Ang II + MT; # indicates comparison between the Ang II, Sham + MT, and Ang II + MT groups

The preventive effects of Mito-T suggest an improvement in placental mitochondrial function. First, we examined the protein expression of key components of the electron transport chain (ETC) in the placentas of the four groups. Complex I and II subunits were significantly reduced in the Ang II group (Sham vs. Ang II CI, *P* = 0.0289; CII, *P* = 0.0114); however, Mito-T increased the expression of all complex I‒V components in Ang II-treated rats (Ang II *vs.* Ang II + MT CI, *P* < 0.0001; CII, *P* < 0.0001; CIII, *P* = 0.0008; CIV, *P* = 0.0051; CV, *P* = 0.0013) (Fig. 3A–B).

Both mitochondrial ATPase and citrate synthase activities were reduced in the Ang II group, and Mito-T significantly increased these activities (ATPase activity in pmol/ μg protein/min: Sham *vs.* Ang II: *P* = 0.0003; Ang II *vs.* Ang II + MT *P* < 0.0001; Citrate synthase activity in pmol/ μg protein/min: Sham *vs.* Ang II: *P* = 0.0002; Ang II *vs.* Ang II + MT: *P* < 0.0001) (Fig. 3C). Importantly, placental ROS levels increased and MMP was reduced in Ang II placentas (ROS: Sham *vs.* Ang II: *P* = 0.0059; MMP: Sham *vs.* Ang II: *P* = 0.0416) (Fig. 3D); however, Mito-T treatment reversed both changes (ROS: Ang II *vs.* Ang II + MT, *P* = 0.0217; MMP: Ang II *vs.* Ang II + MT, *P* = 0.0154) (Fig. 3D).

In addition, mRNA levels of mitochondrial biogenesis markers—including peroxisome proliferator-activated receptor gamma coactivator 1 alpha (PGC-1α), nuclear respiratory factor (NRF1), and mitochondrial transcription factor (TFAM)—were reduced in Ang II placentas. Mito-T increased the expression of the three markers (Sham vs. Ang II: PGC-1 α *P* < 0.0001, NRF1 *P* = 0.0131, TFAM *P* < 0.0001; Ang II vs. Ang II + MT: PGC-1 α *P* < 0.0001, NRF1 *P* = 0.0017, TFAM *P* = 0.0002) (Fig. 3E).

### Mito-T on placental mitochondrial dynamics and mitophagy biomarkers

Mito-T may improve mitochondrial function by regulating placental mitochondrial dynamics.

**Fig. 4:**
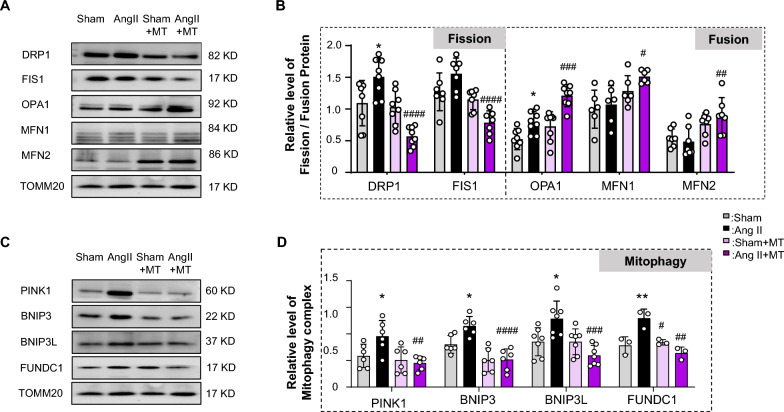
Key proteins of mitochondrial dynamics and mitophagy regulations in PE rat placenta with and without Mito-T. **A** and **B** Immunoblotting results for the mitochondrial fission and fusion proteins DRP1, FIS1, OPA1, MFM1, and MFN2 in the four groups. Mitochondrial fission proteins DRP1 and Fis1 increased in Ang II rats; however, Mito-T reduced the increments (DRP1: Sham vs. Ang II: DRP1 *P* = 0.0285; Ang II vs. Ang II + MT: DRP1 *P* < 0.0001; Ang II vs. Sham + MT: DRP1 *P* = 0.0093; Sham + MT vs. Ang II + MT: DRP1 *P* = 0.0133; Fis 1: Ang II vs. Ang II + MT: Fis 1 *P* = 0.0150; Ang II vs. Sham + MT: Fis 1 *P* < 0.0001; Sham + MT vs. Ang II + MT: Fis 1 *P* = 0.0306). The mitochondrial fusion protein OPA1 was increased in the Ang II rat placenta. After Mito-T treatment, the OPA1 protein level in the Ang II + MT group was significantly increased compared to that in the Ang II and Sham + MT groups. (OPA1: Sham vs. Ang II: OPA1 *P* = 0.0402; Ang II vs. Ang II + MT: OPA1 *P* = 0.0006; Sham + MT vs. Ang II + MT: OPA1 *P* < 0.0001. Mito-fusion proteins (MFN1 and MFN2) showed significantly increased expression in the Ang II + MT group compared to that in the Ang II group after Mito-T treatment. In addition, OPA1, MFN1, and MFN2 protein expression increased, and a significant increase was observed in the Ang II + MT group (Ang II vs. Ang II + MT: OPA1, *P* = 0.0006, MFN1 *P* = 0.0281, MFN2 *P* = 0.0077). (DRP1, n = 8; Fis 1 n = 7; OPA1, n = 8; MFN1, n = 6; MFN 2 n = 7 per group). **C** and **D** Comparison of mitophagy proteins (PINK1, BNIP3, BNIP3L, and FUNDC1) across the four groups. Mitophagy biomarkers—PINK1, BNIP3, BNIP3L, and FUNDC1—were significantly increased in the Ang II group compared to those in the Sham group (Sham vs. Ang II, PINK1 *P* = 0.0495, BNIP3 *P* = 0.0264, BNIP3L *P* = 0.0243, FUNDC1 *P* = 0.0067). Mito-T significantly reduced the expression of these proteins (Ang II vs. Ang II + MT: PINK 1 *P* = 0.0056, BNIP3 *P* < 0.0001, BNIP3L *P* = 0.003, FUNDC1 *P* = 0.0014; Ang II vs. sham + MT: PINK 1 *P* = 0.0143, BNIP3 *P* < 0.0001, BNIP3L *P* = 0.0241, FUNDC1 *P* = 0.013). (PINK1, n = 6; BNIP3, n = 6; BNIP3L, n = 7; FUNDC 1 n = 3 for each group). Values are expressed as **mean ± SEM.** *P < 0.05, **P < 0.005, ****P < 0.0001. * Sham group vs. Ang II group or Sham + MT vs. Ang II + MT; # shows comparison between the Ang II, Sham + MT, and Ang II + MT groups

Representative mitochondrial fission proteins—dynamin-related protein 1 (DRP1) and fission protein 1 (Fis1)—were significantly increased in Ang II-treated rats; however, Mito-T reduced these increases (DRP1: Sham vs. Ang II, *P* = 0.0285; Ang II vs. Ang II + MT, *P* < 0.0001; Fis 1: Ang II vs. Ang II + MT, *P* < 0.0001) (Fig. 4A–B).

Furthermore, mitochondrial fusion protein expressions were detected. As shown in Fig. 4, Optic atrophy 1 (OPA1) was increased in the Ang II rat placenta. After Mito-T treatment, OPA1 protein level in the Ang II + MT group was significantly increased compared to those in the Ang II. (OPA1: Sham vs. Ang II, *P* = 0.0402; Ang II vs. Ang II + MT, *P* = 0.0006).

Mito-fusion protein (MFN1 and MFN2) levels were significantly increased in the Ang II + MT group compared with those in the Ang II group (MFN1: Ang II vs. Ang II + MT, *P* = 0.0281, MFN2: Ang II vs. Ang II + MT, *P* = 0.0077) (Fig. 4A–B).

Mitophagy biomarkers, including PTEN induced putative kinase 1(PINK1), Bcl2/adenovirus E1B 19 kDa protein interacting protein 3 (BNIP3), BNIP3 like (BNIP3L), and Fun14 domain containing 1 (FUNDC 1) were significantly increased in Ang II placentas at GD20 compared to those in Sham (Sham vs. Ang II, PINK 1 *P* = 0.0495, BNIP3 *P* = 0.0264, BNIP3L *P* = 0.0243, FUNDC1 *P* = 0.0067; Fig. 4C–D). Mito-T treatment significantly reduced the levels of these proteins (Ang II vs. Ang II + MT: PINK 1 *P* = 0.0056, BNIP3 *P* < 0.0001, BNIP3L *P* = 0.0003, FUNDC1 *P* = 0.0014) (Fig. 4C–D).

Collectively, these results suggest that Mito-T reduces placental mitochondrial fission and mitophagy biomarkers while enhancing mitochondrial fusion proteins, suggesting the tentative mechanisms of improved mitochondrial functions after Mito-T.

### Signalling pathways regulating sFLT-1 in placental trophoblast cells before and after Mito-T

**Fig. 5:**
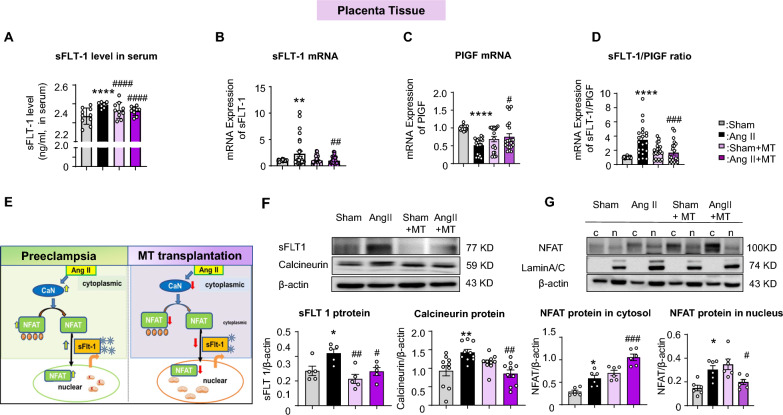
Effects of Mito-T on sFLT-1 and calcineurin/nuclear factor of activated T cells (NFAT) signalling pathways in placental tissue of PE rats. **A** Serum levels of sFlt-1 in the Sham, Ang II, Sham + MT, and Ang II + MT groups. The level of sFlt-1 in the serum of Ang II rats was significantly increased, whereas after Mito-T, it significantly decreased. (n = 10 for each group). **B** sFlt-1 mRNA levels in placental tissues of the four groups. The mRNA expression of sFLT-1 was significantly increased in Ang II and reduced by Mito-T (tissue sFLT-1: Sham: 1.01 ± 0.02, Ang II: 2.04 ± 0.39, Sham + MT: 0.84 ± 0.13, Ang II + MT: 0.83 ± 0.12; Sham vs. Ang II, *P* < 0.0001; Ang II vs. Ang II + MT: *P* < 0.0001). (n = 27 for each group). **C** PlGF mRNA levels in placental tissues of the four groups. PlGF mRNA levels were reduced in the Ang II group; this was reversed by Mito-T (tissue PlGF: Sham: 1.00 ± 0.010, Ang II: 0.66 ± 0.05, Sham + MT: 0.94 ± 0.05, Ang II + MT: 0.85 ± 0.07; Sham vs. Ang II, *P* < 0.0001; Ang II vs. Ang II + MT: *P* < 0.0001). (n = 21 for each group). **D** sFlt-1/PlGF ratio in placental tissues of the four groups. sFlt-1/PlGF ratio was increased in Ang II placenta but attenuated by Mito-T (sFLT-1/PlGF: 1.01 ± 0.02, Ang II: 3.45 ± 1.02, Sham + MT: 1.24 ± 0.16, Ang II + MT: 1.43 ± 0.21; Sham vs. Ang II, *P* < 0.0001; Ang II vs. Ang II + MT: *P* < 0.0001) (n = 18 for each group). **E** Schematic representation of calcineurin and NFAT signalling pathways in the presence and absence of Mito-T in rat PE placentas. **F** sFlt-1, Calcineurin protein expression in placental tissues of the four groups. sFLT 1 protein expression in the placenta tissue of Ang II rats was significantly increased; however, Mito-T reduced this expression (sFLT 1: Sham vs. Ang II, *P* = 0.0229; Ang II vs. Ang II + MT, *P* = 0.0127). Calcineurin protein expression in the placenta tissue of Ang II rats was significantly increased; however, Mito-T reduced this expression (Sham vs. Ang II: calcineurin *P* = 0.0054, Ang II vs. Ang II + MT: calcineurin *P* = 0.0003). (sFLT 1 n = 5; calcineurin n = 10 for each group). **G** NFAT protein expressions in the cytosol and nucleus of placental tissue. NFAT levels of cytoplasm and nucleus in the placenta of Ang II-treated rats were significantly increased (cytoplasmic *P* = 0.0033, nuclear *P* = 0.0493, n = 6 for each group). Mito-T reduced this expression in the nucleus but maintained a high level of NFAT in the cytoplasm (Ang II vs. Ang II + MT: NFAT cytoplasmic *P* = 0.0001, n = 6 for each group). Values are expressed as mean ± SEM. *P < 0.05, **P < 0.005, ****P < 0.0001. * Sham group vs. Ang II group or Sham + MT vs. Ang II + MT; # shows comparison between the Ang II, Sham + MT, and Ang II + MT groups

The increase in sFlt-1 and elevated sFlt-1/placental growth factor (PlGF) ratio are well-established diagnostic biomarkers of PE [6, 8, 9]. Similarly, serum sFlt-1 levels were significantly increased in Ang II-treated rats, whereas Mito-T treatment significantly decreased sFlt-1 levels (Fig. 5A). The mRNA expression of sFLT-1 was significantly increased in the Ang II group and was reduced by Mito-T treatment (tissue sFLT-1: Sham, 1.01 ± 0.02; Ang II, 2.04 ± 0.39; Sham + MT, 0.84 ± 0.13; Ang II + MT, 0.83 ± 0.12; Sham vs. Ang II, *P* = 0.0041; Ang II vs. Ang II + MT: *P* = 0.0031) (Fig. 5B). The expression of PlGF, another important pro-angiogenesis factor, was reduced in the Ang II group; Mito-T reversed this decrease (tissue PlGF: Sham, 1.00 ± 0.010; Ang II, 0.66 ± 0.05; Sham + MT, 0.94 ± 0.05; Ang II + MT, 0.85 ± 0.07; Sham vs. Ang II, *P* < 0.0001; Ang II vs. Ang II + MT: *P* = 0.0237) (Fig. 5C). Consequently, sFlt-1/PlGF ratio, an important placenta biomarker, was increased in Ang II placenta but attenuated by Mito-T (sFLT-1/PlGF: 1.01 ± 0.02, Ang II: 3.45 ± 1.02, Sham + MT: 1.24 ± 0.16, Ang II + MT: 1.43 ± 0.21; Sham vs. Ang II, *P* < 0.0001; Ang II vs. Ang II + MT: *P* = 0.0006) (Fig. 5D).

NFAT, once translocated into the nucleus, binds to the promoter region of Flt-1, activating the gene and promoting the production of an alternative sFlt-1[29–31]. Therefore, we hypothesised that NAFT is dephosphorylated through calcineurin upregulation in Ang II-treated rats, causing NFAT to enter the nucleus and subsequently increasing sFlt-1 transcription and protein levels in the placenta of PE (Fig. 5E). Conversely, Mito-T decreases sFLT-1 production by modulating calcineurin-dependent NFAT signalling (Fig. 5E). Our results demonstrated that sFLT-1 expression in the placenta tissue of Ang II-treated rats was significantly increased (Sham vs. Ang II, *P* = 0.0477), whereas Mito-T reduced this elevation (Ang II vs. Ang II + MT, *P* = 0.0404) (Fig. 5F). Similarly, calcineurin protein expression in the placenta tissue of Ang II rats was significantly increased and was reduced by Mito-T (Sham vs. Ang II: calcineurin *P* = 0.0055, Ang II vs. Ang II + MT: Calcineurin *P* = 0.0012) (Fig. 5F).

Placental tissue was further separated into cytoplasmic and nuclear components, and the results showed that both cytoplasmic and nuclear NFAT protein levels were significantly increased in the placenta of Ang II-treated rats (cytoplasmic *P* = 0.0124, nuclear *P* = 0.0131) (Fig. 5G). Mito-T reduced NFAT expression in the nucleus but maintained a high level of NFAT in the cytoplasm (Ang II vs. Ang II + MT, cytoplasmic *P* = 0.0001, nuclear *P* = 0.0266) (Fig. 5G).

**Fig. 6:**
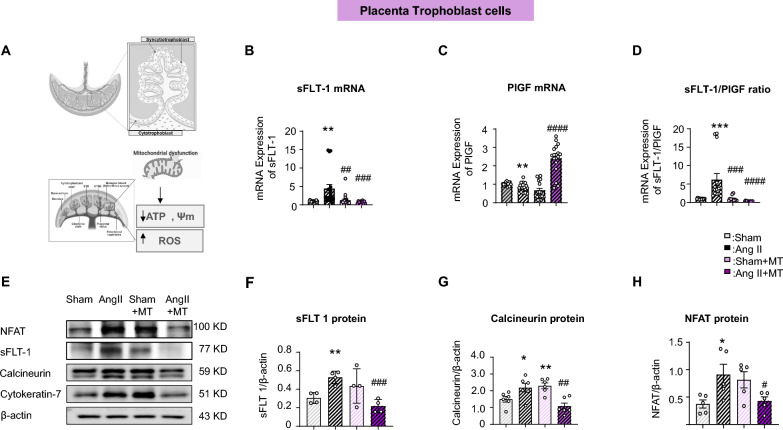
Effects of Mito-T on sFLT-1 and calcineurin/nuclear factor of activated T cells (NFAT) signalling pathways in placental trophoblast cells. **A** Schematic diagram of the effect of placental trophoblast on placental mitochondria. **B** sFlt-1 mRNA levels in isolated placental trophoblasts from the four groups. sFLT-1 mRNA expression was greater in the Ang II group than in the sham group; Mito-T reduced sFLT-1 level (sFLT-1: Sham: 1.01 ± 0.14, Ang II: 4.41 ± 4.87, *p* = 0.0055; Sham + MT: 1.24 ± 1.63, Ang II + MT: 0.67 ± 0.37; Ang II vs. Ang II + MT: *P* = 0.0026). (n = 18 for each group). **C** PlGF mRNA levels in isolated placental trophoblasts from the four groups. PlGF mRNA was reduced in Ang II-treated rats, and Mito-T increased it (Sham: 1.00 ± 0.09, Ang II: 0.82 ± 0.24, *P* = 0.0040; Sham + MT: 0.67 ± 0.48, Ang II + MT: 0.21 ± 0.80; Ang II vs. Ang II + MT: *P* < 0.0001) (n = 18 for each group). **D** sFlt-1/PlGF ratio in isolated placental trophoblasts from the four groups. sFlt-1/PlGF ratio was significantly high in Ang II-treated rats, and Mito-T reduced it (Sham: 1.01 ± 0.15, Ang II: 5.49 ± 6.04, Sham vs. Ang II, *P* = 0.0034; Sham + MT: 3.40 ± 5.62, Ang II + MT: 0.29 ± 0.14; Ang II vs. Ang II + MT: *P* = 0.0009) (n = 18 for each group). **E** Comparison of sFlt-1, NFAT, and calcineurin proteins across the four groups of isolated placental trophoblasts (Western blot bands). **F** sFlt-1 protein expression in isolated placental trophoblasts from the four groups. sFlt-1 was increased in Ang II-treated rats, whereas Mito-T attenuated such increases, consistent with those obtained from placental tissue. (Sham vs. Ang II, *P* = 0.0025; Ang II vs. Ang II + MT: *P* = 0.0007) (n = 4 for each group). **G**, **H** Calcineurin and NFAT protein expression in placental trophoblasts isolated from the four groups. The protein expression of calcineurin and NFAT was increased in Ang II-treated rats and reduced by Mito-T (calcineurin: *P* = 0.0496, NFAT: *P* = 0.0299 between Sham and Ang II; calcineurin *P* = 0.0063, NFAT *P* = 0.0007 between Ang II and Ang II + MT). (Calcineurin, n = 6; NFAT, n = 5 per group). Values are expressed as mean ± SEM. *P < 0.05, **P < 0.005, ****P < 0.0001. * Sham group vs. Ang II group or Sham + MT vs. Ang II + MT; # shows comparison between the Ang II, Sham + MT, and Ang II + MT groups

We isolated and purified trophoblasts from the placenta to validate our earlier findings regarding the Ang II–induced increase in sFLT-1 and the sFLT-1/PlGF ratio. The results showed that sFLT-1 mRNA expression was greater in Ang II-treated trophoblasts than in the Sham group (sFLT-1: Sham, 1.01 ± 0.14; Ang II, 4.41 ± 4.87; Sham vs. Ang II, *P* = 0.0010) (Fig. 6B); Mito-T reduced sFLT-1 level (Sham + MT: 1.24 ± 1.63, Ang II + MT: 0.67 ± 0.37; Ang II vs. Ang II + MT: *P* = 0.0003) (Fig. 6B). In addition, PlGF mRNA levels were reduced in the Ang II group (Sham: 1.00 ± 0.09, Ang II: 0.82 ± 0.24, Sham vs. Ang II, *P* = 0.0040)(Fig. 6C), whereas Mito-T increased PlGF expression (Sham + MT: 0.67 ± 0.48, Ang II + MT: 0.21 ± 0.80; Ang II vs. Ang II + MT: *P* < 0.0001) (Fig. 6C). Consequently, the sFlt-1/PlGF ratio was significantly elevated in the Ang II group (Sham: 1.01 ± 0.15, Ang II: 5.49 ± 6.04, Sham vs. Ang II, *P* = 0.003) (Fig. 6D), and Mito-T reduced it (Sham + MT: 3.40 ± 5.62, Ang II + MT: 0.29 ± 0.14; Ang II vs. Ang II + MT: *P* < 0.0001) (Fig. 6D).

In placental trophoblasts, the protein expression of calcineurin and NFAT was increased in the Ang II group, and both were reduced by Mito-T (calcineurin: *P* = 0.0496, NFAT: *P* = 0.0299 between Sham and Ang II; calcineurin: *P* = 0.0063, NFAT: *P* = 0.0007 between Ang II and Ang II + MT) (Fig. 6 G-H). Similarly, sFlt-1 was increased in Ang II trophoblasts (*P* = 0.0025) (Fig. 6F), and Mito-T attenuated this increase (*P* = 0.0007) (Fig. 6F), consistent with the results obtained from whole placental tissue.

### Mito-T affects placental mitochondrial dynamics in Bewo cells to improve mitochondrial function.

Among the four commonly used trophoblast cell lines—BeWo, Jar, Jeg-3, and HTR-8/SVneo—a study by Zhou et al. [32] reported that although BeWo, Jar, and Jeg-3 showed significantly enhanced sFlt-1 mRNA expression in response to hypoxia, only BeWo secreted detectable sFlt-1 protein [33]. Therefore, to investigate the effects of Mito-T on the placental function of Ang II-induced PE rats, we used the common human trophoblast cell line BeWo for in vitro experiments.

**Fig. 7:**
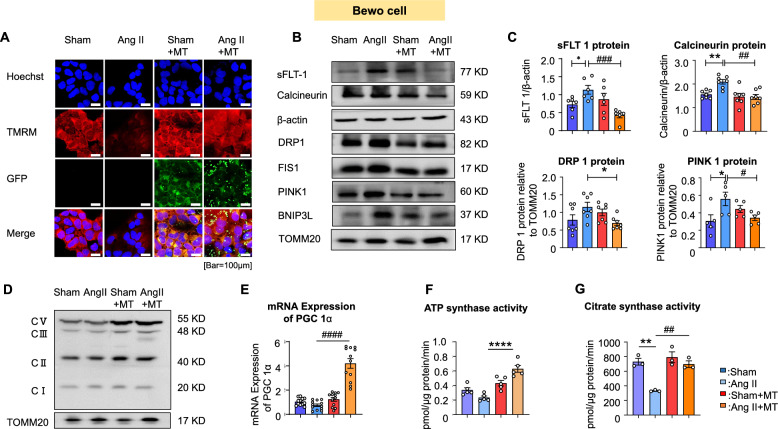
Effects of Mito-T on Ang II-induced sFLT-1 regulation and its mechanisms in BeWo cells. **A** Confocal microscopy showed that injected mitochondria isolated from hUC-MSC clearly entered the Bewo cells. TMRM results showed that MMP, which was reduced in the Ang II group, was increased with Mito-T. **B** and **C** Immunoblotting analysis of sFLT-1 and calcineurin proteins in BeWo cells, and of DRP1, Fis1, PINK1, and BNIP3L proteins in the mitochondria of BeWo cells across the four groups. The sFLT-1results were consistent with those of the placental tissue and placental trophoblast cells. Calcineurin protein in the Ang II group was increased significantly (calcineurin: *p* = 0.007, Sham vs. Ang II). After Mito-T treatment, calcineurin protein levels were significantly reduced compared to those in the Ang II group (calcineurin: *p* = 0.0012, Ang II vs. Ang II + MT). (sFLT-1, n = 6; calcineurin, n = 7 per group). DRP1 protein levels increased in the Ang II group, and Mito-T reduced this increase (DRP1: Ang II vs. Ang II + MT, *P* = 0.0423). The mitophagy biomarker PINK1 in BeWo cells was significantly increased in the Ang II group (sham vs. Ang II, PINK1, *P* = 0.0334). (DRP1, n = 7; PINK 1 n = 5 per group). **D** Comparison of mitochondrial complex components in the four groups of BeWo cells. **E** PGC-1α mRNA levels in the four groups of Bewo cells. The mRNA expression of the mitochondrial biogenesis marker PGC-1α was decreased in Ang II BeWo cells; however, Mito-T significantly increased the level of PGC-1α (Ang II vs. Ang II + MT, *P* < 0.0001) (n = 12 per group). **F** and **G** Comparison of ATP and citrate synthase activities in the four groups of BeWo cells. The mitochondrial ATPase and citrate synthase activities were decreased in Bewo cells in the Ang II group, which was reversed by Mito-T (ATP in pmol/μg protein/min: Ang II vs. Ang II + MT *P* < 0.0001 Fig. 7F; Citrate in pmol/μg protein/min: Sham vs. Ang II: *P* = 0.0020; Ang II vs. Ang II + MT *P* = 0.0034 Fig. 7G) (ATP n = 6; Citrate n = 3 for each group). Values are expressed as mean ± SEM. *P < 0.05, **P < 0.005, ****P < 0.0001. * Sham group vs. Ang II group or Sham + MT vs. Ang II + MT; # shows comparison between the Ang II, Sham + MT, and Ang II + MT groups

First, we used confocal microscopy to determine whether the transplanted mitochondria entered BeWo cells. The results showed that green GFP was visible in the Sham + MT and Ang II + MT groups, but not in the Sham and Ang II groups, indicating that the transplanted mitochondria had indeed entered BeWo cells (Fig. 7A).

TMRM (50 nM) staining was used to compare MMP across the four groups (△Ψm). The results showed that the TMRM fluorescence signal was significantly reduced in Bewo cells treated with Ang II, indicating impaired MMP, whereas Mito-T treatment significantly enhanced the fluorescence signal, demonstrating restoration of MMP (TMRM, red, Fig. 7A).

Subsequently, we examined the expression of sFlt-1 and calcineurin proteins in BeWo cells. The results were consistent with those obtained from placental tissue and isolated trophoblast cells (Fig. 7B). As illustrated in Fig. 7C, calcineurin protein levels were significantly increased in the Ang II group compared to Sham (calcineurin: *P* = 0.0071, sham vs. Ang II), and sFLT-1 protein levels also to increase(sFLT-1:*P* = 0.0211,) (Fig. 7C). After Mito-T treatment, calcineurin protein levels were significantly reduced compared to those in the Ang II group (calcineurin: *P* = 0.0019, Ang II vs. Ang II + MT) (Fig. 7C). Similarly, the expression of sFlt-1 protein was significantly reduced after Mito-T treatment (sFlt-1: *P* = 0.0019, Ang II vs. Ang II + MT) (Fig. 7C).

sFlt-1 consists of multiple splice variants, with sFLT-1 i13 and sFLT-1 e15a being translated into human proteins [33]. We measured the mRNA expression of these two variants in BeWo cells. As shown in Data Supplement 3A & B, sFLT-1 i13 and sFLT-1 e15a levels were increased in the Ang II group and reduced by Mito-T (sFlt-1 i13: *P* < 0.0001, Sham vs. Ang II; *P* < 0.0001, Ang II vs. Ang + MT; sFlt-1 e15a: *P* = 0.0013, Sham vs. Ang II; *P* = 0.0007, Ang II vs. Ang II + MT).

Subsequently, DRP1 and Fis 1 proteins were increased in the Ang II group, and Mito-T reduced these increases (DRP1: Ang II vs. Ang II + MT, *P* = 0.0423; Fis1: Sham vs. Ang II, *P* = 0.0258; Ang II vs. Ang II + MT, *P* < 0.0001) (Fig. 7B–C and Data supplement 3C).

The mitophagy biomarkers PINK1 and BNIP3L in BeWo cells were significantly increased in the Ang II group (Sham vs. Ang II, PINK1 *P* = 0.0334, BNIP3L *P* = 0.0017) (Fig. 7B-C, Data supplement 3D). Mito-T also significantly reduced the expression of these proteins (Ang II vs. Ang II + MT: PINK 1 *P* = 0.0320, BNIP3L *P* = 0.0255) (Fig. 7B–C, Fig. SE). The mRNA expression of the mitochondrial biogenesis marker PGC-1α was decreased in Ang II BeWo cells but increased after Mito-T treatment (Ang II vs. Ang II + MT, *P* < 0.0001) (Fig. 7E). In addition, Mito-T increased the protein expression of the complex V component of the electron transport chain in BeWo cells. (Fig. 7D).

Mitochondrial enzymatic activities were also affected: ATPase and citrate synthase activities were decreased in Ang II-treated Bewo cells, and these reductions were reversed by Mito-T (ATPase activity in pmol/μg protein/min: Ang II vs. Ang II + MT *P* < 0.0001 Fig. 7F; Citrate synthase activity in pmol/μg protein/min: Sham vs. Ang II: *P* = 0.0020; Ang II vs. Ang II + MT *P* = 0.0034 Fig. 7G).

## Discussion

**Fig. 8:**
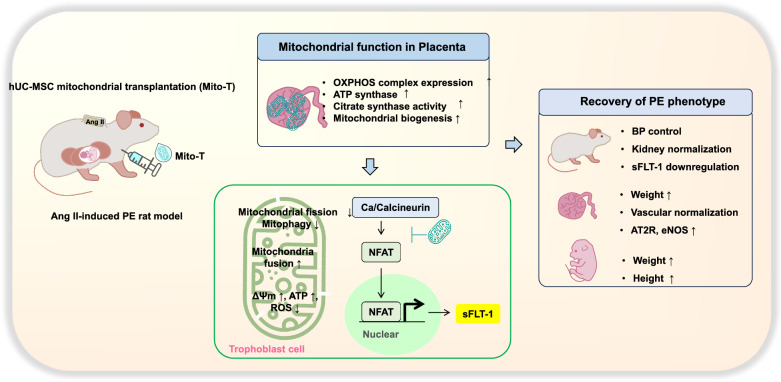
Schematic diagram showing the effects and mechanisms of hUC-MSC-derived Mito–T in a PE rat model. Maternal phenotypes can be improved *by* increasing placental mitochondrial function. sFLT-1 secretion from trophoblast cells was reduced by calcineurin-NFAT-dependent signalling in an Ang II-induced rat PE model

This study demonstrates that midterm administration of hUC-MSC-derived mitochondria in PE can protect against maternal and foetal abnormalities. Experimental findings support the following: (1) Mito-T controlled high BP in PE rats and reversed glomerular structural dysregulation; (2) Mito-T increased foetal weight and crown rump length, which were reduced in PE; (3) sFLT-1 mRNA and protein levels were increased in maternal sera, placental tissue, and trophoblast cells; Mito-T reduced sFLT-1 levels and the sFLT-1/PlGF ratio, indicating normalisation in the placenta. Immunohistochemistry demonstrated disease phenotypes in placental structure and foetal vessels of PE rats, which Mito-T restored to normal level; (4) Mito-T improved placental mitochondria functions (ATP citrate synthase activities, ROS reduction, MMP, biogenesis marker mRNA increment) and enhanced key elements of mitochondrial ETC complexes; (5) Mito-T administration shifted mitochondria toward a fusion state by reducing mitochondrial fission or mitophagy proteins and increasing fusion proteins, indicative of improved mitochondrial stability; (6) In placental tissues, isolated trophoblast cells, and cultured BeWo cell lines, sFLT-1, calcineurin, and nuclear NFAT 1 proteins were increased in PE rats; Mito-T reduced the increment of these proteins. These findings provide, *for the first time*, evidence of the advantageous effects of hUC-MSC-derived mitochondria in PE (Fig. 8).

Placental malperfusion affects maternal and foetal metabolism through mitochondrial dysregulation, including the reduction of key components of the ETC and increases in oxidative stress due to diminished oxidative capacity, consequently attenuating mitochondrial biogenesis. Our results clearly showed that Mito-T administration improved mitochondrial ETC functions (increased MMP and reduced ROS levels) and biogenesis (increased mRNA expression of NRF, Tfam, and PGC 1α). Protein levels of complexes I‒V were increased, suggesting mitochondrial DNA upregulation and post-translational functional regulation. Accordingly, Mito-T reversed the dysregulation of mitochondrial dynamics and mitophagy in PE; specifically, Mito-T administration reduced mitochondrial fission and mitophagy to normal levels and increased mitochondrial fusion in placental trophoblast cells.

Recovery of mitochondrial function was attributed to the intravenous administration of Mito‑T, which facilitated its translocation into target tissues. However, the precise mechanism by which Mito‑T traverses from the bloodstream into tissues remains unclear. Since isolated mitochondria are vulnerable to calcium‑induced depolarization of the membrane potential [34], the chelating agent EGTA was added during the isolation process to mitigate this risk. Although exposure to circulating calcium during intravascular delivery poses a potential threat to mitochondrial integrity, Mito‑T may reach target tissues before irreversible damage occurs. Supporting this hypothesis, previous studies have shown that in ischemic hearts, transplanted mitochondria localize in the myocardium within 1 h after administration, increasing tissue ATP content and exerting cardioprotective effects [35]. Similarly, in cultured keratinocytes, exogenous mitochondria were internalized within 20 min and subsequently integrated into the host mitochondrial network, restoring both structural integrity and bioenergetic function [36]. Moreover, cell‑free mitochondria have been detected circulating in the blood, indicating that mitochondria can remain relatively stable even under physiological calcium concentrations [37]. Collectively, these findings suggest that intravenously delivered mitochondria are not necessarily degraded by calcium exposure but can remain functional and actively translocate to tissues to confer therapeutic benefits. Consistent with these observations, this study demonstrated increased mitochondrial protein abundance and enhanced mitochondrial function following administration, indicating that exposure to blood calcium does not compromise the therapeutic efficacy of transplanted mitochondria.

Mitochondria in the bloodstream cross the endothelial barrier and accumulate in organs through several mechanisms involving cellular uptake and transcellular transport. One major pathway is endocytosis or macropinocytosis by endothelial cells [30, 38], which allows mitochondria to be internalized from the bloodstream into target tissues and cells. After internalization, mitochondria can pass through the endothelial cytoplasm and be released at the abluminal (tissue-facing) surface, facilitating their transport to underlying organ tissues. Mitochondrial transport via tunnelling nanotubes or extracellular vesicles has also been reported in some biological contexts [39]. Furthermore, inflammation or tissue damage can temporarily increase endothelial permeability [40], facilitating mitochondrial transport. Overall, mitochondrial transmigration across the endothelial barrier is a complex, multifactorial process involving cellular uptake, vesicle transport, and changes in endothelial permeability under both physiological and pathological conditions. However, it remains unclear whether mitochondria are transported through cellular membranes intact and remain structural and functional integrity in the cells. Further research is essential to determine detailed mechanisms of mitochondria transportation from venous injection to target cellular functions in the placenta. Addressing this knowledge gap is critical to fully understand the physiological significance and therapeutic potential of Mito-T in cardiovascular diseases.

The fate of mitochondria that migrate into host tissues is consistent with that of endogenous mitochondria. Transplanted mitochondria, either injected locally, administered intravascularly, or incubated in culture media, are taken up within minutes into various cell types of target organs through endocytosis [38]. Specific labelling of transplanted mitochondria indicates that the majority (~ 80%) integrate with the local mitochondrial network, resulting in increased ATP synthesis and higher cellular ATP content [38]. In the current PE model, we observed that labelled hUC-MSC mitochondrial proteins were expressed in the kidney and placenta from GD15 to GD17 and GD20. The observed increase in mitochondrial fusion in PE following Mito-T administration suggests that transplanted hUC-MSC mitochondria are maintained through fusion processes in these tissue cells; alternatively, transplanted mitochondrial components could integrate with endogenous mitochondria, contributing to mitochondrial DNA replacement and subsequent translation and protein processes [38–40]. This is essential for mitochondrial quality control, which is impaired in PE but appears to be restored with Mito-T during recovery.

This study focused on the biodistribution and therapeutic efficacy of Mito-T, as the kidney, uterus, and placenta are the primary organs affected in PE [41, 42]. Although other organs were not evaluated in this study, previous reports have demonstrated that intravenously administered mitochondria preferentially migrate to sites of cellular injury [26], suggesting targeted delivery to the kidney, uterus, and placenta. Notably, no toxicity or adverse effects were observed in control animals after Mito-T treatment, allowing for a focused assessment of therapeutic efficacy in PE.

The stability of placental trophoblast cells is a critical prerequisite for sFLT-1 reduction and disease recovery in PE. Biochemical analysis indicated that calcineurin-NFAT-dependent signalling is upregulated in PE, contributing to increased sFLT-1 levels [31], whereas Mito-T administration reversed these pathways. Similar findings were observed in isolated trophoblasts and a human trophoblast cell line (BeWo cells). Mito-T treatment (directly or indirectly) improved mitochondria function across these study samples—potentially through integration of transplanted mitochondria with endogenous mitochondria from trophoblast cells—leading to enhanced energy status, reduced ischaemic stress, and attenuated inflammation. These improvements likely suppressed cellular stress signals, such as the calcineurin/NFAT pathway, thereby reducing sFLT-1 production. Although the mechanisms by which human-derived mitochondria regulate trophoblast sFLT-1 production require further investigation, these results strongly support that hUC-MSC mitochondria are promising targets for PE therapy drug development.

Accumulating evidence has shown that Mito-T consistently exerts beneficial effects in various ischaemic diseases, including PE [32–38]. These effects can be attributed to systemic and local regulation. For example, immune responses to cross-species mitochondria can exacerbate inflammation and the consequent cardiovascular events. However, Mito-T cells are not associated with autoimmune or inflammatory reactions [38–40]. Recent experimental studies using hUC-MSC mitochondria in LPS-induced sepsis mice demonstrated attenuation of systemic inflammation [42, 43]. Hypertension or PE is associated with oxidative stress and inflammation [44, 45], which affects systemic cardiovascular function, particularly kidney dysfunction [46–50]. The observation that Mito-T restored kidney structure, foetal weight, and crown-rump length at GD20, along with the restoration of foetal vasculature, strongly supports the safety of Mito-T use without causing additional complications that may hamper foetal delivery and development.

We believe that Mito-T derived from hUC-MSCs could potentially be used in human PE. Compelling evidence shows that injected hUC-MSC mitochondria are distributed in systemic organs for up to 7 days post-transplantation, with mitochondrial activity upregulated and maintained throughout the experimental period in our study and others [25]. Mitochondria transplantation has demonstrated therapeutic efficacy in models of isolated heart injury [51], wound healing [52], and inflammatory diseases [24, 25, 53] by restoring mitochondrial function. Mito-T has been evaluated for safety through toxicology and dose-ranging studies, and its therapeutic efficacy in inflammatory myopathies has been confirmed in clinical trials [25]. These findings suggest that Mito-T can be a quick and effective regimen for treating PE. As an emerging therapeutic strategy, stem cell-derived mitochondria improve mitochondrial function in placental trophoblast cells, regulate sFLT-1 release, and exert short- or long-term beneficial effects through multiple mechanisms, such as antioxidant and anti-inflammatory effects.

## Conclusion

Mito T is a novel therapeutic strategy for PE, with hUC-MSC-derived mitochondria providing an “easy to apply” and efficient potential treatment. Our study, using animal models of PE, provides proof-of-concept for the application of Mito T in this disease. In humans, systematic and thorough investigations of the dose and timing of Mito T administration are required to define a precise blueprint for PE therapy.

## Supplementary Information

Additional file 1 (PPTX 31892 KB)

Additional file 2 (PPTX 4863 KB)

Additional file 3 (DOCX 19 KB)

## Data Availability

All data are included in the manuscript or supporting materials.

## Abbreviations

Ang II: Angiotensin II
Mito-T: Mitochondrial transplantation
sFlt-1: Soluble fms-like tyrosine kinase 1
PlGF: Placental growth factor
PE: Preeclampsia
GD: Gestational day
SD: Sprague-Dawley
BP: Blood pressure
ROS: Reactive oxygen species
hUC-MSC: Human umbilical cord mesenchymal stem cells
NFAT: Nuclear factor of activated T-cell
MMP: Mitochondrial membrane potential
H&E: Haematoxylin/eosin
PGC-1α: Peroxisome proliferator-activated receptor gamma coactivator 1-alpha
NRF1: Nuclear respiratory factor 1
Tfam: Mitochondrial transcription factor A
OPA1: Optic atrophy 1
DRP1: Dynamin-related protein 1
FIS1: Fission 1
MFN: Mitofusin
PINK1: PTEN-induced putative kinase 1
BNIP3: Bcl2/adenovirus E1B 19 kDa protein-interacting protein 3
BNIP3L: BNIP3-like
FUNDC1: Fun14 domain containing 1

## Declarations

### Author statement

No works generated by artificial intelligence were used in this article.
