# Supplementary material for: Human umbilical mesenchymal stem cell-derived mitochondria transplantation suppresses sFLT-1 secretion by regulating calcineurin-NFAT-dependent pathways in angiotensin II-induced preeclampsia rats
Source: Stem Cell Res Ther. 2026 Feb 13;17:92. doi: 10.1186/s13287-026-04930-9 (PMC12954920; doi:10.1186/s13287-026-04930-9)
Supplement: Supplementary file 1 — Additional file 1 (PPTX 31892 KB) [file 13287_2026_4930_MOESM1_ESM.pptx]

## Slide 1
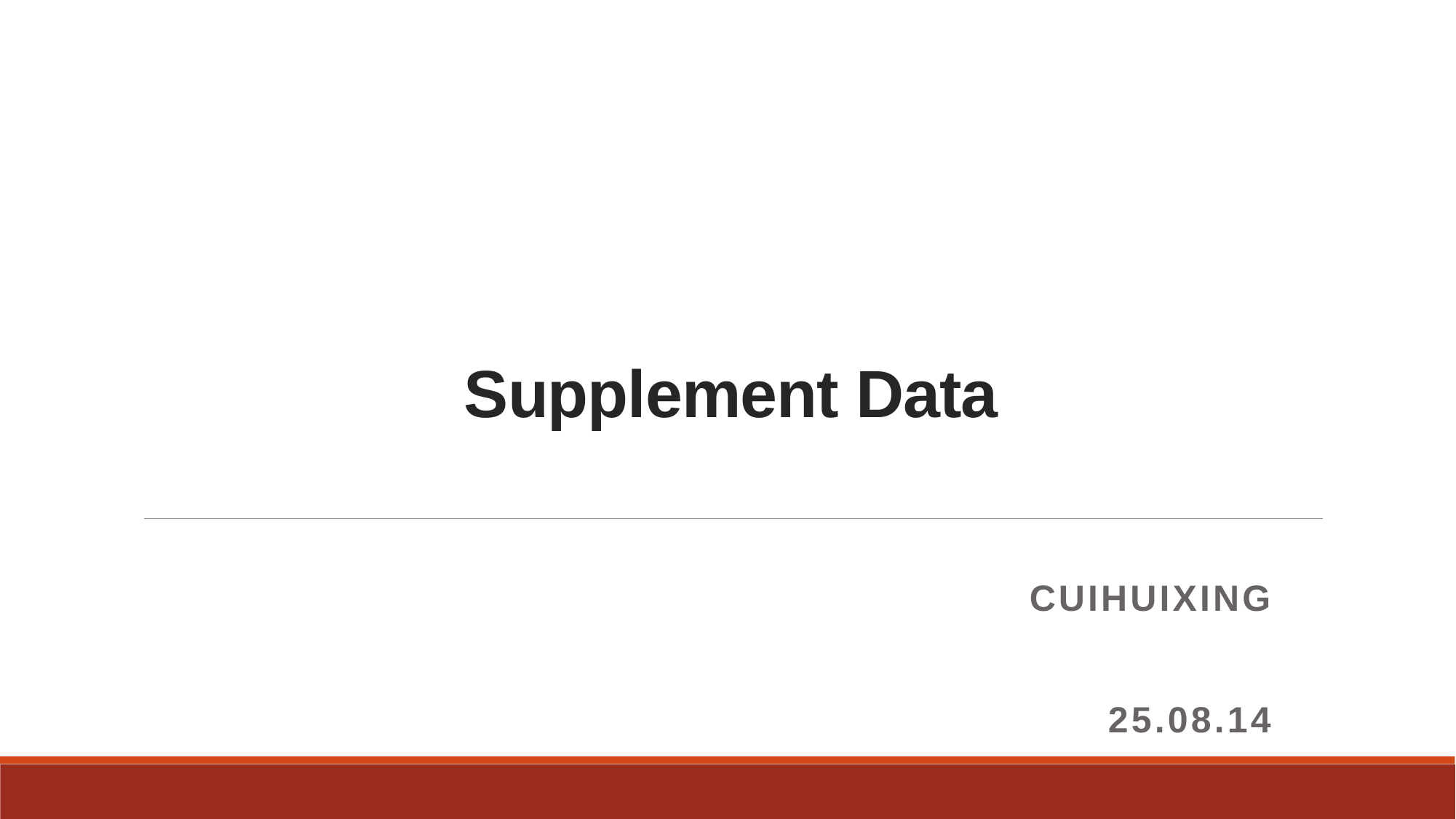

# Supplement Data
CUIHUIXING
25.08.14

## Slide 2
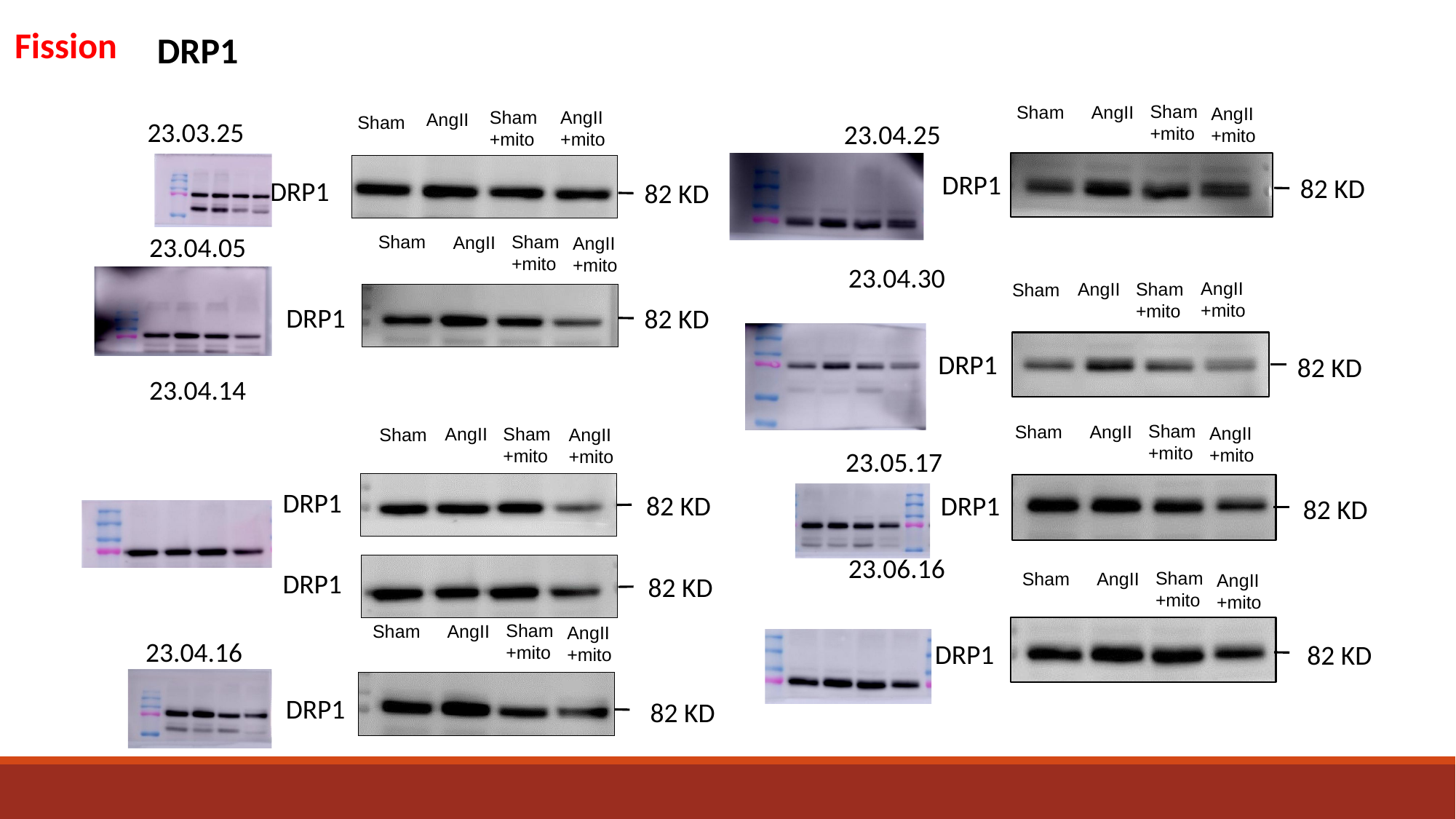

Fission
DRP1
Sham
+mito
Sham
AngII
AngII
+mito
Sham
+mito
AngII
+mito
AngII
Sham
23.03.25
23.04.25
DRP1
82 KD
DRP1
82 KD
23.04.05
Sham
+mito
Sham
AngII
AngII
+mito
23.04.30
AngII
AngII
+mito
Sham
+mito
Sham
DRP1
82 KD
DRP1
82 KD
23.04.14
Sham
+mito
Sham
AngII
AngII
+mito
AngII
Sham
+mito
Sham
AngII
+mito
23.05.17
DRP1
82 KD
DRP1
82 KD
23.06.16
DRP1
Sham
+mito
Sham
AngII
AngII
+mito
82 KD
Sham
+mito
Sham
AngII
AngII
+mito
23.04.16
DRP1
82 KD
DRP1
82 KD

## Slide 3
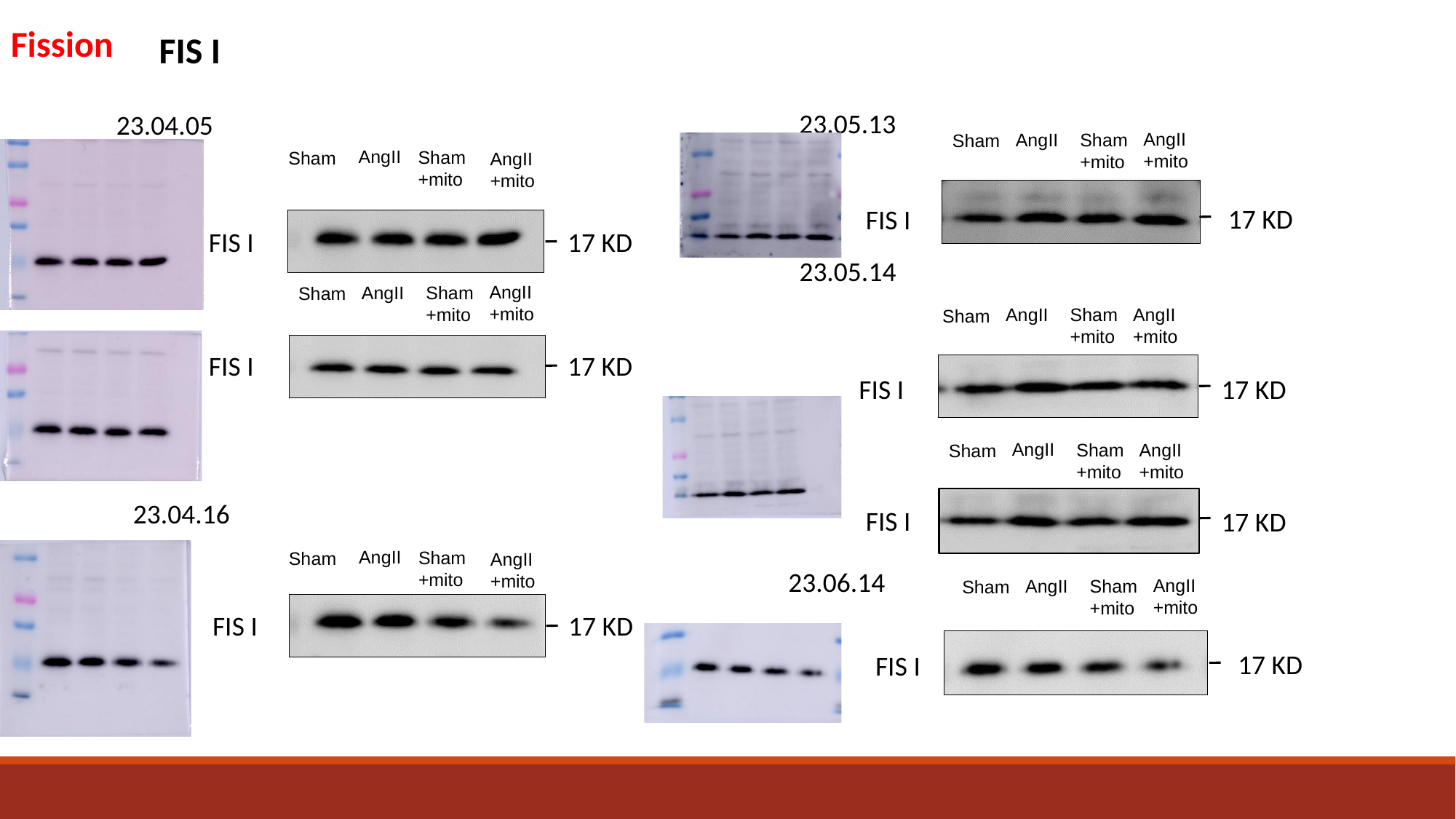

Fission
FIS I
23.05.13
23.04.05
AngII
+mito
AngII
Sham
+mito
Sham
AngII
Sham
+mito
Sham
AngII
+mito
17 KD
FIS I
FIS I
17 KD
23.05.14
AngII
+mito
AngII
Sham
+mito
Sham
AngII
Sham
+mito
AngII
+mito
Sham
FIS I
17 KD
FIS I
17 KD
AngII
Sham
+mito
AngII
+mito
Sham
23.04.16
FIS I
17 KD
AngII
Sham
+mito
Sham
AngII
+mito
23.06.14
AngII
+mito
AngII
Sham
+mito
Sham
17 KD
FIS I
17 KD
FIS I

## Slide 4
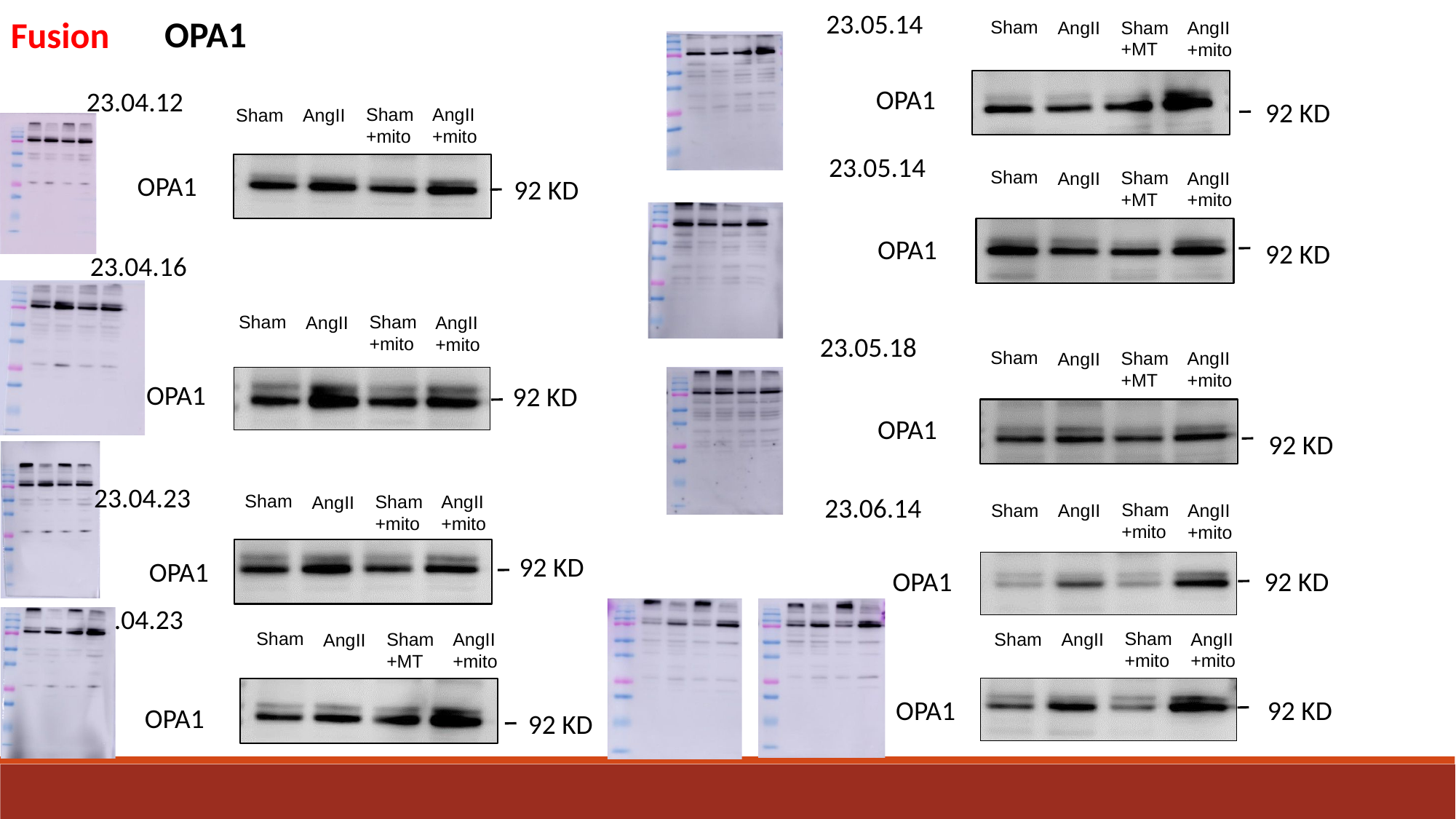

23.05.14
OPA1
Fusion
Sham
Sham
+MT
AngII
+mito
AngII
OPA1
23.04.12
92 KD
Sham
+mito
AngII
+mito
AngII
Sham
23.05.14
Sham
Sham
+MT
AngII
+mito
AngII
OPA1
92 KD
OPA1
92 KD
23.04.16
Sham
Sham
+mito
AngII
+mito
AngII
23.05.18
Sham
Sham
+MT
AngII
+mito
AngII
OPA1
92 KD
OPA1
92 KD
23.04.23
Sham
Sham
+mito
AngII
+mito
23.06.14
AngII
Sham
+mito
AngII
+mito
AngII
Sham
92 KD
OPA1
OPA1
92 KD
23.04.23
Sham
+mito
Sham
AngII
+mito
Sham
+MT
AngII
Sham
AngII
+mito
AngII
OPA1
92 KD
OPA1
92 KD

## Slide 5
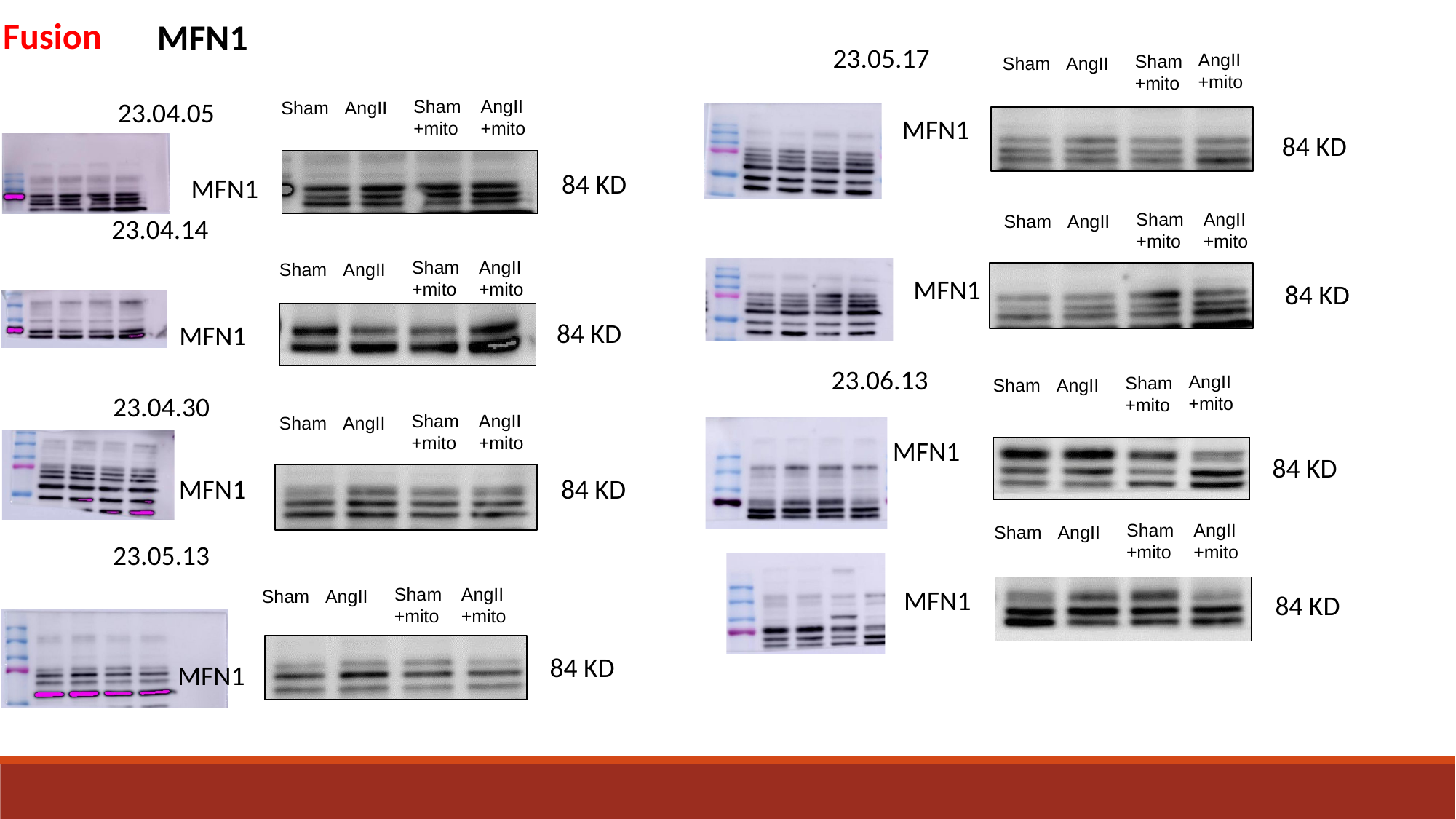

Fusion
MFN1
23.05.17
AngII
+mito
Sham
+mito
AngII
Sham
AngII
+mito
Sham
+mito
23.04.05
AngII
Sham
MFN1
84 KD
84 KD
MFN1
AngII
+mito
Sham
+mito
AngII
Sham
23.04.14
AngII
+mito
Sham
+mito
AngII
Sham
MFN1
84 KD
84 KD
MFN1
23.06.13
AngII
+mito
Sham
+mito
AngII
Sham
23.04.30
AngII
+mito
Sham
+mito
AngII
Sham
MFN1
84 KD
MFN1
84 KD
AngII
+mito
Sham
+mito
AngII
Sham
23.05.13
AngII
+mito
Sham
+mito
MFN1
AngII
Sham
84 KD
84 KD
MFN1

## Slide 6
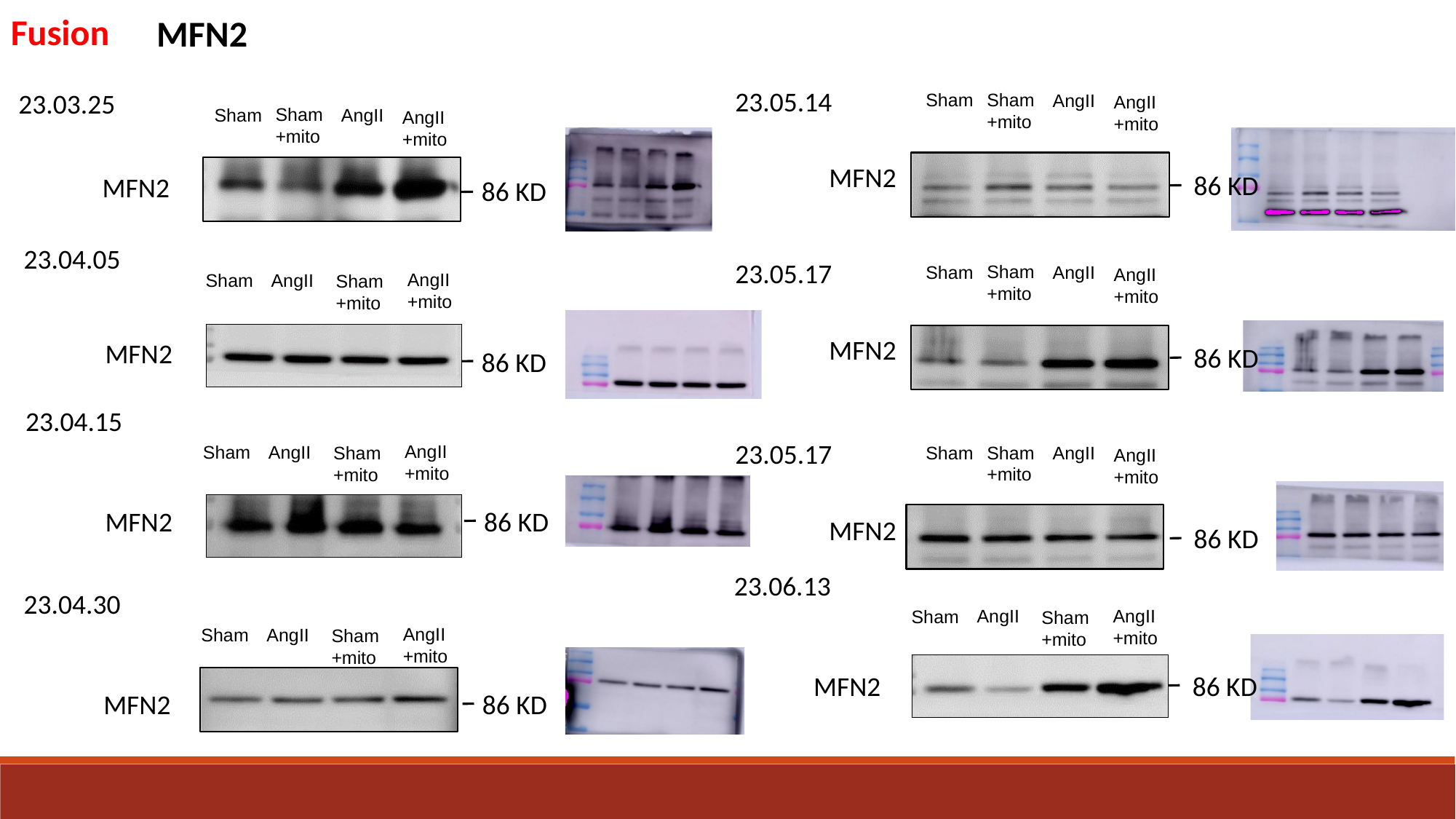

Fusion
MFN2
23.05.14
23.03.25
Sham
+mito
Sham
AngII
AngII
+mito
Sham
+mito
Sham
AngII
AngII
+mito
MFN2
86 KD
MFN2
86 KD
23.04.05
23.05.17
Sham
+mito
Sham
AngII
AngII
+mito
AngII
+mito
AngII
Sham
Sham
+mito
MFN2
MFN2
86 KD
86 KD
23.04.15
23.05.17
AngII
+mito
AngII
Sham
Sham
+mito
Sham
Sham
+mito
AngII
AngII
+mito
MFN2
86 KD
MFN2
86 KD
23.06.13
23.04.30
AngII
+mito
AngII
Sham
Sham
+mito
AngII
+mito
AngII
Sham
Sham
+mito
MFN2
86 KD
MFN2
86 KD

## Slide 7
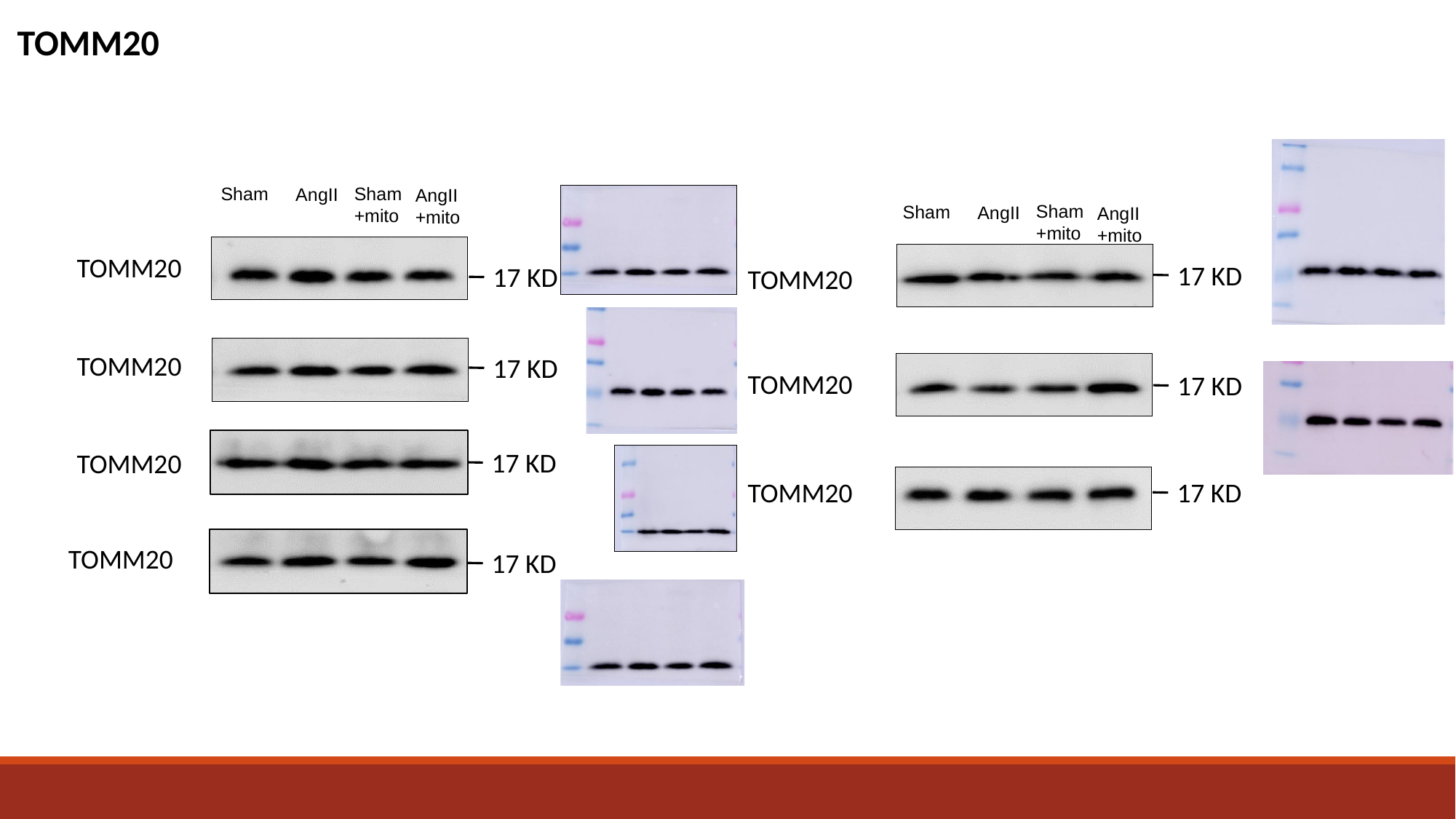

TOMM20
Sham
+mito
Sham
AngII
AngII
+mito
Sham
+mito
Sham
AngII
AngII
+mito
TOMM20
17 KD
17 KD
TOMM20
TOMM20
17 KD
TOMM20
17 KD
17 KD
TOMM20
TOMM20
17 KD
TOMM20
17 KD

## Slide 8
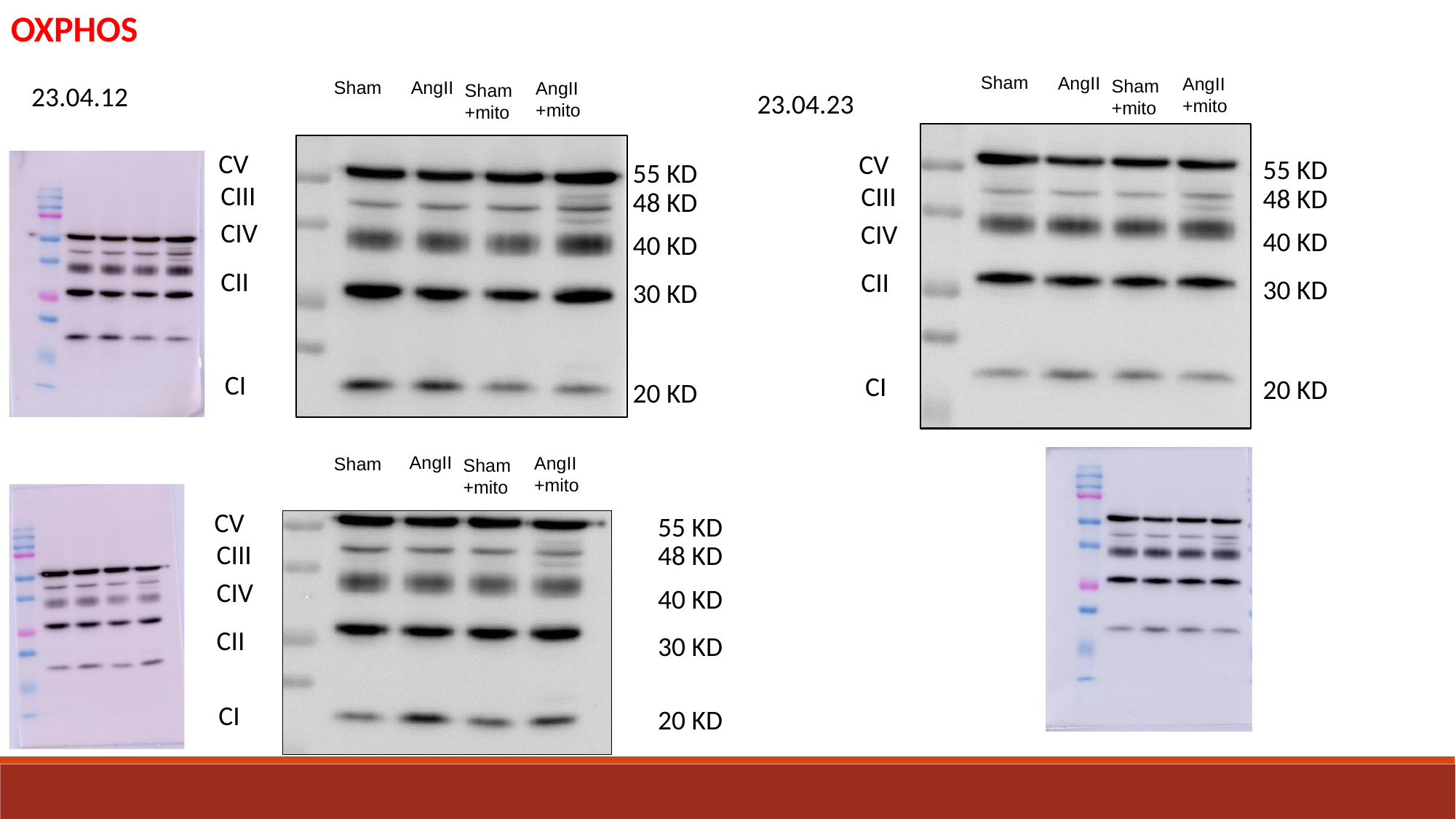

OXPHOS
Sham
AngII
AngII
+mito
Sham
+mito
Sham
AngII
AngII
+mito
23.04.12
Sham
+mito
23.04.23
CV
CV
55 KD
55 KD
CIII
CIII
48 KD
48 KD
CIV
CIV
40 KD
40 KD
CII
CII
30 KD
30 KD
CI
CI
20 KD
20 KD
AngII
AngII
+mito
Sham
Sham
+mito
CV
55 KD
CIII
48 KD
CIV
40 KD
CII
30 KD
CI
20 KD

## Slide 9
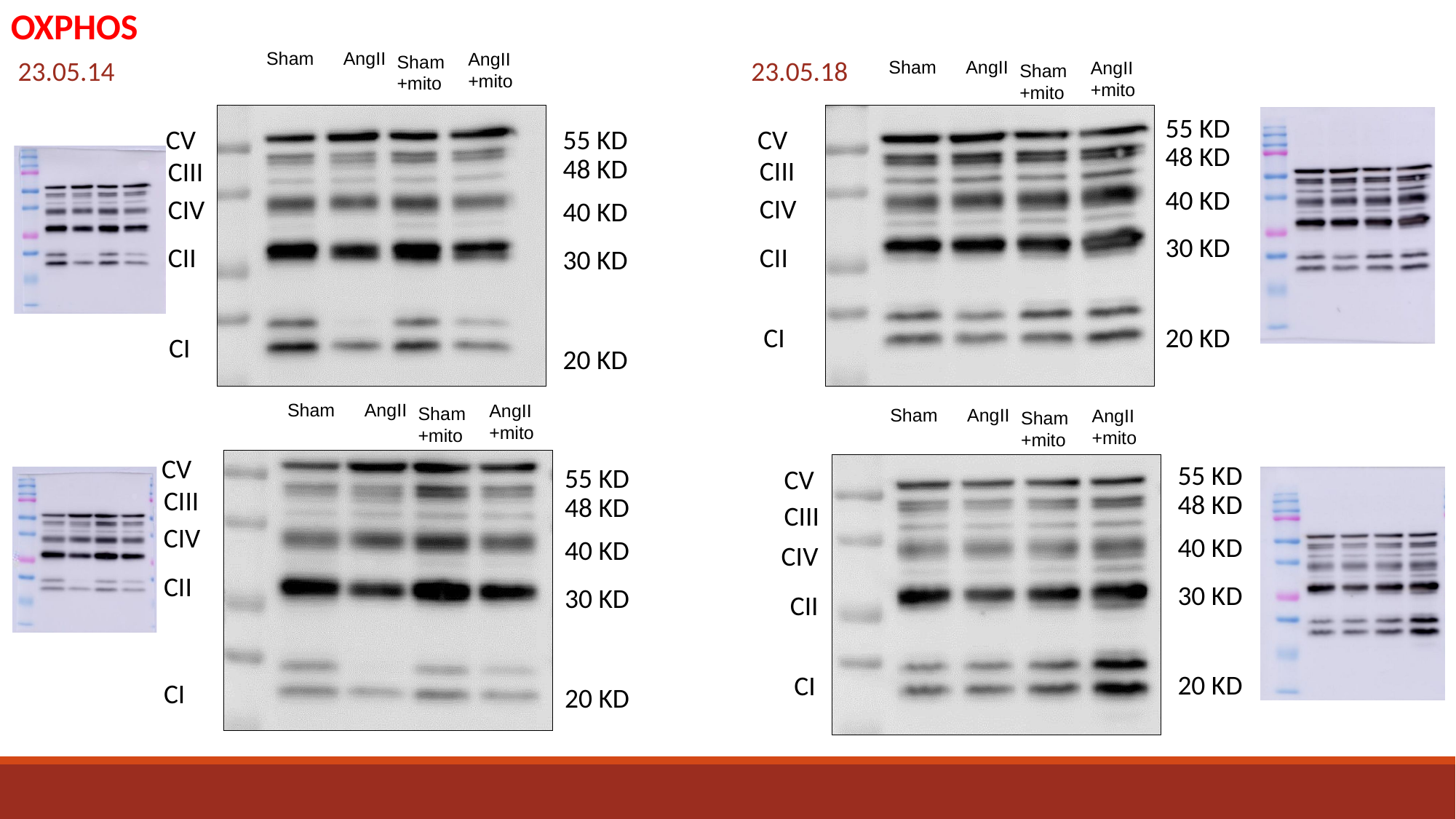

OXPHOS
Sham
AngII
AngII
+mito
Sham
+mito
23.05.14
23.05.18
Sham
AngII
AngII
+mito
Sham
+mito
55 KD
CV
CV
55 KD
48 KD
48 KD
CIII
CIII
40 KD
CIV
CIV
40 KD
30 KD
CII
CII
30 KD
CI
20 KD
CI
20 KD
Sham
AngII
AngII
+mito
Sham
+mito
Sham
AngII
AngII
+mito
Sham
+mito
CV
55 KD
55 KD
CV
CIII
48 KD
48 KD
CIII
CIV
40 KD
40 KD
CIV
CII
30 KD
30 KD
CII
20 KD
CI
CI
20 KD

## Slide 10
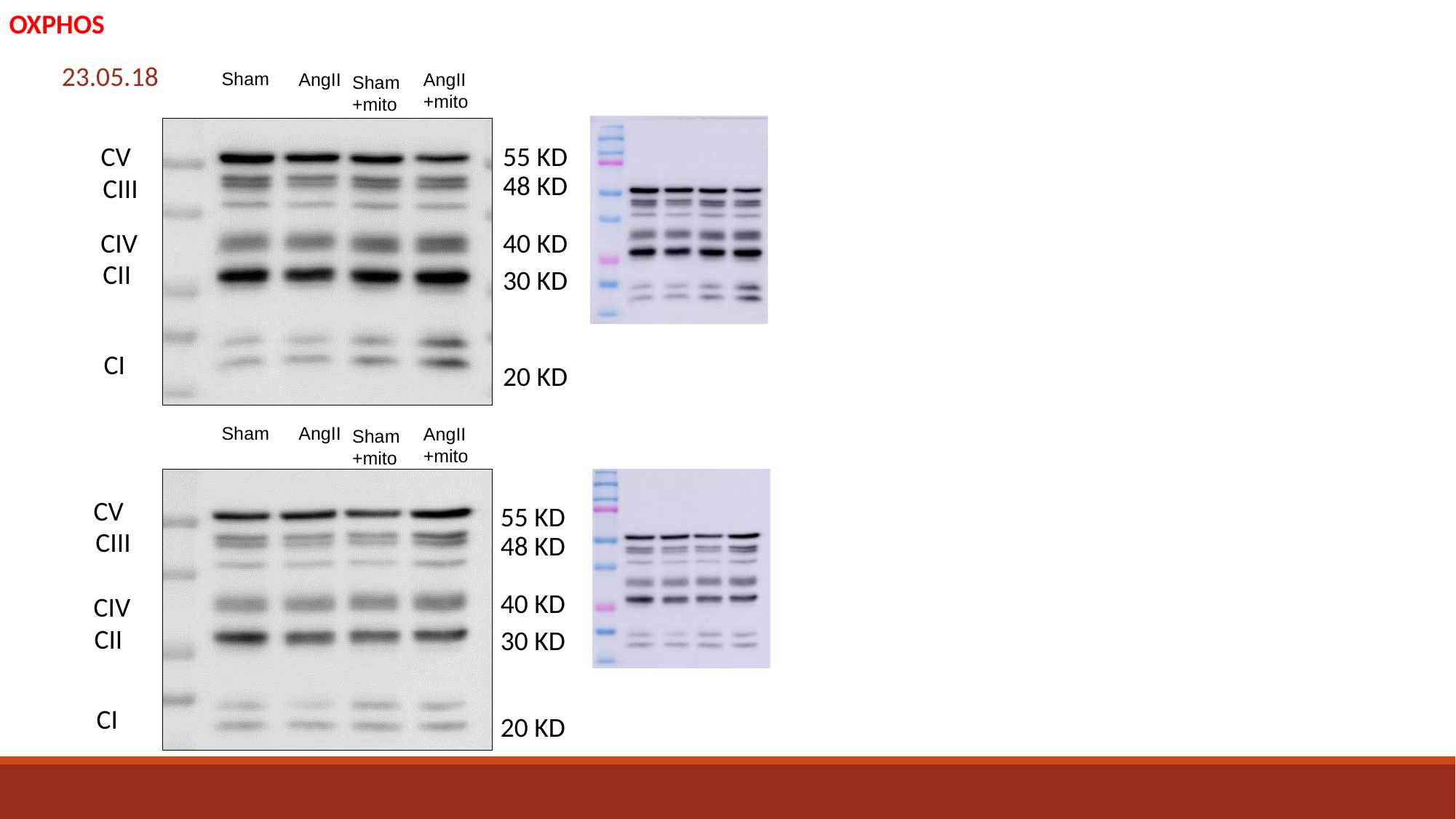

OXPHOS
23.05.18
Sham
AngII
AngII
+mito
Sham
+mito
CV
55 KD
48 KD
CIII
CIV
40 KD
CII
30 KD
CI
20 KD
Sham
AngII
AngII
+mito
Sham
+mito
CV
55 KD
CIII
48 KD
40 KD
CIV
CII
30 KD
CI
20 KD

## Slide 11
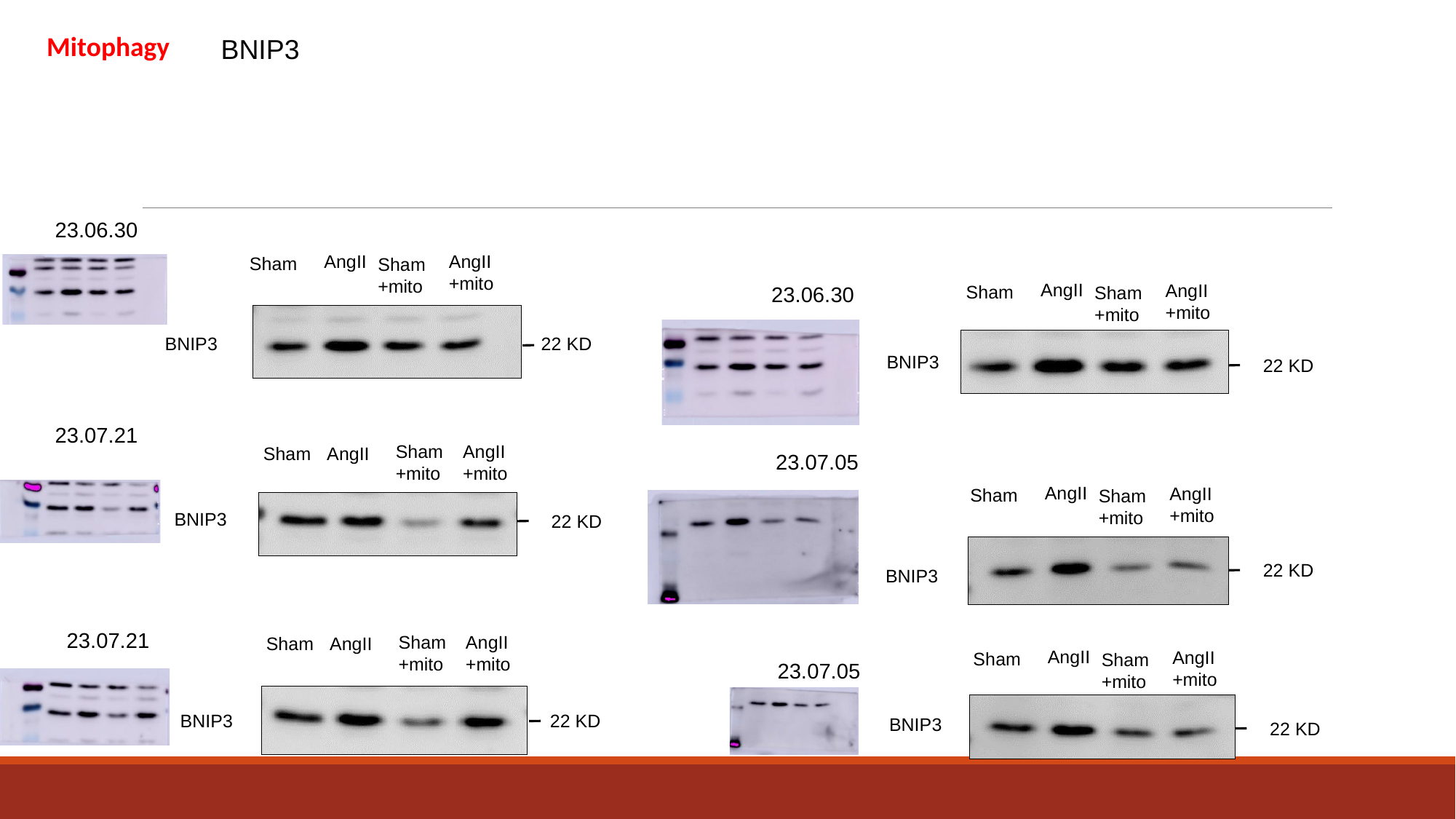

Mitophagy
BNIP3
23.06.30
AngII
AngII
+mito
Sham
Sham
+mito
AngII
AngII
+mito
Sham
23.06.30
Sham
+mito
BNIP3
22 KD
BNIP3
22 KD
23.07.21
AngII
+mito
Sham
+mito
AngII
Sham
23.07.05
AngII
AngII
+mito
Sham
Sham
+mito
BNIP3
22 KD
22 KD
BNIP3
23.07.21
AngII
+mito
Sham
+mito
AngII
Sham
AngII
AngII
+mito
Sham
Sham
+mito
23.07.05
BNIP3
22 KD
BNIP3
22 KD

## Slide 12
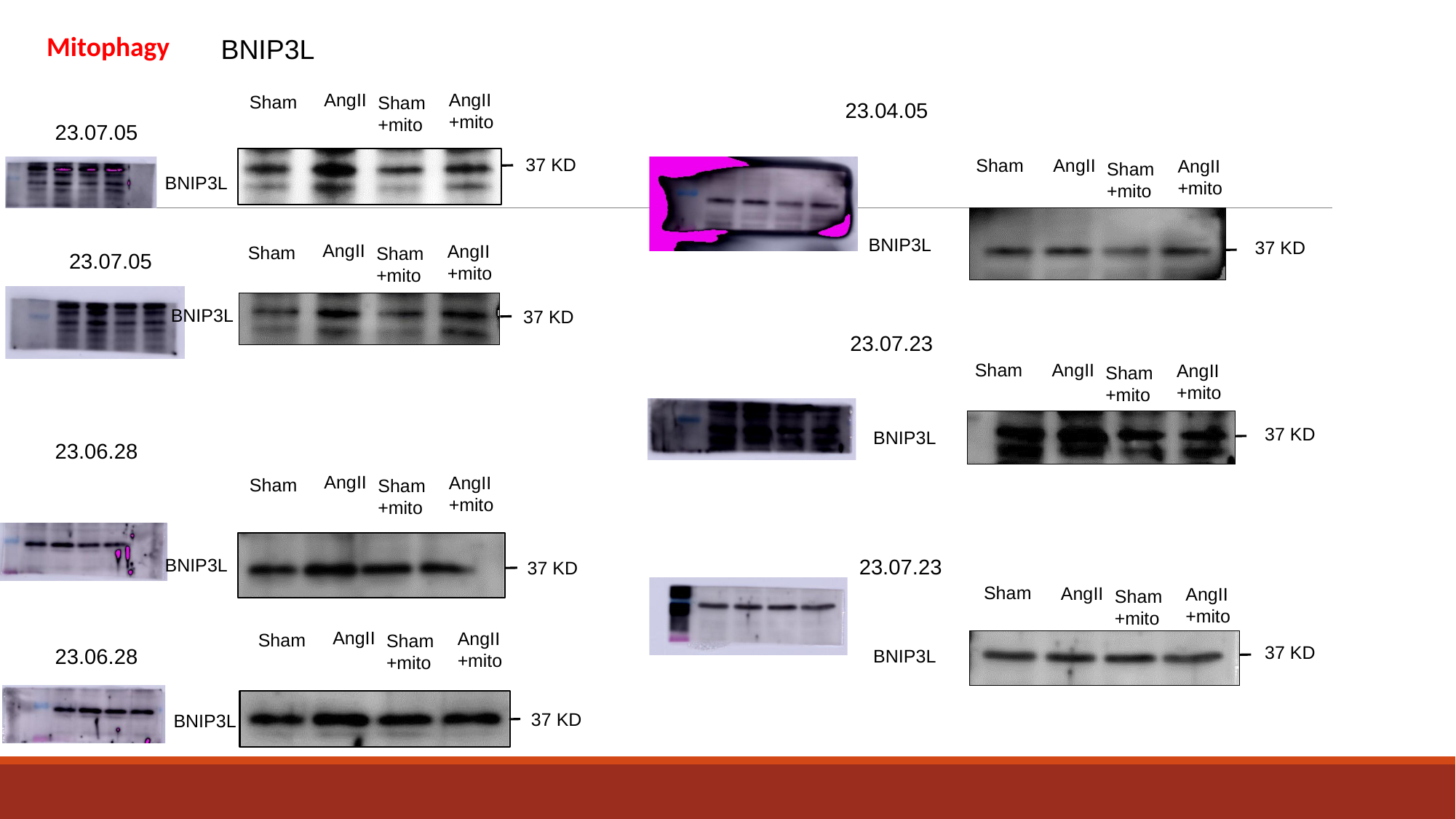

Mitophagy
BNIP3L
AngII
AngII
+mito
Sham
Sham
+mito
23.04.05
23.07.05
37 KD
Sham
AngII
AngII
+mito
Sham
+mito
BNIP3L
BNIP3L
37 KD
AngII
AngII
+mito
Sham
Sham
+mito
23.07.05
BNIP3L
37 KD
23.07.23
Sham
AngII
AngII
+mito
Sham
+mito
37 KD
BNIP3L
23.06.28
AngII
AngII
+mito
Sham
Sham
+mito
23.07.23
BNIP3L
37 KD
Sham
AngII
AngII
+mito
Sham
+mito
AngII
AngII
+mito
Sham
Sham
+mito
37 KD
23.06.28
BNIP3L
37 KD
BNIP3L

## Slide 13
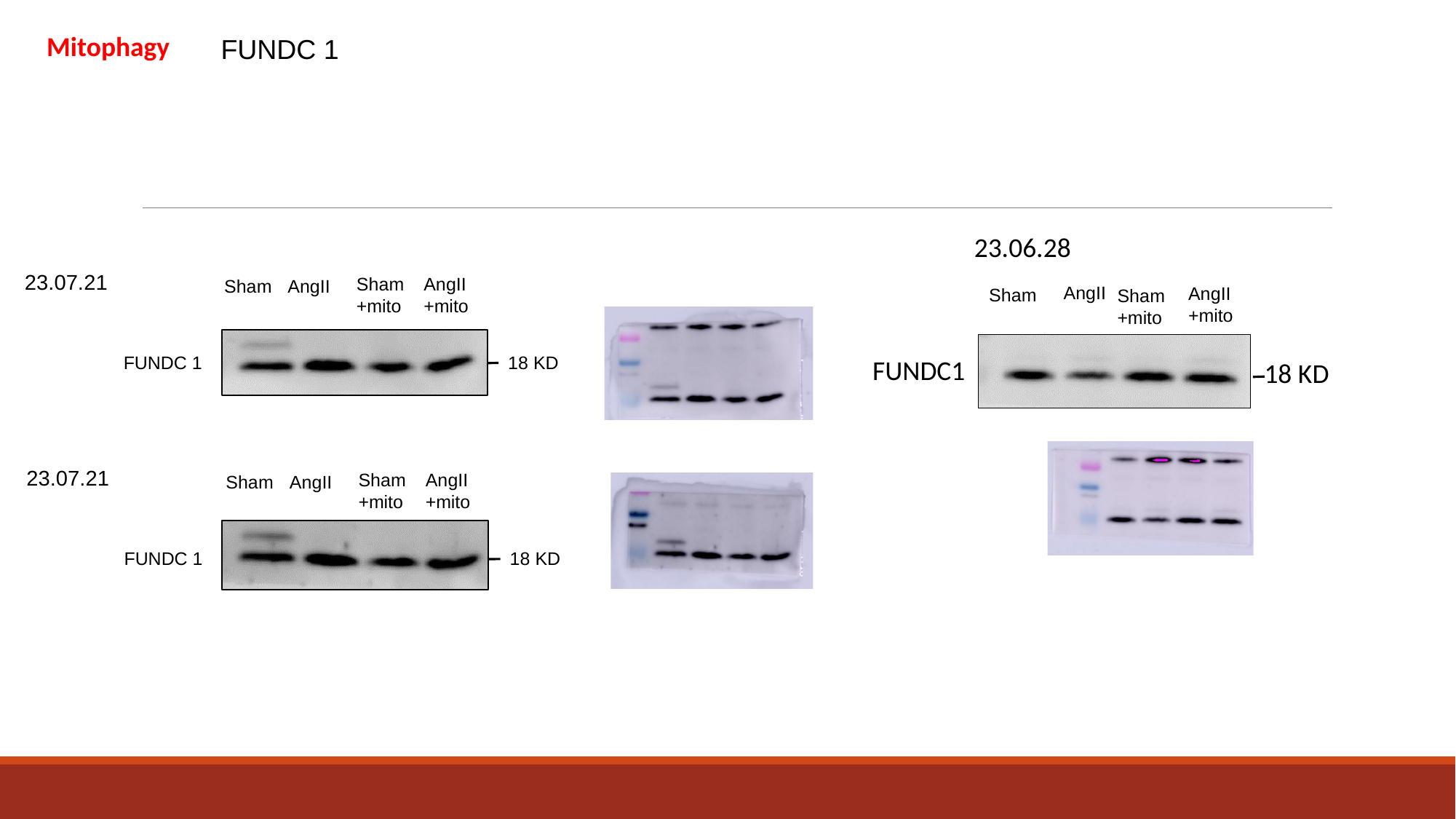

Mitophagy
FUNDC 1
23.06.28
23.07.21
AngII
+mito
Sham
+mito
AngII
Sham
AngII
AngII
+mito
Sham
Sham
+mito
FUNDC 1
18 KD
FUNDC1
18 KD
23.07.21
AngII
+mito
Sham
+mito
AngII
Sham
FUNDC 1
18 KD

## Slide 14
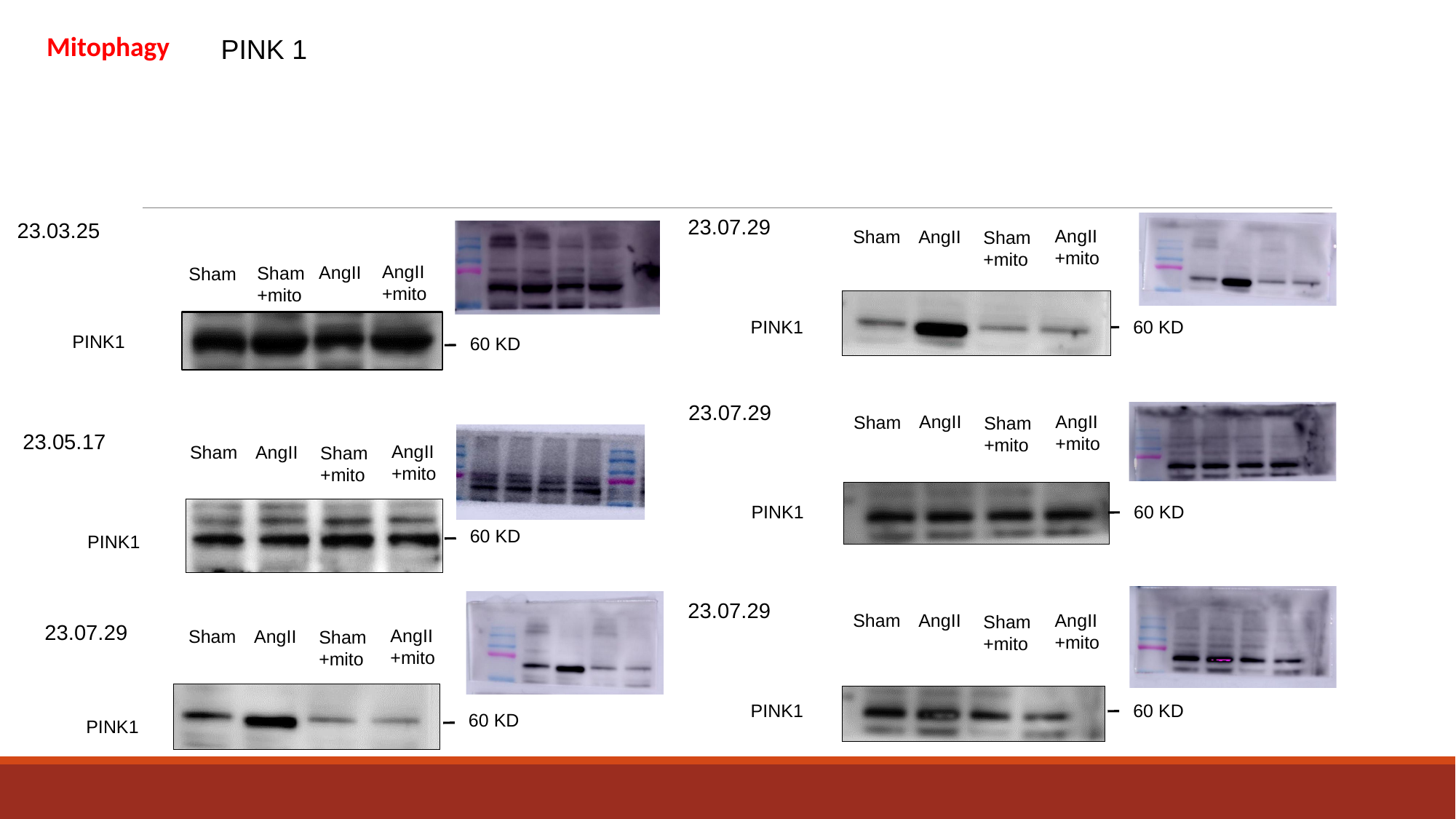

Mitophagy
PINK 1
23.07.29
23.03.25
AngII
+mito
AngII
Sham
Sham
+mito
AngII
+mito
AngII
Sham
+mito
Sham
60 KD
PINK1
PINK1
60 KD
23.07.29
AngII
+mito
AngII
Sham
Sham
+mito
23.05.17
AngII
+mito
AngII
Sham
Sham
+mito
60 KD
PINK1
60 KD
PINK1
23.07.29
AngII
+mito
AngII
Sham
Sham
+mito
23.07.29
AngII
+mito
AngII
Sham
Sham
+mito
60 KD
PINK1
60 KD
PINK1

## Slide 15
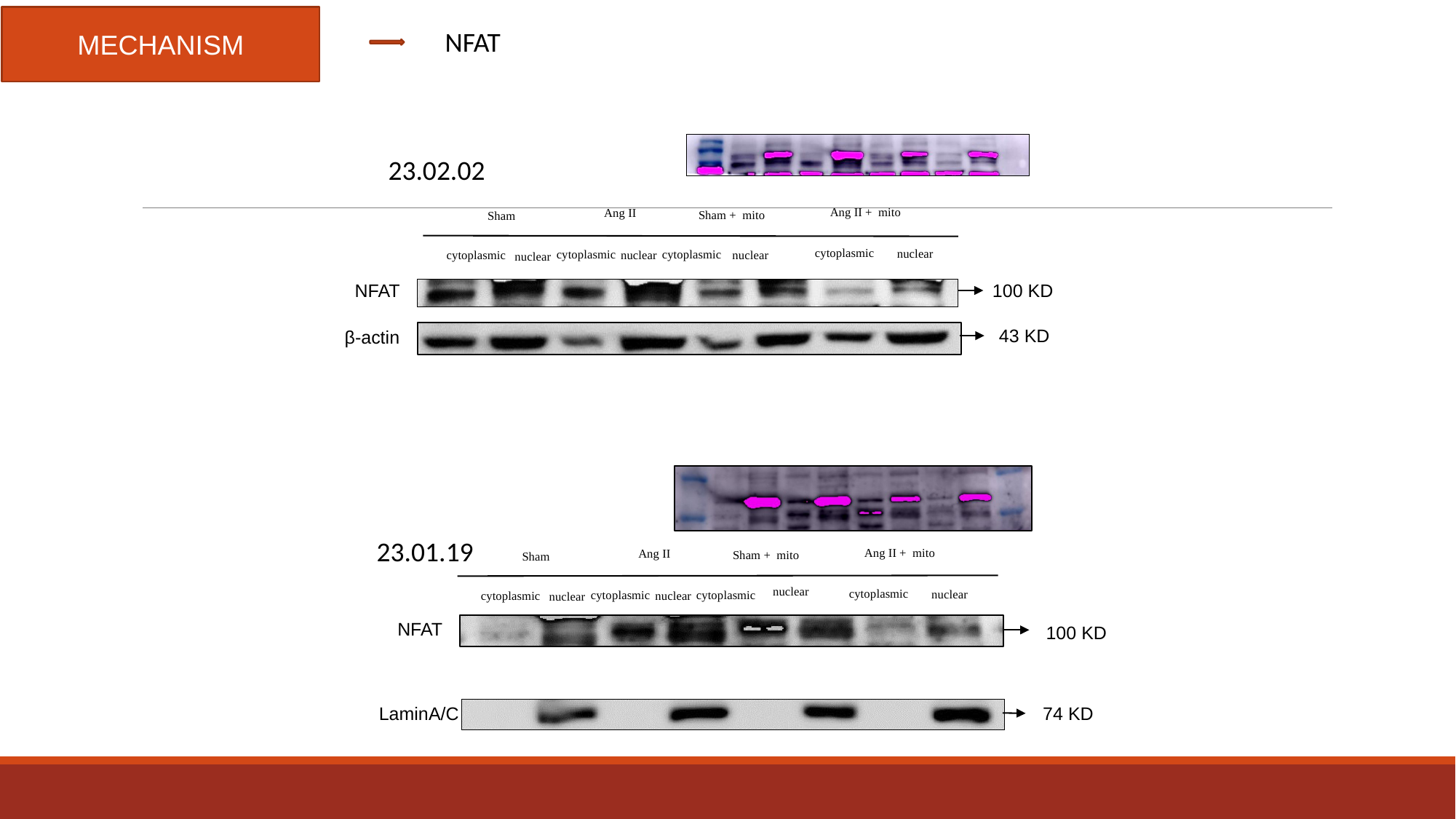

MECHANISM
NFAT
#
23.02.02
Ang II + mito
Ang II
Sham + mito
Sham
cytoplasmic
nuclear
cytoplasmic
cytoplasmic
cytoplasmic
nuclear
nuclear
nuclear
100 KD
NFAT
43 KD
β-actin
23.01.19
Ang II + mito
Ang II
Sham + mito
Sham
nuclear
cytoplasmic
nuclear
cytoplasmic
cytoplasmic
cytoplasmic
nuclear
nuclear
NFAT
100 KD
74 KD
LaminA/C

## Slide 16
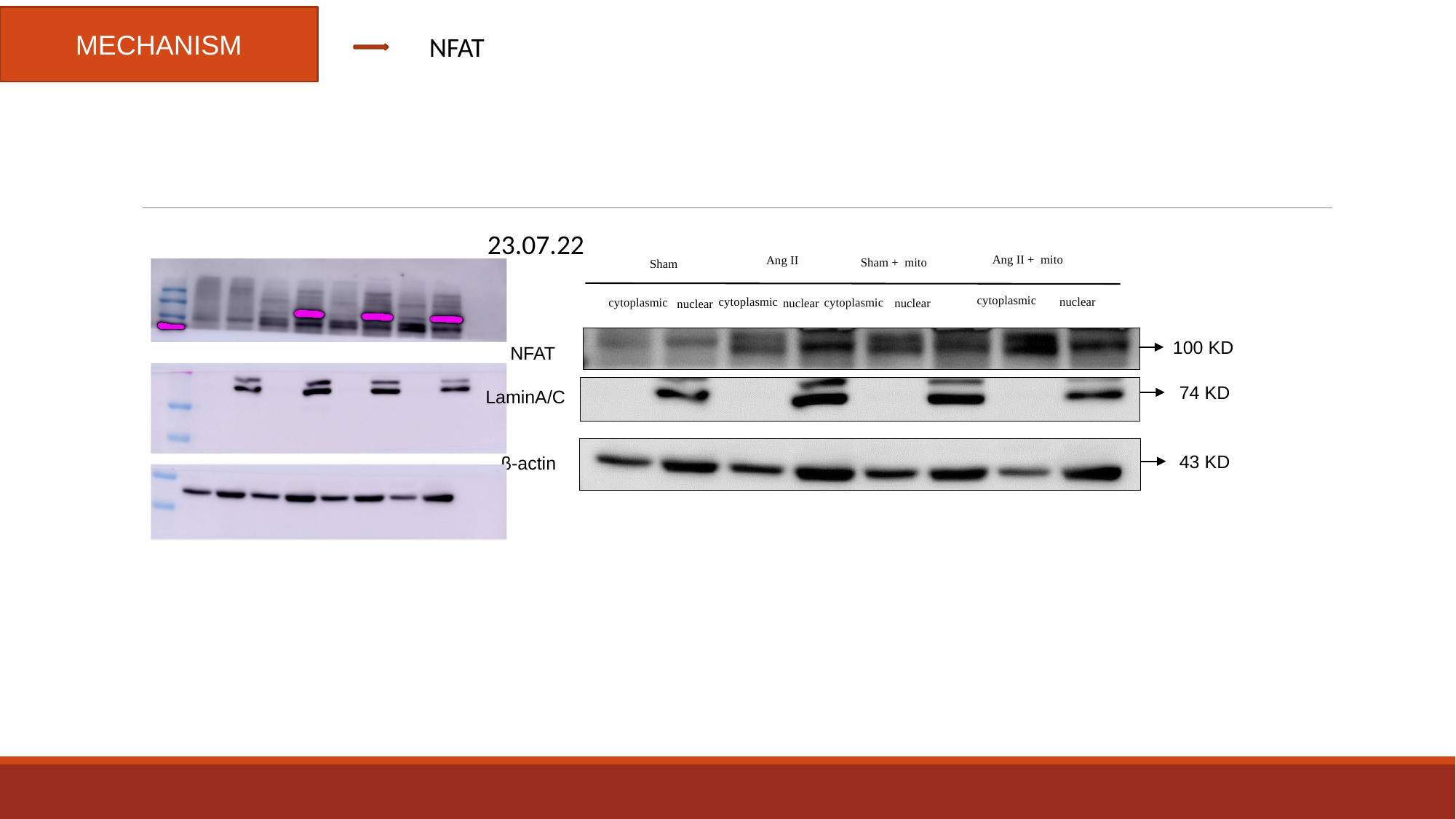

MECHANISM
NFAT
23.07.22
Ang II + mito
Ang II
Sham + mito
Sham
cytoplasmic
nuclear
cytoplasmic
cytoplasmic
cytoplasmic
nuclear
nuclear
nuclear
100 KD
NFAT
74 KD
LaminA/C
43 KD
β-actin

## Slide 17
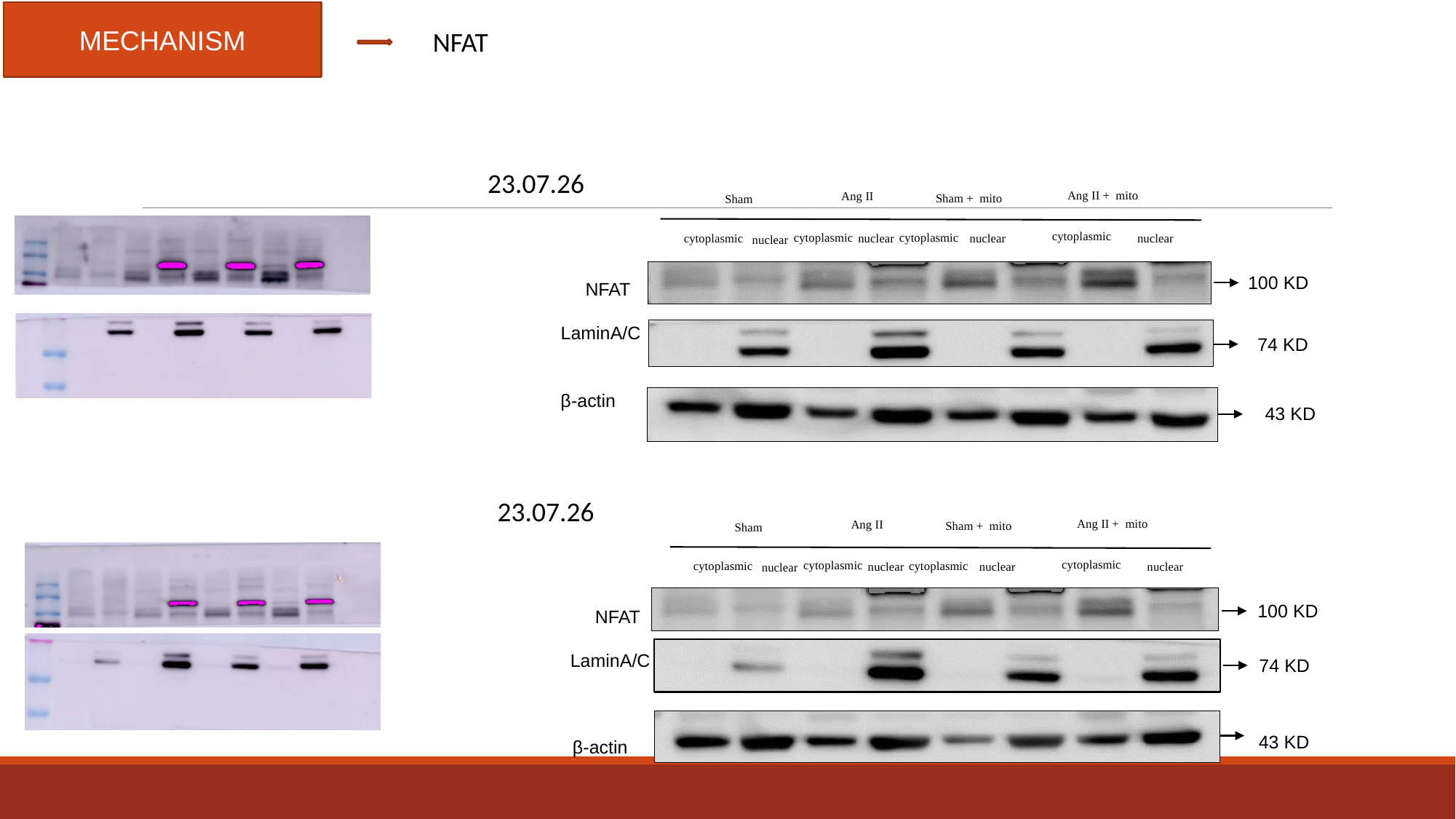

MECHANISM
NFAT
23.07.26
Ang II + mito
Ang II
Sham + mito
Sham
cytoplasmic
cytoplasmic
cytoplasmic
cytoplasmic
nuclear
nuclear
nuclear
nuclear
100 KD
NFAT
LaminA/C
74 KD
β-actin
43 KD
23.07.26
Ang II + mito
Ang II
Sham + mito
Sham
cytoplasmic
cytoplasmic
cytoplasmic
cytoplasmic
nuclear
nuclear
nuclear
nuclear
100 KD
NFAT
LaminA/C
74 KD
43 KD
β-actin

## Slide 18
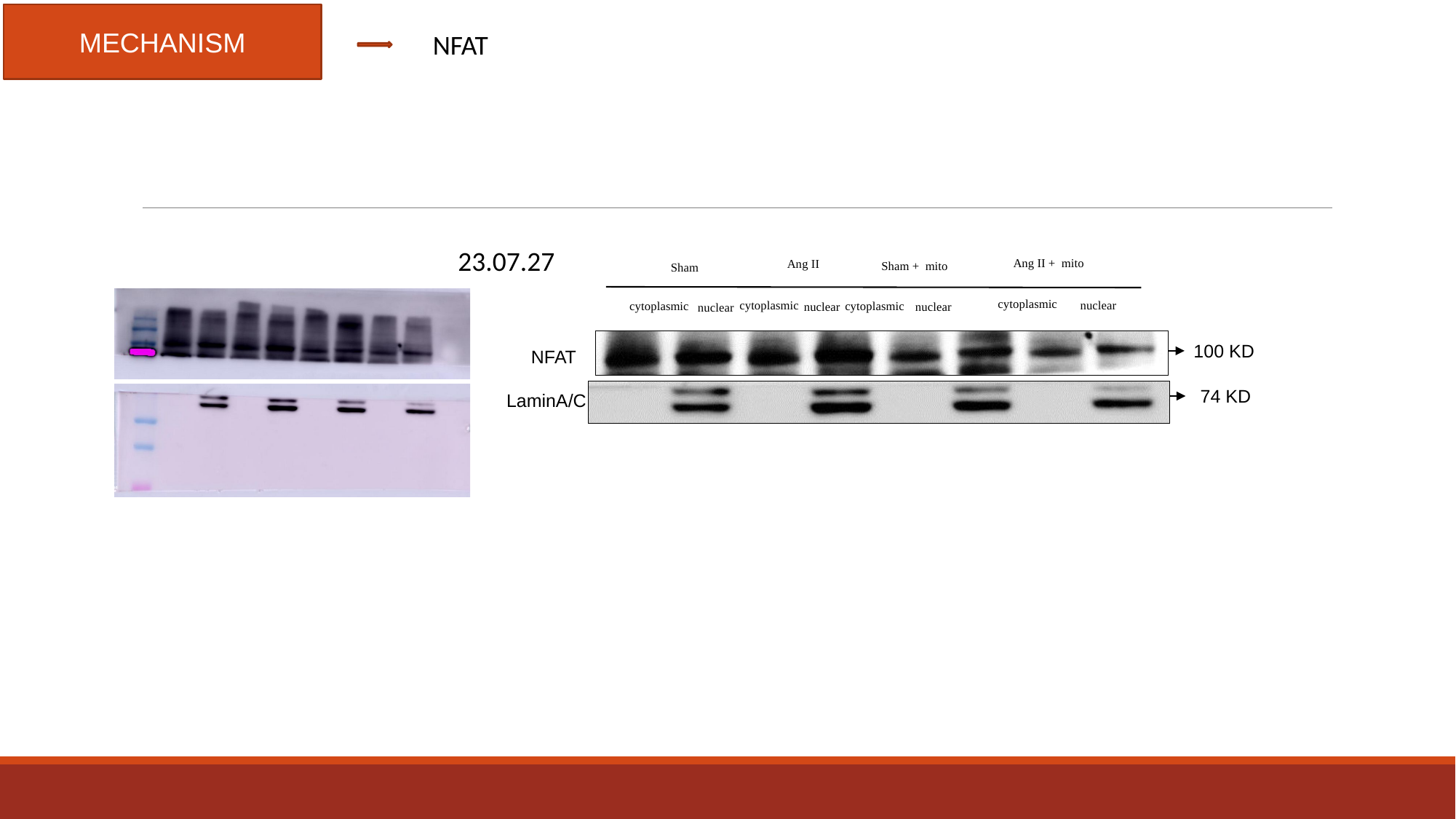

MECHANISM
NFAT
23.07.27
Ang II + mito
Ang II
Sham + mito
Sham
cytoplasmic
nuclear
cytoplasmic
cytoplasmic
cytoplasmic
nuclear
nuclear
nuclear
100 KD
NFAT
74 KD
LaminA/C

## Slide 19
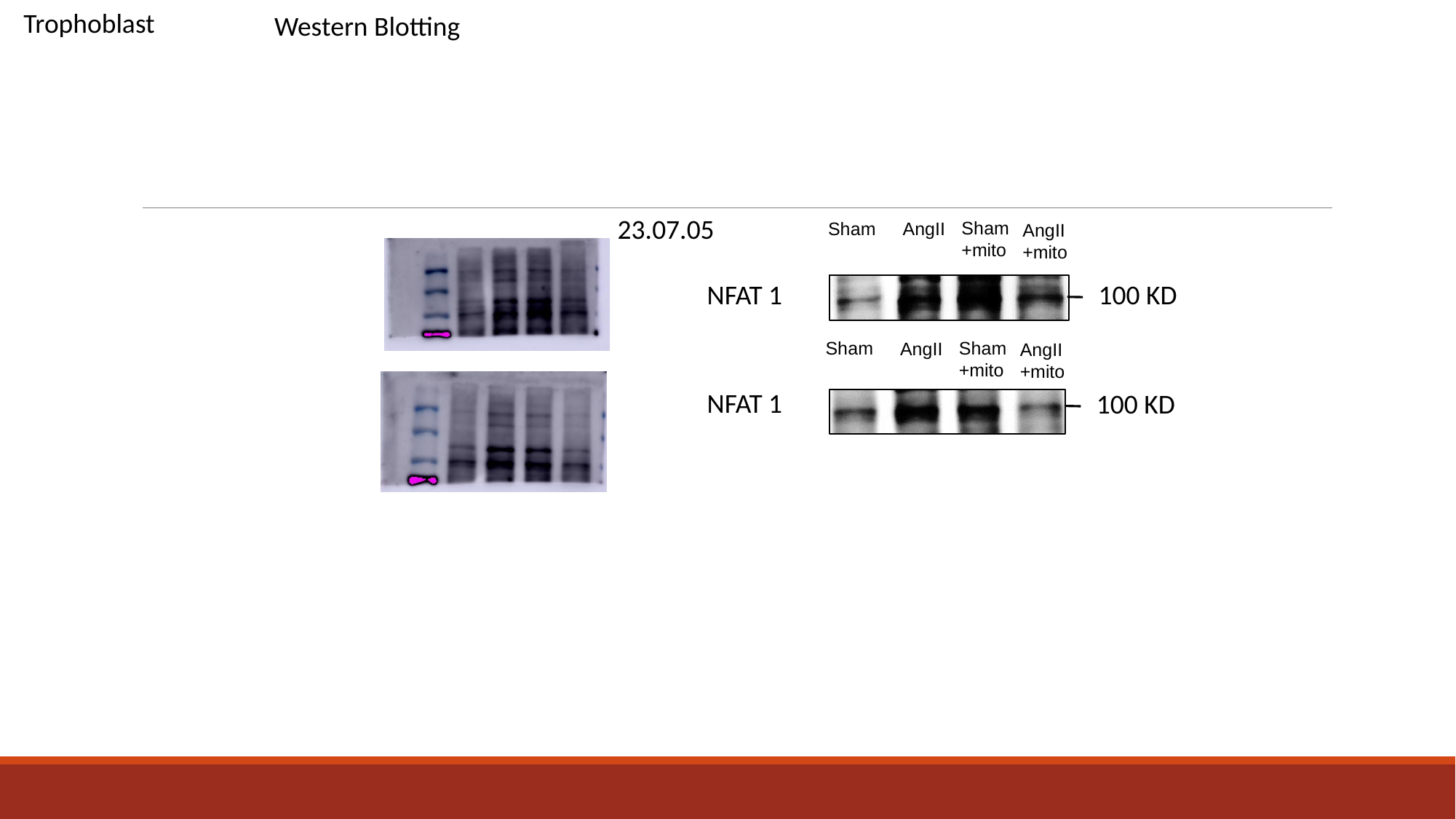

Trophoblast
Western Blotting
23.07.05
Sham
+mito
Sham
AngII
AngII
+mito
NFAT 1
100 KD
Sham
+mito
Sham
AngII
AngII
+mito
NFAT 1
100 KD

## Slide 20
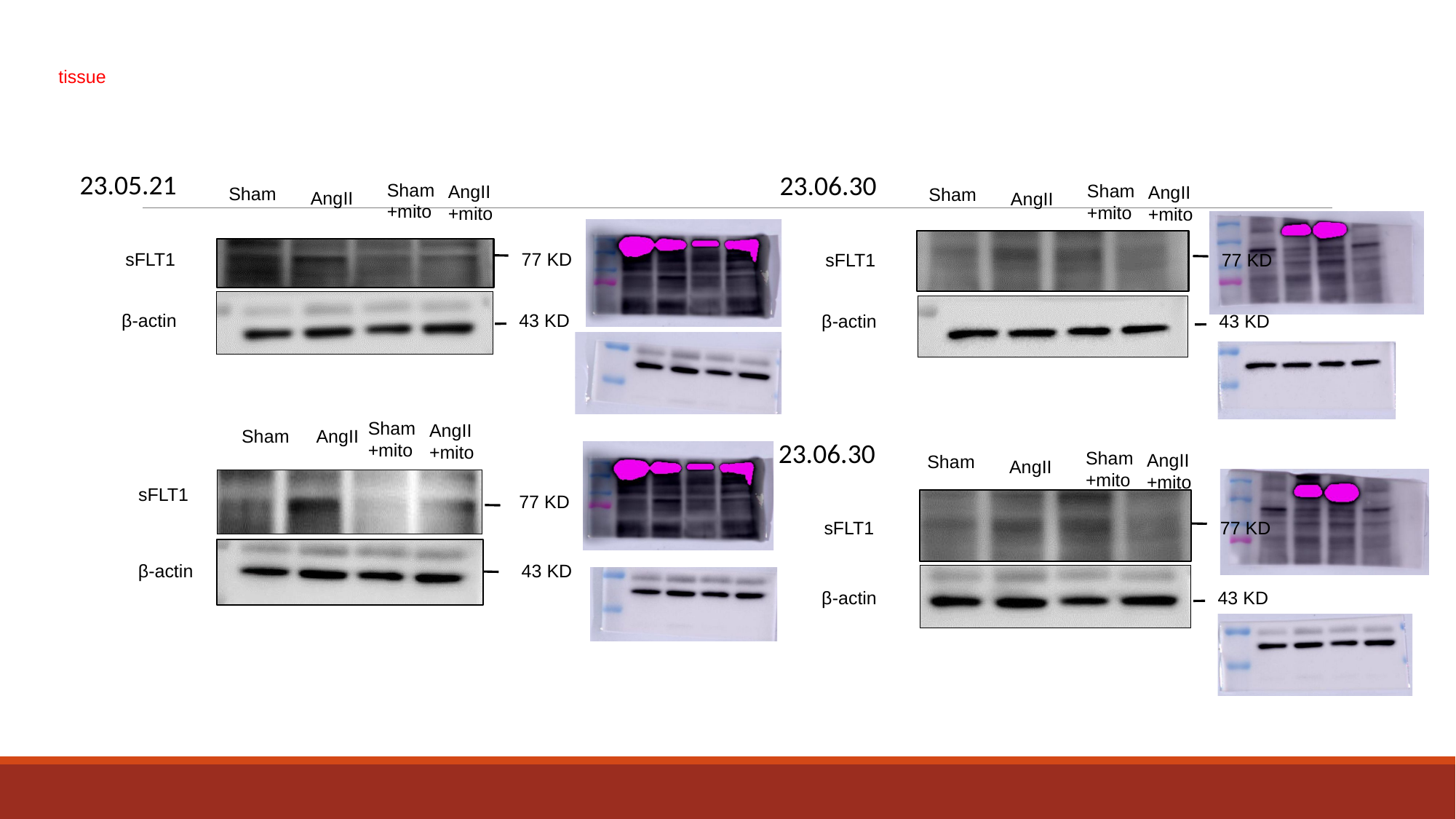

tissue
23.05.21
23.06.30
Sham
+mito
Sham
+mito
AngII
+mito
AngII
+mito
Sham
Sham
AngII
AngII
sFLT1
77 KD
sFLT1
77 KD
β-actin
43 KD
β-actin
43 KD
Sham
+mito
AngII
+mito
Sham
AngII
23.06.30
Sham
+mito
AngII
+mito
Sham
AngII
sFLT1
77 KD
sFLT1
77 KD
β-actin
43 KD
43 KD
β-actin

## Slide 21
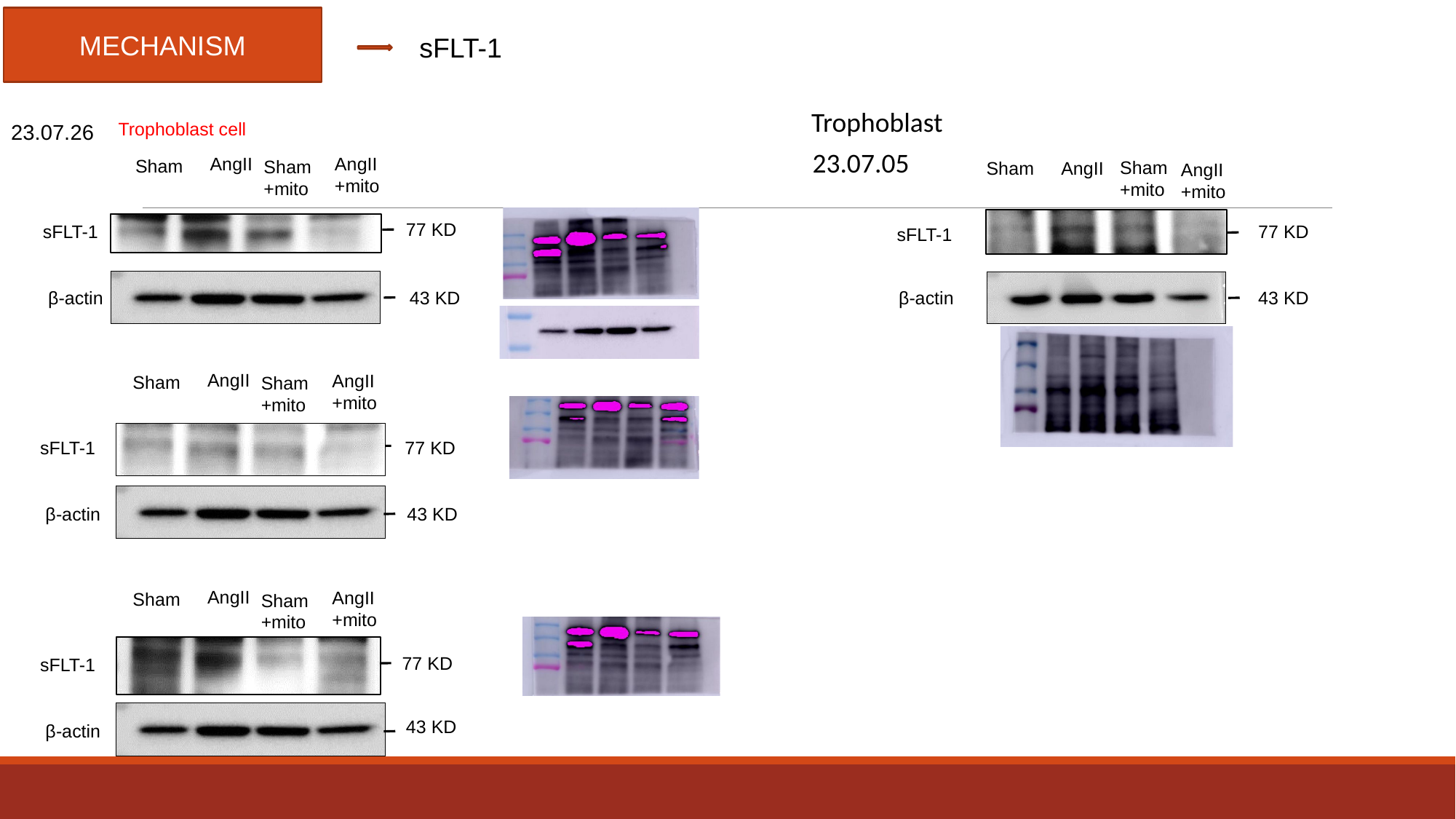

MECHANISM
sFLT-1
Trophoblast
Trophoblast cell
23.07.26
23.07.05
AngII
AngII
+mito
Sham
Sham
+mito
Sham
+mito
Sham
AngII
AngII
+mito
77 KD
sFLT-1
77 KD
sFLT-1
β-actin
43 KD
43 KD
β-actin
AngII
AngII
+mito
Sham
Sham
+mito
77 KD
sFLT-1
β-actin
43 KD
AngII
AngII
+mito
Sham
Sham
+mito
77 KD
sFLT-1
43 KD
β-actin

## Slide 22
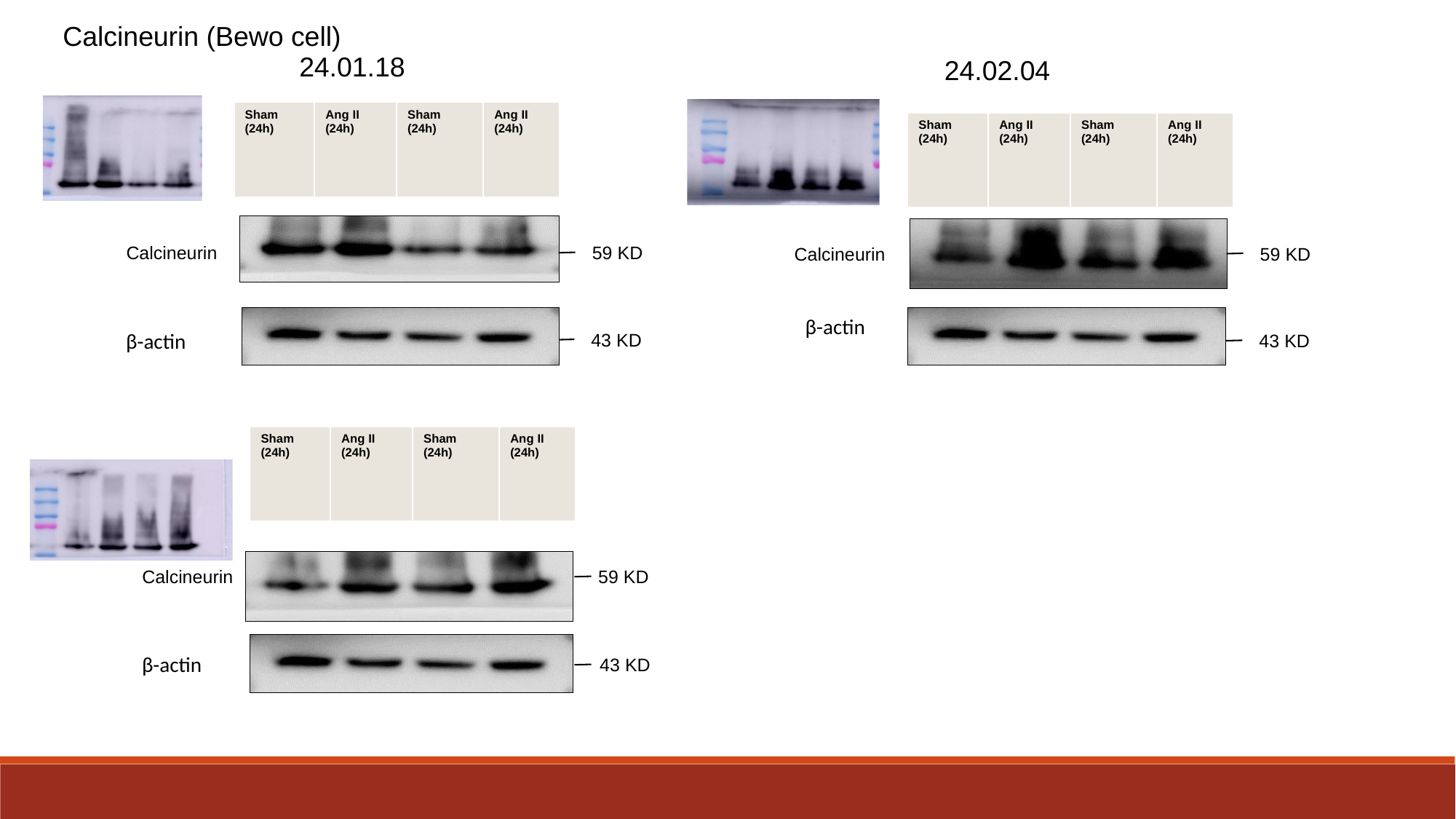

Calcineurin (Bewo cell)
24.01.18
24.02.04
| Sham (24h) | Ang II (24h) | Sham (24h) | Ang II (24h) |
| --- | --- | --- | --- |
| Sham (24h) | Ang II (24h) | Sham (24h) | Ang II (24h) |
| --- | --- | --- | --- |
Calcineurin
59 KD
Calcineurin
59 KD
β-actin
β-actin
43 KD
43 KD
| Sham (24h) | Ang II (24h) | Sham (24h) | Ang II (24h) |
| --- | --- | --- | --- |
Calcineurin
59 KD
β-actin
43 KD

## Slide 23
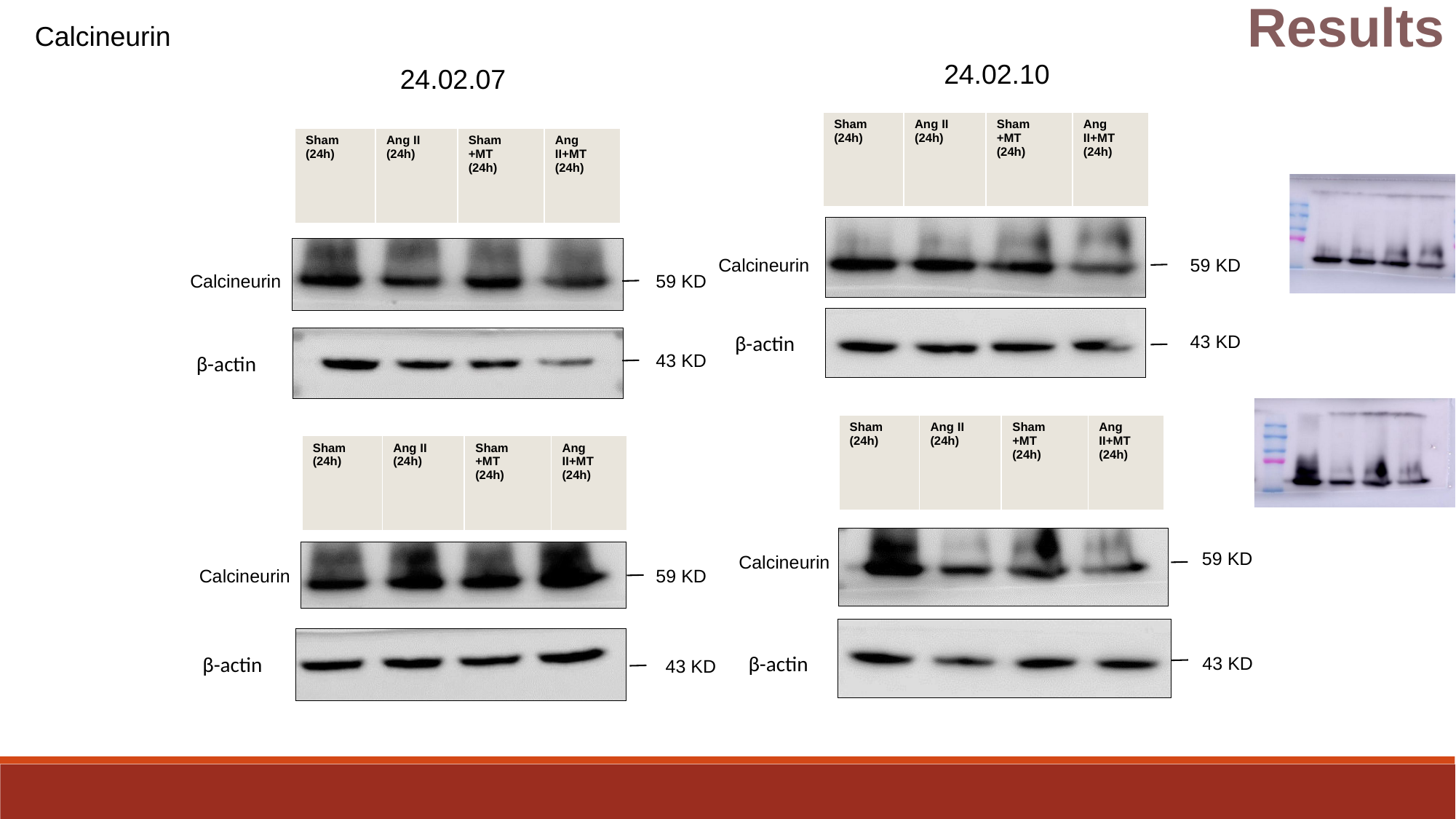

Results
Calcineurin
24.02.10
24.02.07
| Sham (24h) | Ang II (24h) | Sham +MT (24h) | Ang II+MT (24h) |
| --- | --- | --- | --- |
| Sham (24h) | Ang II (24h) | Sham +MT (24h) | Ang II+MT (24h) |
| --- | --- | --- | --- |
Calcineurin
59 KD
Calcineurin
59 KD
β-actin
43 KD
43 KD
β-actin
| Sham (24h) | Ang II (24h) | Sham +MT (24h) | Ang II+MT (24h) |
| --- | --- | --- | --- |
| Sham (24h) | Ang II (24h) | Sham +MT (24h) | Ang II+MT (24h) |
| --- | --- | --- | --- |
59 KD
Calcineurin
Calcineurin
59 KD
β-actin
β-actin
43 KD
43 KD

## Slide 24
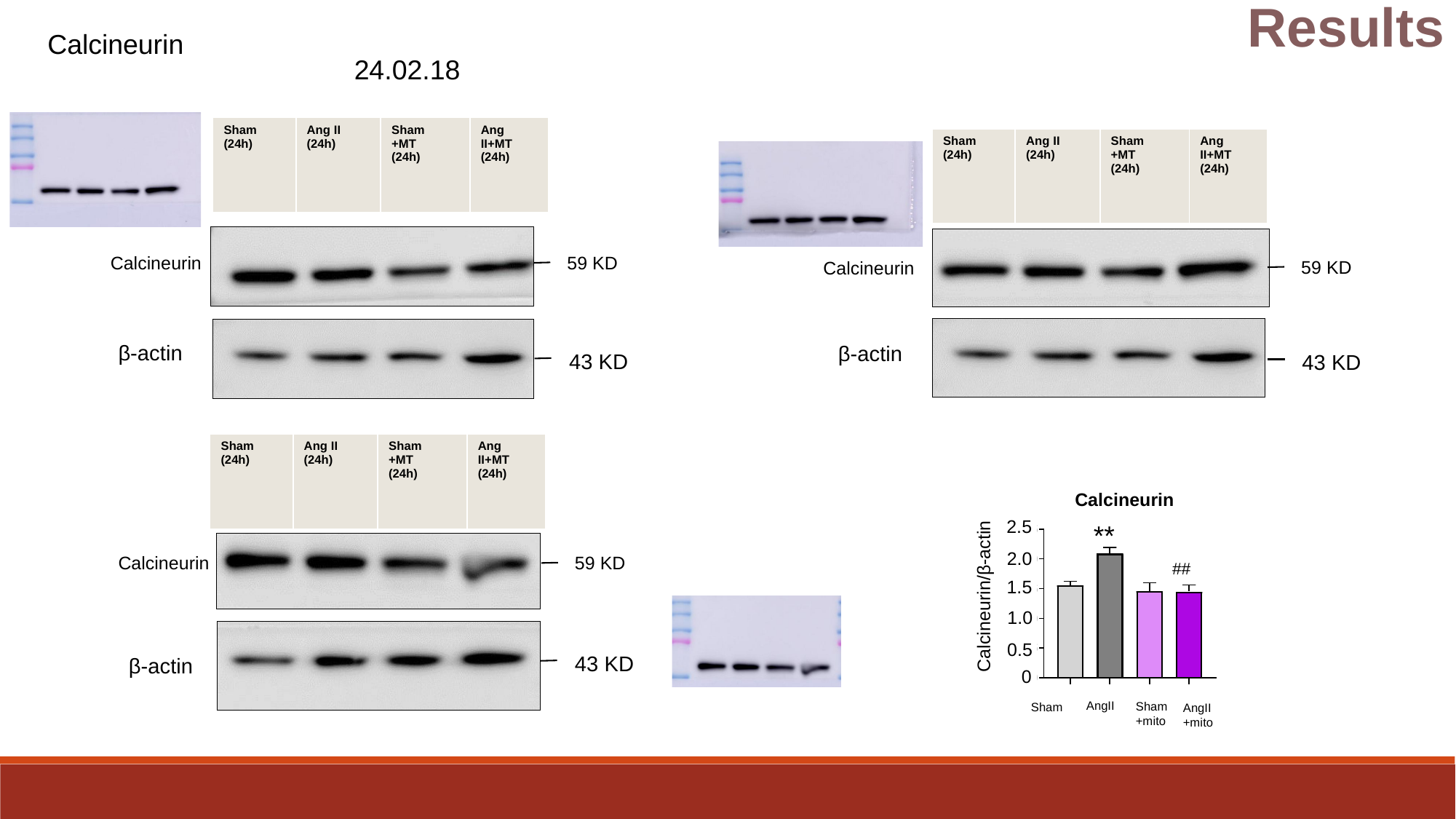

Results
Calcineurin
24.02.18
| Sham (24h) | Ang II (24h) | Sham +MT (24h) | Ang II+MT (24h) |
| --- | --- | --- | --- |
| Sham (24h) | Ang II (24h) | Sham +MT (24h) | Ang II+MT (24h) |
| --- | --- | --- | --- |
Calcineurin
59 KD
59 KD
Calcineurin
β-actin
β-actin
43 KD
43 KD
| Sham (24h) | Ang II (24h) | Sham +MT (24h) | Ang II+MT (24h) |
| --- | --- | --- | --- |
Calcineurin
2.5
**
2.0
##
1.5
1.0
0.5
0
Calcineurin
59 KD
Calcineurin/β-actin
43 KD
β-actin
AngII
Sham
+mito
Sham
AngII
+mito

## Slide 25
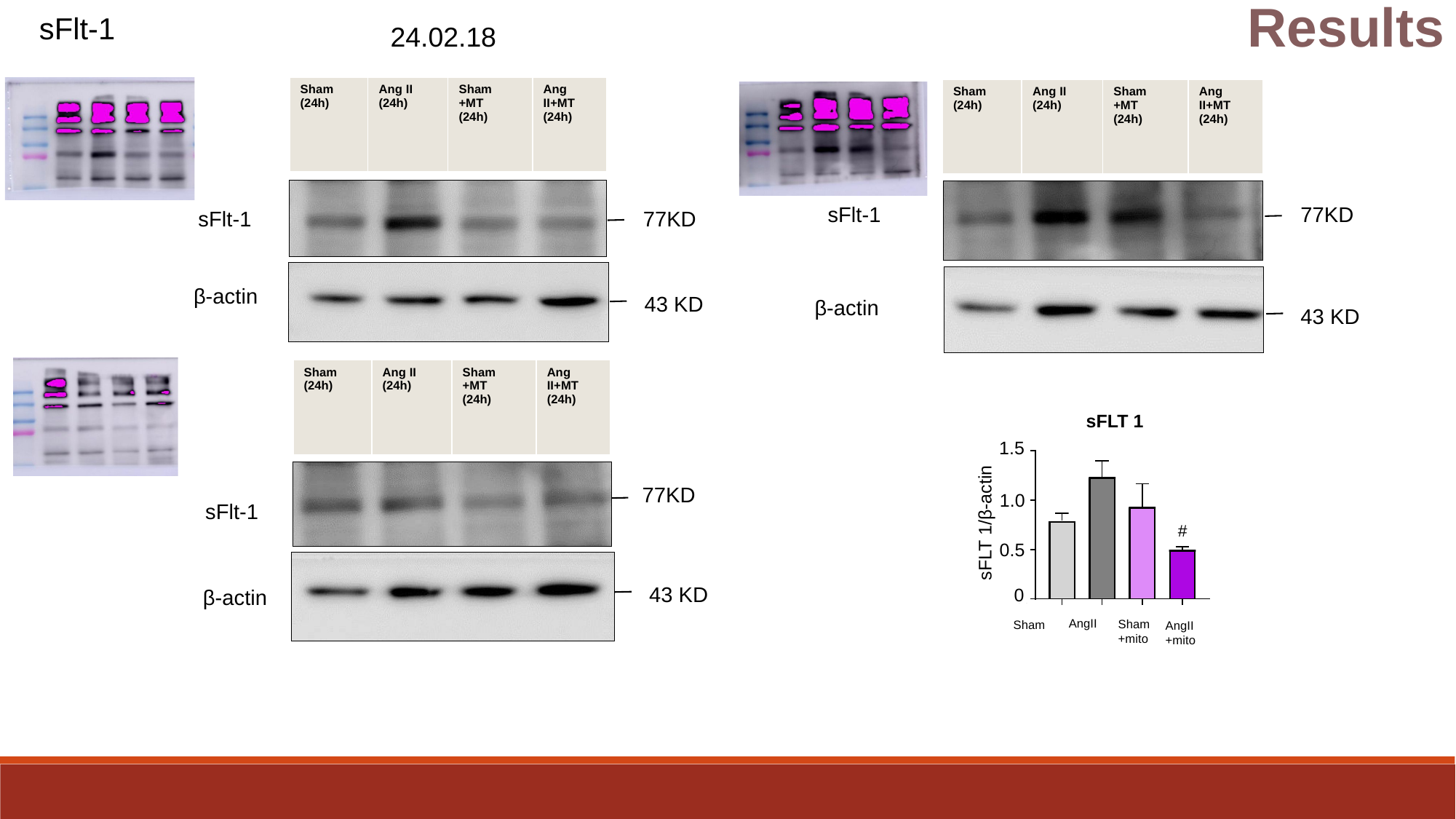

Results
sFlt-1
24.02.18
| Sham (24h) | Ang II (24h) | Sham +MT (24h) | Ang II+MT (24h) |
| --- | --- | --- | --- |
| Sham (24h) | Ang II (24h) | Sham +MT (24h) | Ang II+MT (24h) |
| --- | --- | --- | --- |
77KD
sFlt-1
77KD
sFlt-1
β-actin
43 KD
β-actin
43 KD
| Sham (24h) | Ang II (24h) | Sham +MT (24h) | Ang II+MT (24h) |
| --- | --- | --- | --- |
sFLT 1
1.5
1.0
#
0.5
0
77KD
sFlt-1
sFLT 1/β-actin
43 KD
β-actin
AngII
Sham
+mito
Sham
AngII
+mito

## Slide 26
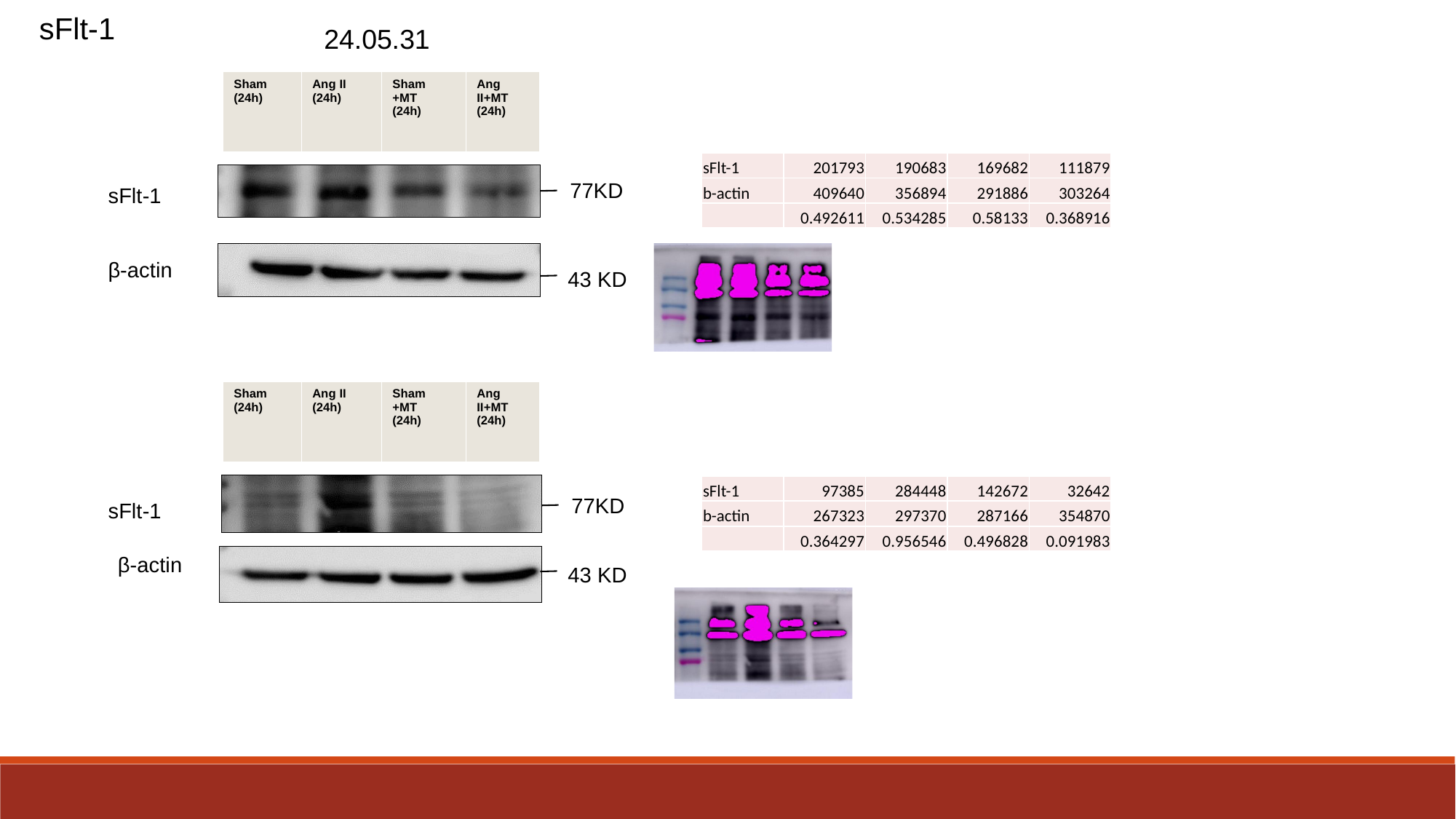

sFlt-1
24.05.31
| Sham (24h) | Ang II (24h) | Sham +MT (24h) | Ang II+MT (24h) |
| --- | --- | --- | --- |
| sFlt-1 | 201793 | 190683 | 169682 | 111879 |
| --- | --- | --- | --- | --- |
| b-actin | 409640 | 356894 | 291886 | 303264 |
| | 0.492611 | 0.534285 | 0.58133 | 0.368916 |
77KD
sFlt-1
β-actin
43 KD
| Sham (24h) | Ang II (24h) | Sham +MT (24h) | Ang II+MT (24h) |
| --- | --- | --- | --- |
| sFlt-1 | 97385 | 284448 | 142672 | 32642 |
| --- | --- | --- | --- | --- |
| b-actin | 267323 | 297370 | 287166 | 354870 |
| | 0.364297 | 0.956546 | 0.496828 | 0.091983 |
77KD
sFlt-1
β-actin
43 KD

## Slide 27
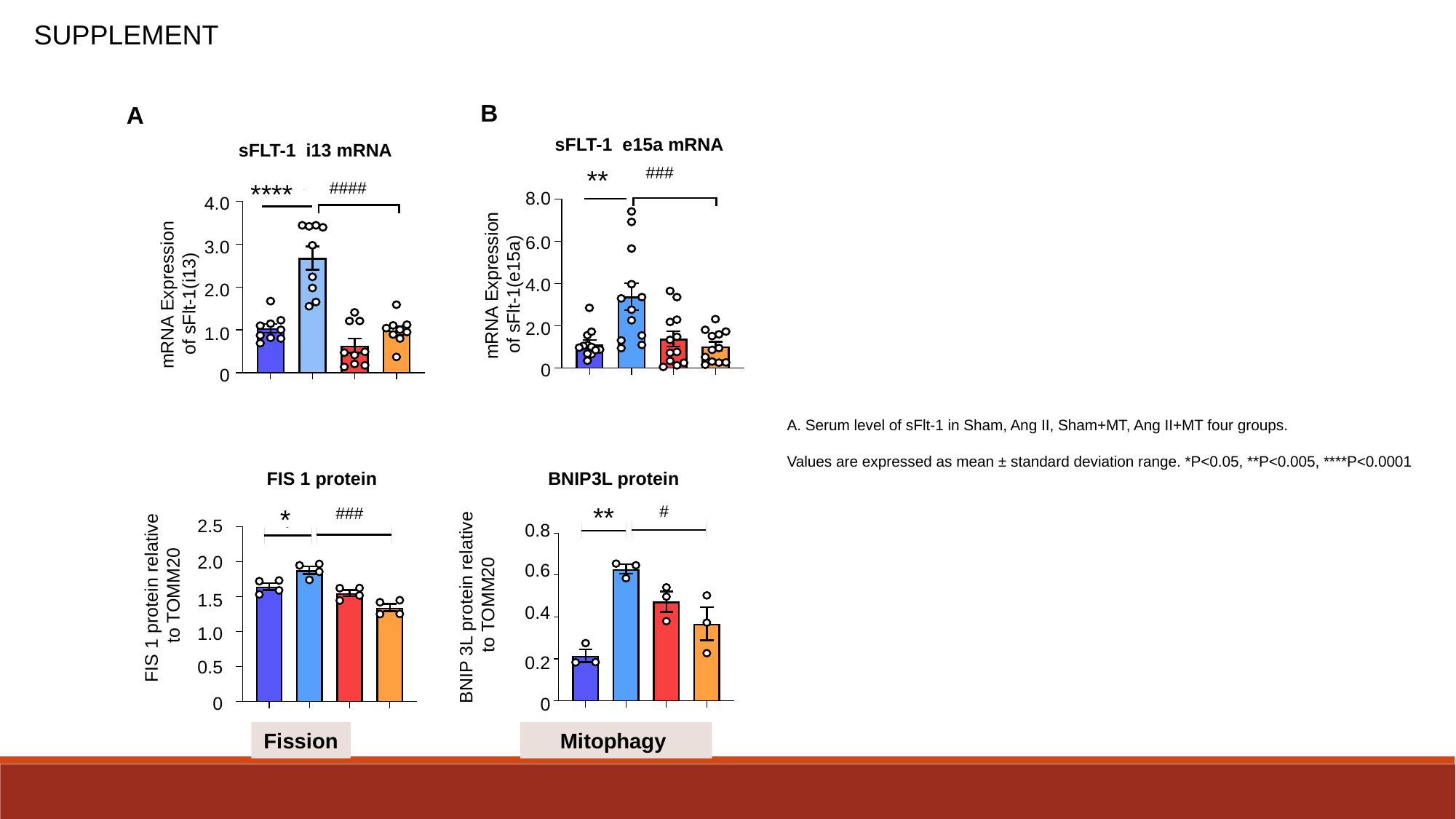

SUPPLEMENT
B
A
sFLT-1 e15a mRNA
###
**
8.0
6.0
mRNA Expression
of sFlt-1(e15a)
4.0
2.0
0
sFLT-1 i13 mRNA
****
####
4.0
3.0
mRNA Expression
of sFlt-1(i13)
2.0
1.0
0
A. Serum level of sFlt-1 in Sham, Ang II, Sham+MT, Ang II+MT four groups.
Values are expressed as mean ± standard deviation range. *P<0.05, **P<0.005, ****P<0.0001
FIS 1 protein
*
###
2.5
2.0
FIS 1 protein relative
to TOMM20
1.5
1.0
0.5
0
BNIP3L protein
**
#
0.8
0.6
BNIP 3L protein relative
to TOMM20
0.4
0.2
0
Fission
Mitophagy
