# Supplementary material for: Human umbilical mesenchymal stem cell-derived mitochondria transplantation suppresses sFLT-1 secretion by regulating calcineurin-NFAT-dependent pathways in angiotensin II-induced preeclampsia rats
Source: Stem Cell Res Ther. 2026 Feb 13;17:92. doi: 10.1186/s13287-026-04930-9 (PMC12954920; doi:10.1186/s13287-026-04930-9)
Supplement: Supplementary file 2 — Additional file 2 (PPTX 4863 KB) [file 13287_2026_4930_MOESM2_ESM.pptx]

## Slide 1
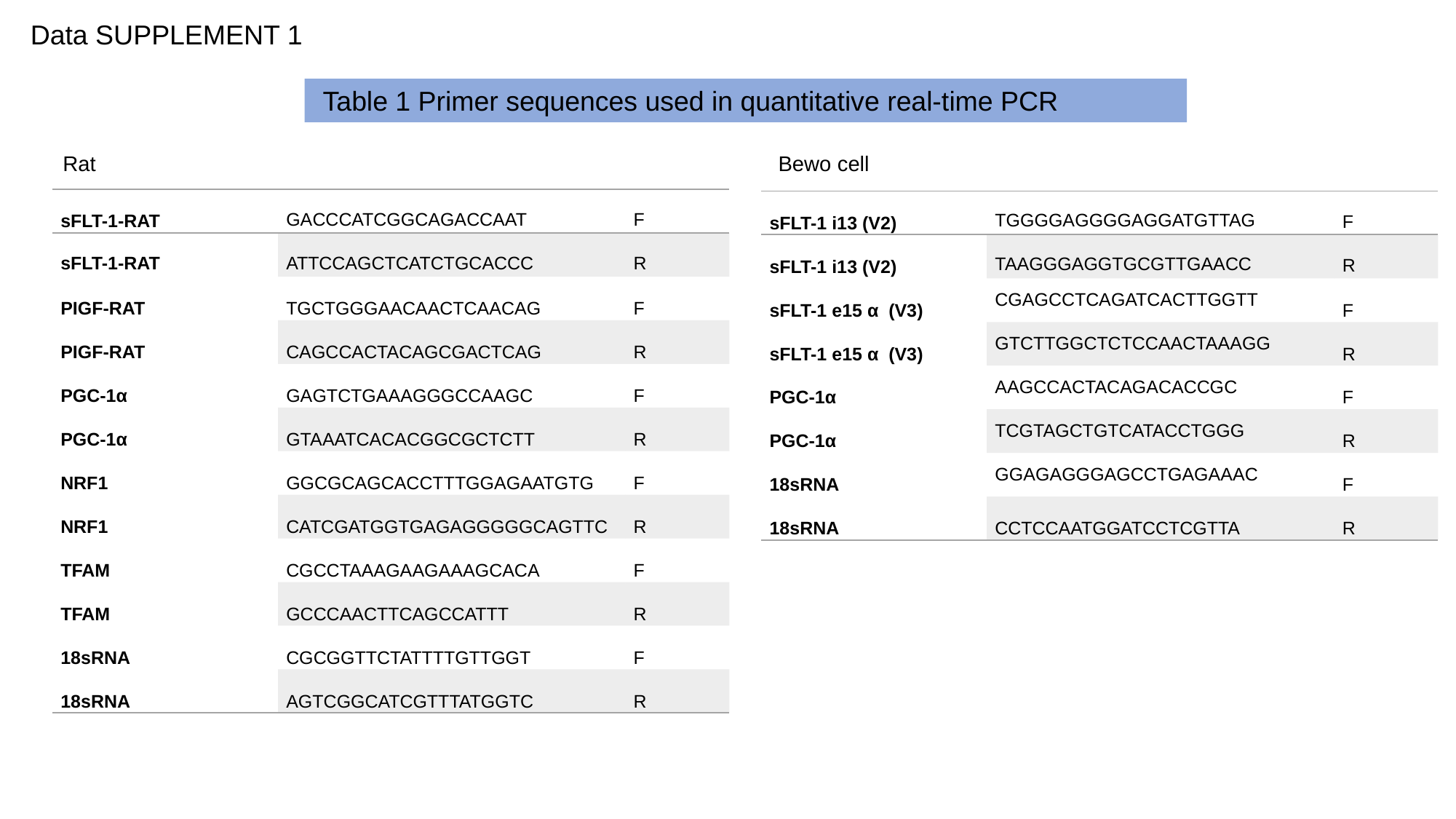

Data SUPPLEMENT 1
 Table 1 Primer sequences used in quantitative real-time PCR
Rat
Bewo cell
| sFLT-1-RAT | GACCCATCGGCAGACCAAT | F |
| --- | --- | --- |
| sFLT-1-RAT | ATTCCAGCTCATCTGCACCC | R |
| PIGF-RAT | TGCTGGGAACAACTCAACAG | F |
| PIGF-RAT | CAGCCACTACAGCGACTCAG | R |
| PGC-1α | GAGTCTGAAAGGGCCAAGC | F |
| PGC-1α | GTAAATCACACGGCGCTCTT | R |
| NRF1 | GGCGCAGCACCTTTGGAGAATGTG | F |
| NRF1 | CATCGATGGTGAGAGGGGGCAGTTC | R |
| TFAM | CGCCTAAAGAAGAAAGCACA | F |
| TFAM | GCCCAACTTCAGCCATTT | R |
| 18sRNA | CGCGGTTCTATTTTGTTGGT | F |
| 18sRNA | AGTCGGCATCGTTTATGGTC | R |
| sFLT-1 i13 (V2) | TGGGGAGGGGAGGATGTTAG | F |
| --- | --- | --- |
| sFLT-1 i13 (V2) | TAAGGGAGGTGCGTTGAACC | R |
| sFLT-1 e15 α (V3) | CGAGCCTCAGATCACTTGGTT | F |
| sFLT-1 e15 α (V3) | GTCTTGGCTCTCCAACTAAAGG | R |
| PGC-1α | AAGCCACTACAGACACCGC | F |
| PGC-1α | TCGTAGCTGTCATACCTGGG | R |
| 18sRNA | GGAGAGGGAGCCTGAGAAAC | F |
| 18sRNA | CCTCCAATGGATCCTCGTTA | R |

## Slide 2
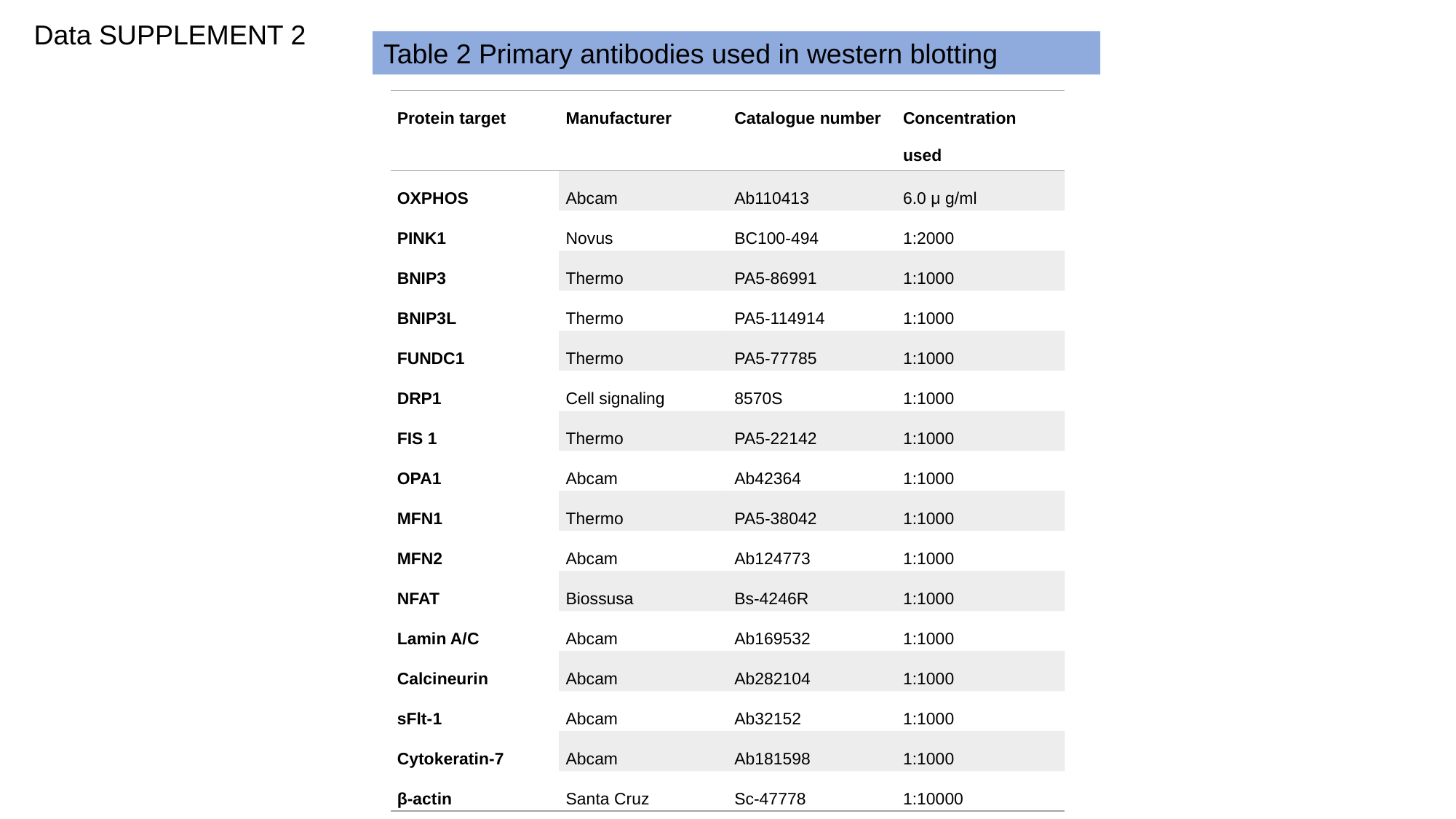

Data SUPPLEMENT 2
Table 2 Primary antibodies used in western blotting
| Protein target | Manufacturer | Catalogue number | Concentration used |
| --- | --- | --- | --- |
| OXPHOS | Abcam | Ab110413 | 6.0 μ g/ml |
| PINK1 | Novus | BC100-494 | 1:2000 |
| BNIP3 | Thermo | PA5-86991 | 1:1000 |
| BNIP3L | Thermo | PA5-114914 | 1:1000 |
| FUNDC1 | Thermo | PA5-77785 | 1:1000 |
| DRP1 | Cell signaling | 8570S | 1:1000 |
| FIS 1 | Thermo | PA5-22142 | 1:1000 |
| OPA1 | Abcam | Ab42364 | 1:1000 |
| MFN1 | Thermo | PA5-38042 | 1:1000 |
| MFN2 | Abcam | Ab124773 | 1:1000 |
| NFAT | Biossusa | Bs-4246R | 1:1000 |
| Lamin A/C | Abcam | Ab169532 | 1:1000 |
| Calcineurin | Abcam | Ab282104 | 1:1000 |
| sFlt-1 | Abcam | Ab32152 | 1:1000 |
| Cytokeratin-7 | Abcam | Ab181598 | 1:1000 |
| β-actin | Santa Cruz | Sc-47778 | 1:10000 |

## Slide 3
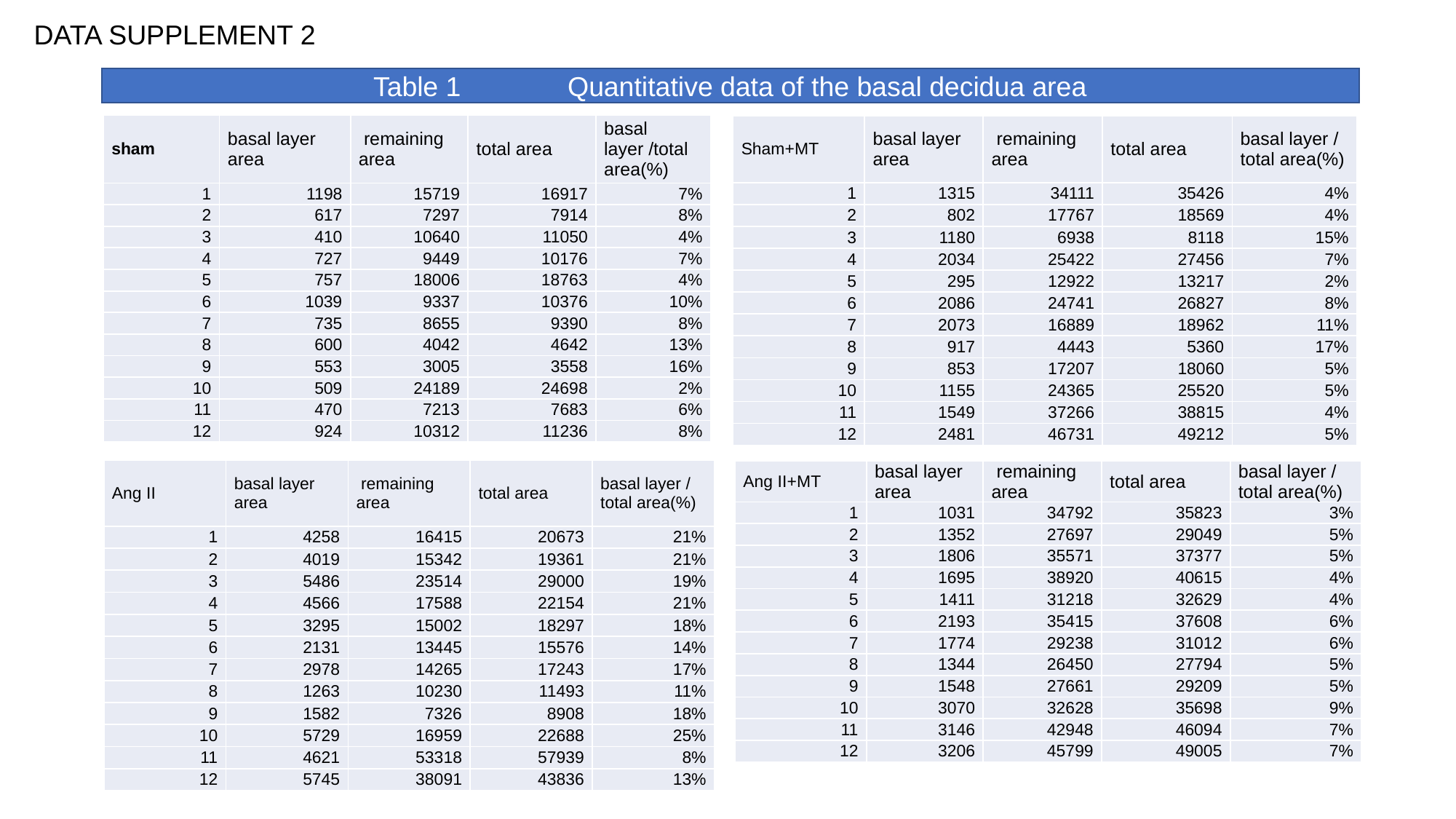

DATA SUPPLEMENT 2
Table 1 Quantitative data of the basal decidua area
| sham | basal layer area | remaining area | total area | basal layer /total area(%) |
| --- | --- | --- | --- | --- |
| 1 | 1198 | 15719 | 16917 | 7% |
| 2 | 617 | 7297 | 7914 | 8% |
| 3 | 410 | 10640 | 11050 | 4% |
| 4 | 727 | 9449 | 10176 | 7% |
| 5 | 757 | 18006 | 18763 | 4% |
| 6 | 1039 | 9337 | 10376 | 10% |
| 7 | 735 | 8655 | 9390 | 8% |
| 8 | 600 | 4042 | 4642 | 13% |
| 9 | 553 | 3005 | 3558 | 16% |
| 10 | 509 | 24189 | 24698 | 2% |
| 11 | 470 | 7213 | 7683 | 6% |
| 12 | 924 | 10312 | 11236 | 8% |
| Sham+MT | basal layer area | remaining area | total area | basal layer /total area(%) |
| --- | --- | --- | --- | --- |
| 1 | 1315 | 34111 | 35426 | 4% |
| 2 | 802 | 17767 | 18569 | 4% |
| 3 | 1180 | 6938 | 8118 | 15% |
| 4 | 2034 | 25422 | 27456 | 7% |
| 5 | 295 | 12922 | 13217 | 2% |
| 6 | 2086 | 24741 | 26827 | 8% |
| 7 | 2073 | 16889 | 18962 | 11% |
| 8 | 917 | 4443 | 5360 | 17% |
| 9 | 853 | 17207 | 18060 | 5% |
| 10 | 1155 | 24365 | 25520 | 5% |
| 11 | 1549 | 37266 | 38815 | 4% |
| 12 | 2481 | 46731 | 49212 | 5% |
| Ang II | basal layer area | remaining area | total area | basal layer /total area(%) |
| --- | --- | --- | --- | --- |
| 1 | 4258 | 16415 | 20673 | 21% |
| 2 | 4019 | 15342 | 19361 | 21% |
| 3 | 5486 | 23514 | 29000 | 19% |
| 4 | 4566 | 17588 | 22154 | 21% |
| 5 | 3295 | 15002 | 18297 | 18% |
| 6 | 2131 | 13445 | 15576 | 14% |
| 7 | 2978 | 14265 | 17243 | 17% |
| 8 | 1263 | 10230 | 11493 | 11% |
| 9 | 1582 | 7326 | 8908 | 18% |
| 10 | 5729 | 16959 | 22688 | 25% |
| 11 | 4621 | 53318 | 57939 | 8% |
| 12 | 5745 | 38091 | 43836 | 13% |
| Ang II+MT | basal layer area | remaining area | total area | basal layer /total area(%) |
| --- | --- | --- | --- | --- |
| 1 | 1031 | 34792 | 35823 | 3% |
| 2 | 1352 | 27697 | 29049 | 5% |
| 3 | 1806 | 35571 | 37377 | 5% |
| 4 | 1695 | 38920 | 40615 | 4% |
| 5 | 1411 | 31218 | 32629 | 4% |
| 6 | 2193 | 35415 | 37608 | 6% |
| 7 | 1774 | 29238 | 31012 | 6% |
| 8 | 1344 | 26450 | 27794 | 5% |
| 9 | 1548 | 27661 | 29209 | 5% |
| 10 | 3070 | 32628 | 35698 | 9% |
| 11 | 3146 | 42948 | 46094 | 7% |
| 12 | 3206 | 45799 | 49005 | 7% |

## Slide 4
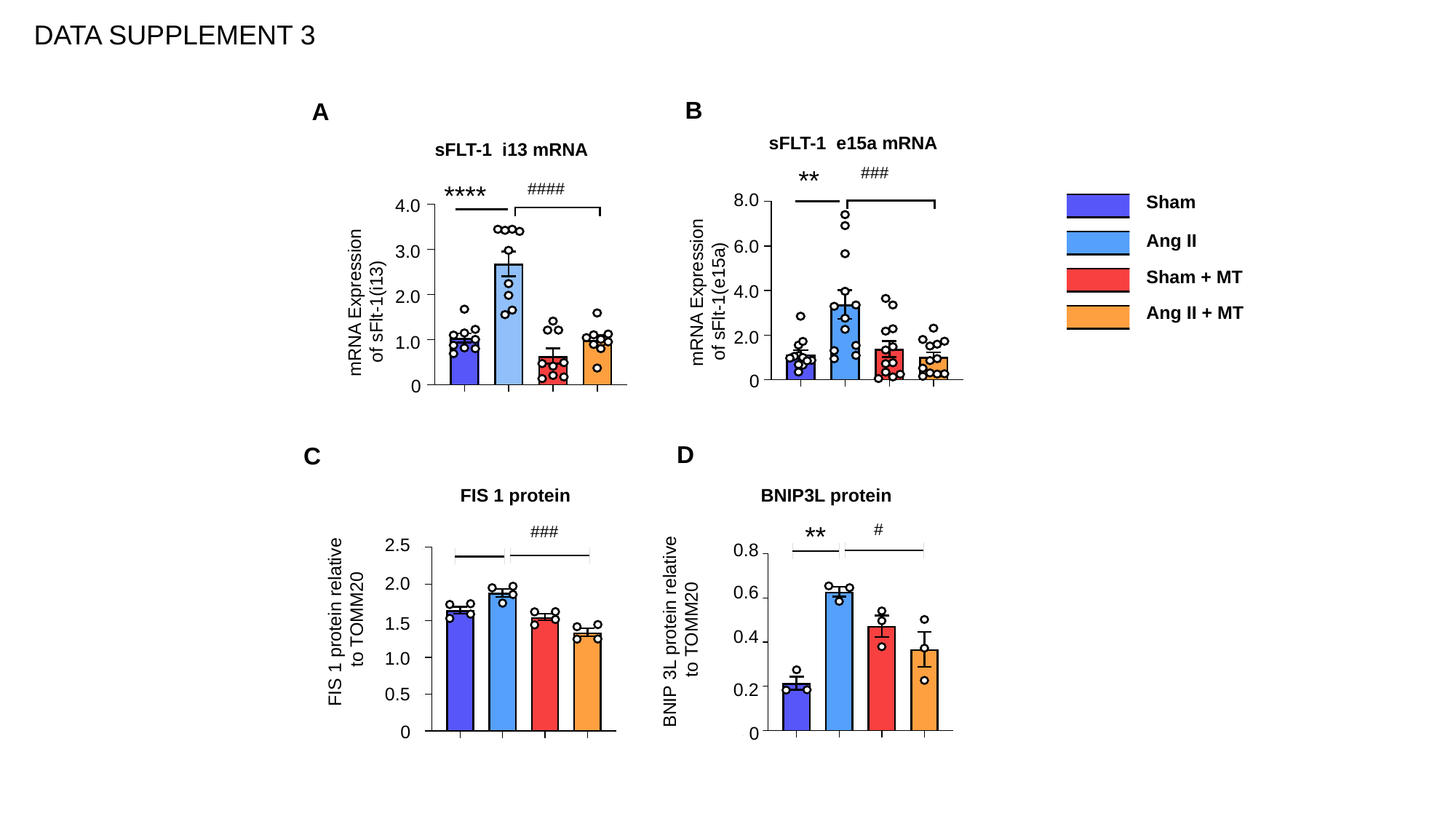

DATA SUPPLEMENT 3
This should be in figure legends
B
A
sFLT-1 e15a mRNA
###
**
8.0
6.0
mRNA Expression
of sFlt-1(e15a)
4.0
2.0
0
sFLT-1 i13 mRNA
****
####
4.0
3.0
mRNA Expression
of sFlt-1(i13)
2.0
1.0
0
Sham
Ang II
Sham + MT
Ang II + MT
D
C
FIS 1 protein
BNIP3L protein
**
#
0.8
0.6
BNIP 3L protein relative
to TOMM20
0.4
0.2
0
*
###
2.5
2.0
FIS 1 protein relative
to TOMM20
1.5
1.0
0.5
0
sFlt-1 i13 mRNA expression in Sham, Ang II, Sham+MT, Ang II+MT four groups in Bewo cells.
 sFlt-1 e15a mRNA expression in Sham, Ang II, Sham+MT, Ang II+MT four groups in Bewo cells. sFlt-1 i13 and sFlt-1 e15a are two subtypes of sFlt-1.
FIS 1protein expression in Sham, Ang II, Sham+MT, Ang II+MT four groups in Bewo cells. This result is consistent with the findings from the previous placental tissue samples.
BNIP3L protein expression in Sham, Ang II, Sham+MT, Ang II+MT four groups in Bewo cells. This result is consistent with the findings from the previous placental tissue samples.
Values are expressed as mean ± standard deviation range. *P<0.05, **P<0.005, ****P<0.0001

## Slide 5
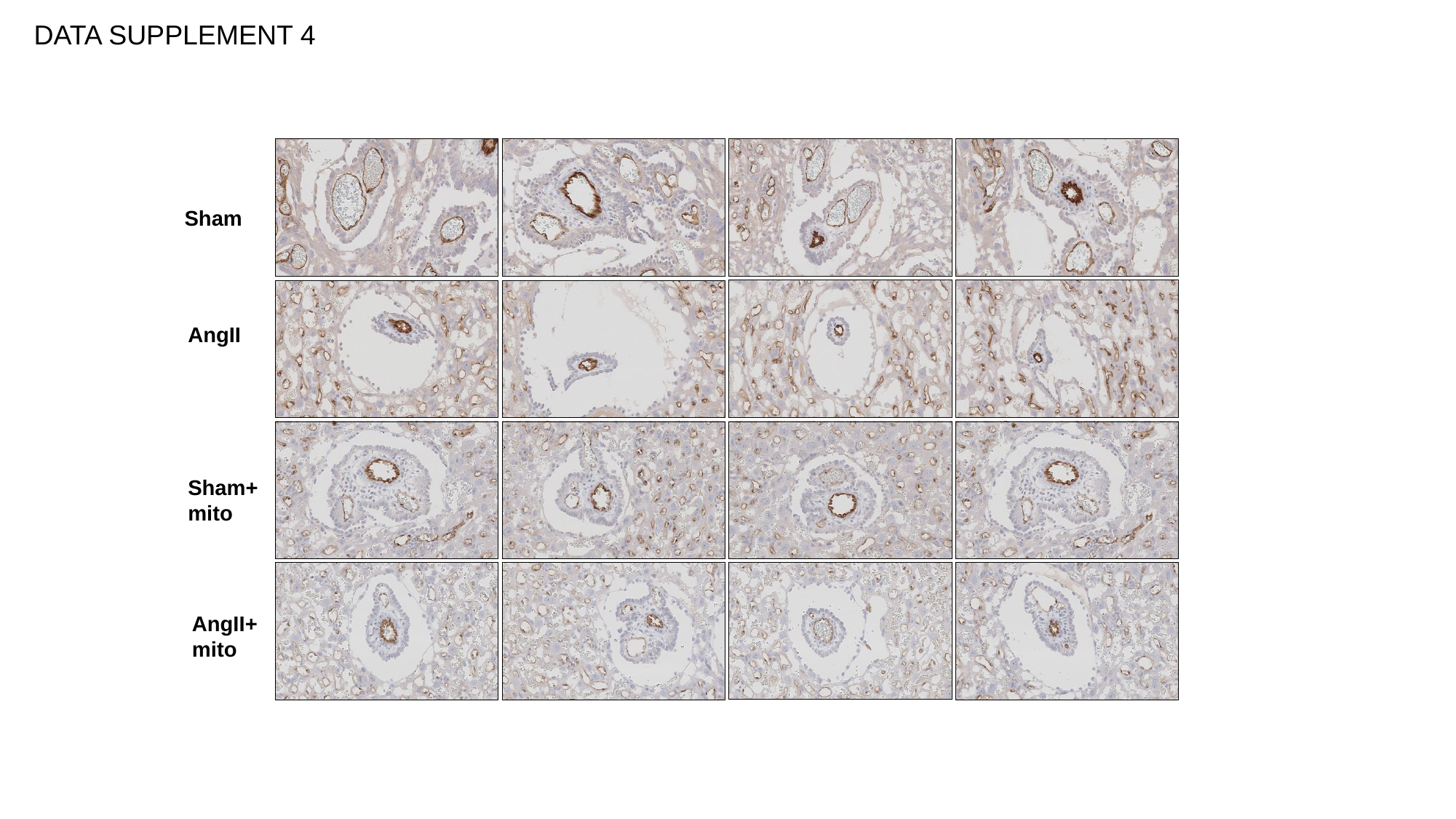

DATA SUPPLEMENT 4
Sham
AngII
Sham+mito
AngII+mito

## Slide 6
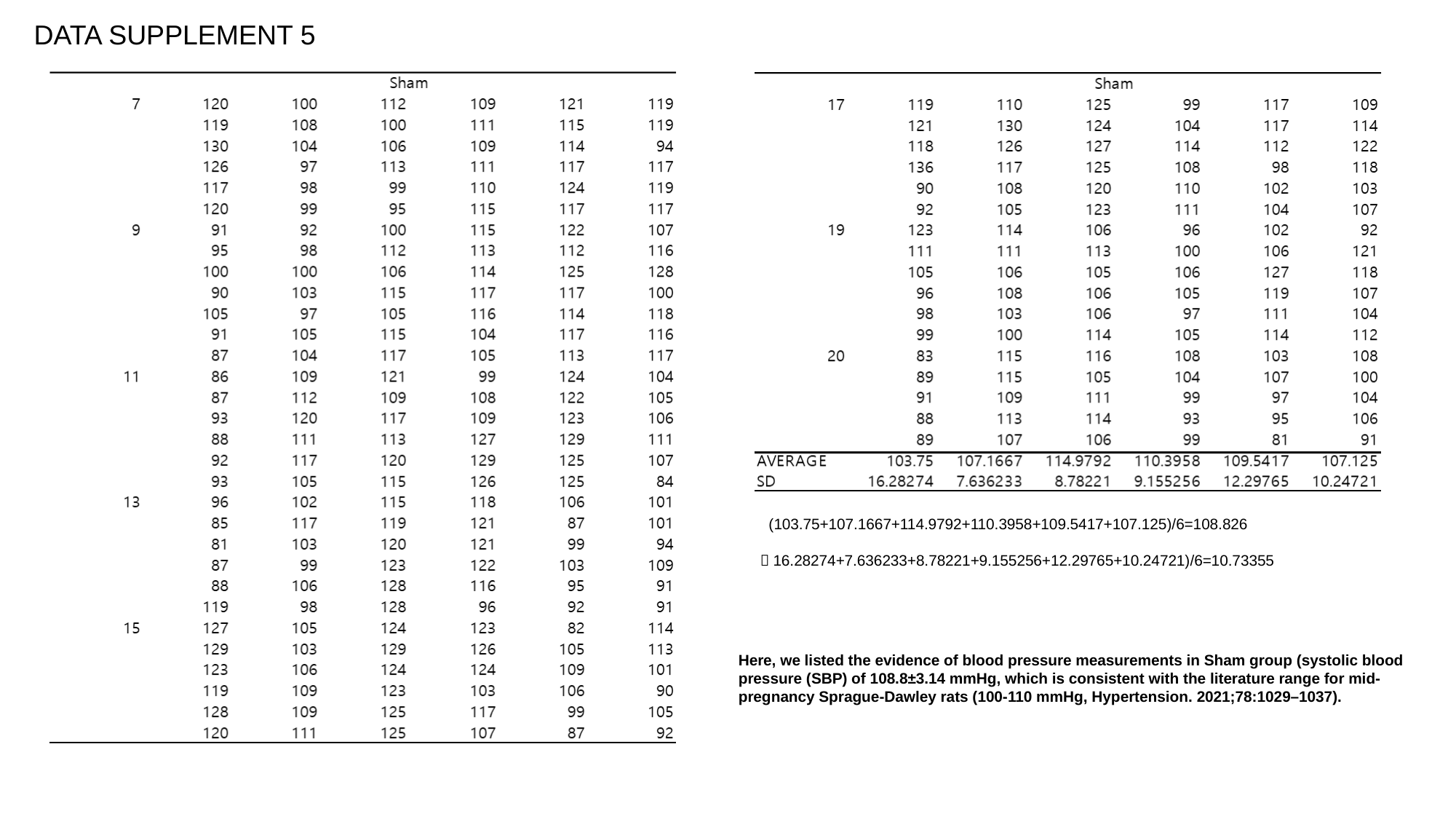

DATA SUPPLEMENT 5
 (103.75+107.1667+114.9792+110.3958+109.5417+107.125)/6=108.826
（16.28274+7.636233+8.78221+9.155256+12.29765+10.24721)/6=10.73355
Here, we listed the evidence of blood pressure measurements in Sham group (systolic blood pressure (SBP) of 108.8±3.14 mmHg, which is consistent with the literature range for mid-pregnancy Sprague-Dawley rats (100-110 mmHg, Hypertension. 2021;78:1029–1037).
