# Supplementary material for: Human umbilical mesenchymal stem cell-derived mitochondria transplantation suppresses sFLT-1 secretion by regulating calcineurin-NFAT-dependent pathways in angiotensin II-induced preeclampsia rats
Source: Stem Cell Res Ther. 2026 Feb 13;17:92. doi: 10.1186/s13287-026-04930-9 (PMC12954920; doi:10.1186/s13287-026-04930-9)
Supplement: Supplementary file 3 — Additional file 3 (DOCX 19 KB) [file 13287_2026_4930_MOESM3_ESM.docx]

**Data supplement 1**

Primer sequences used for quantitative real-time PCR

**Data supplement 2**

Primary antibodies used for western blotting

**Data supplement 3**

1. sFlt-1 i13 mRNA expression in the Sham, Ang II, Sham+MT, and Ang II+MT groups in BeWo cells.
2. sFlt-1 e15a mRNA expression in the Sham, Ang II, Sham+MT, and Ang II+MT groups in BeWo cells. sFlt-1 i13 and sFlt-1 e15a are two subtypes of sFlt-1.
3. FIS 1protein expression in the Sham, Ang II, Sham+MT, and Ang II+MT groups in BeWo cells, consistent with the findings from previous placental tissue samples.
4. BNIP3L protein expression in the Sham, Ang II, Sham+MT, and Ang II+MT groups in BeWo cells, consistent with the findings from previous placental tissue samples.

Values are expressed as mean ± standard deviation. *P<0.05, **P<0.005, ****P<0.0001

**Data supplement 4**

Quantification of perivascular cells in the chorionic villi from highlighted placental regions

1. Representative images selected regions
2. Table showing cell counts for each group.

**Data supplement 5**

Blood pressure measurements in the Sham group (systolic blood pressure (SBP) was 108.8±3.14 mmHg, consistent with the literature values for mid-pregnancy Sprague-Dawley rats (100‒110 mmHg, Hypertension)
